# Supplementary material for: A simple method for developing lysine targeted covalent protein reagents
Source: Nat Commun. 2023 Dec 1;14:7933. doi: 10.1038/s41467-023-42632-5 (PMC10692228; doi:10.1038/s41467-023-42632-5)
Supplement: Supplementary file 1 — Supplementary Information [file 41467_2023_42632_MOESM1_ESM.pdf]

Supplementary information

A simple method for developing lysine targeted covalent protein reagents

Ronen Gabizon[1*], Barr Tivon[1*], Rambabu N. Reddi[1], Maxime C. M. van den Oetelaar[2], Hadar Amartely[3], Peter J. Cossar[2], Christian Ottmann[2], Nir London[1,#]

[1] Department of Chemical and Structural Biology, The Weizmann Institute of Science, Rehovot, Israel, 7610001

[2] Laboratory of Chemical Biology, Department of Biomedical Engineering, Institute for Complex Molecular Systems, Eindhoven University of Technology, P.O. Box 513, 5600MB Eindhoven, The Netherlands

[3] Wolfson Centre for Applied Structural Biology, The Alexander Silberman Institute of Life Sciences, The Hebrew University of Jerusalem, Jerusalem, 9190401, Israel

[*] Equal contribution

[#] To whom correspondence should be addressed: N.L. nir.london@weizmann.ac.il

Keywords: irreversible binding; protein-protein interactions; methacrylamides; covalent drugs; bioconjugation

Supplementary Figures

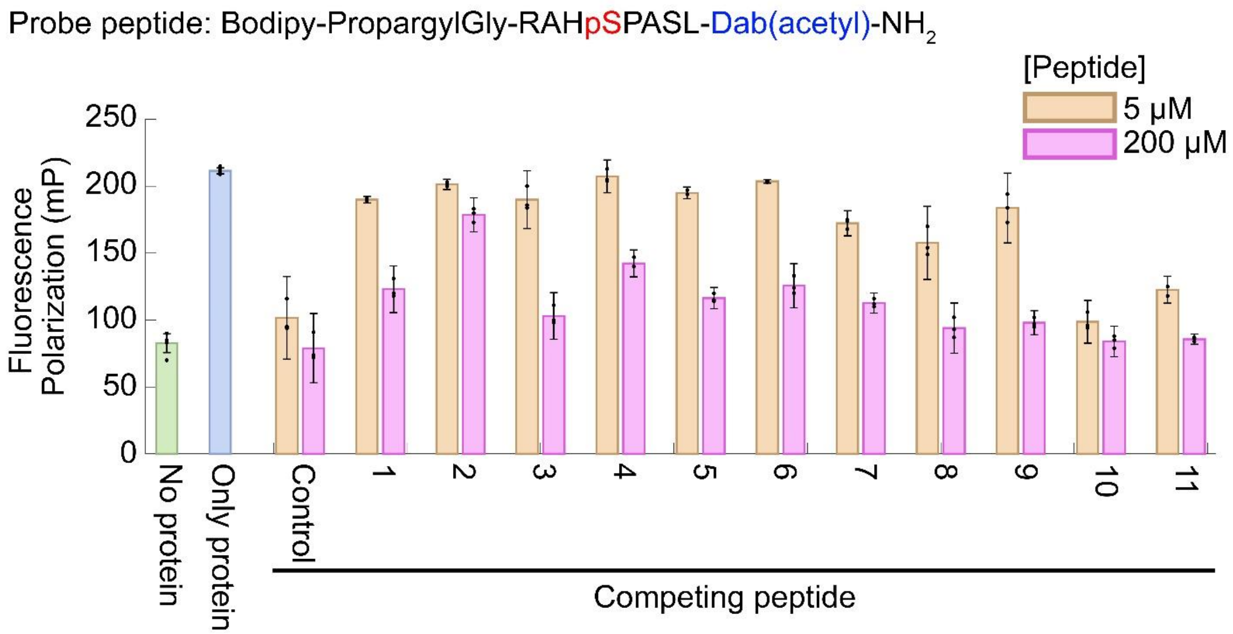

Supplementary Figure 1. Fluorescence Polarization competition experiments.
Fluorescence polarization of 5 nM BODIPY-labeled noncovalent binder for 14-3-3σ was measured alone (green), in the presence of 0.25 μM 14-3-3σ (blue) and in the presence of 14-3-3σ and the different electrophilic peptides (orange and magenta). Black dots indicate data from individual samples, bars represent average values. Error bars represent 95% confidence interval calculated using students T test from n = 3 samples, except for the “no protein” and “only protein” data, in which there were n = 6 samples. The sequence of the BDP labeled binder is shown on top, Dab = diaminobutyric acid. The control peptide is identical to the BDP-labeled binder in sequence.

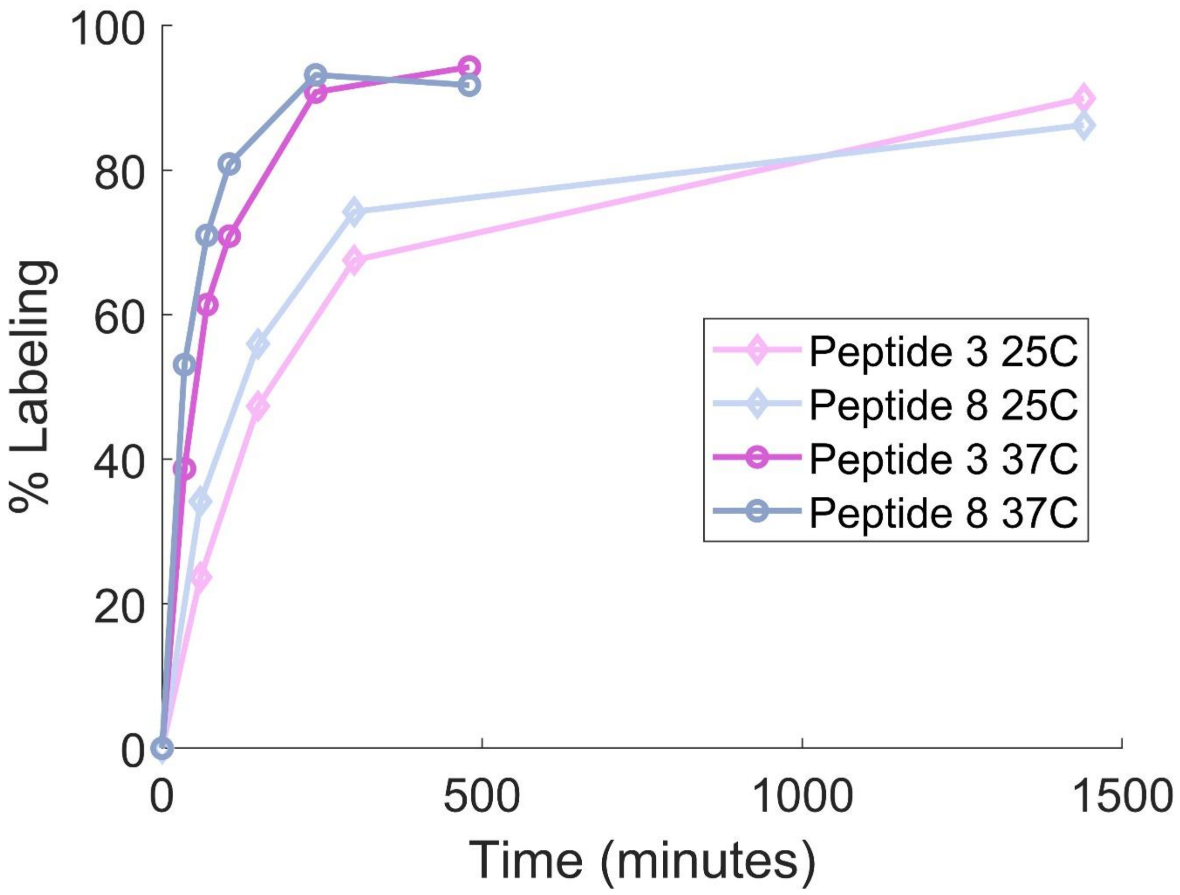

Supplementary Figure 2. Peptide labeling at various temperatures.

Peptides **3** and **8** (5 µM) were incubated with 14-3-3σ (2 µM) at either 25°C or 37°C and the degree of labeling was measured using LC/MS.

a

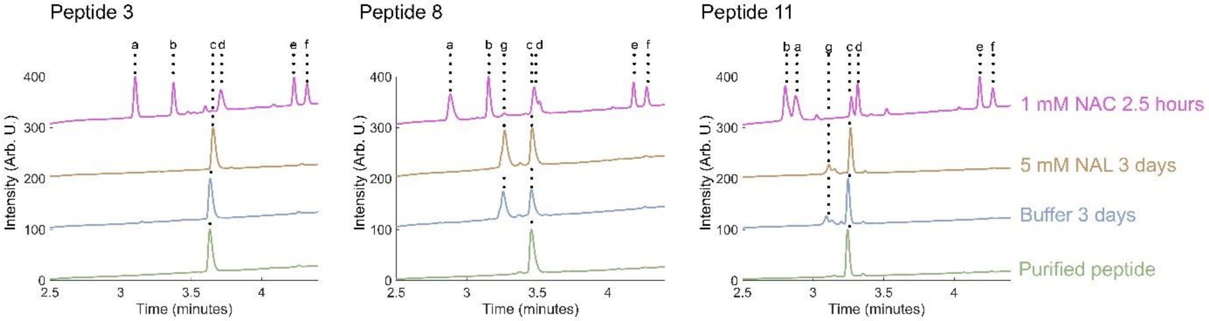

b

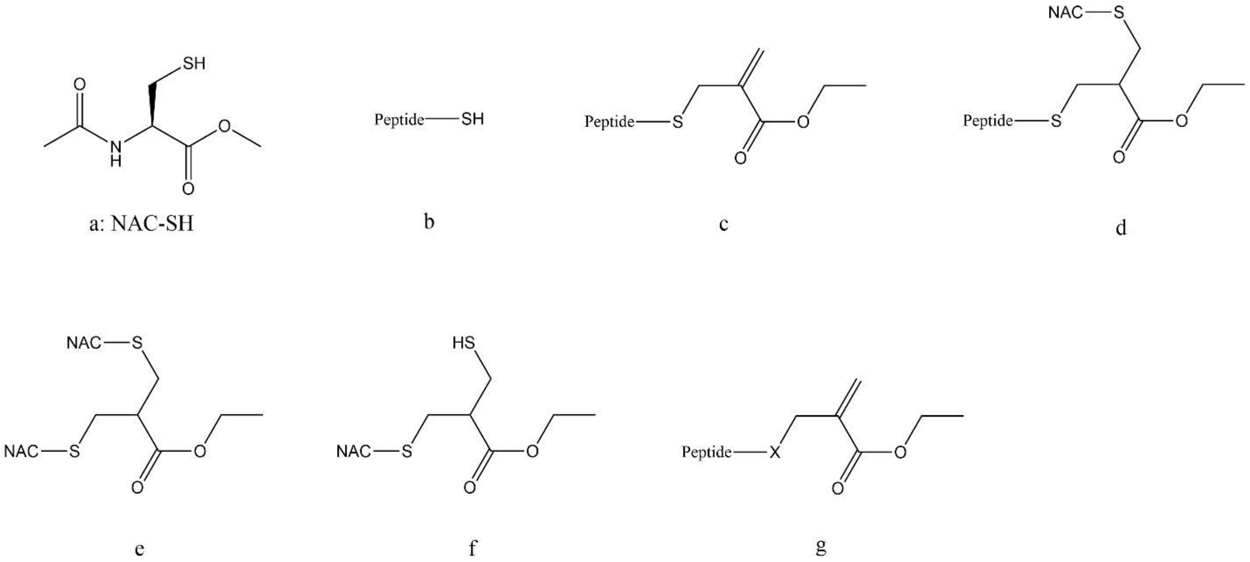

Supplementary Figure 3. Peptide stability in buffer and in the presence of lysine and cysteine.
a) Peptides **3**, **8** and **11** were incubated with 1 mM N-acetyl cysteine methyl ester (NAC, magenta) for 2.5 hours, 5 mM N-acetyl-lysine methylester (NAL, gold) for 3 days, and buffer only (HEPES 25 mM pH = 7.5, 100 mM NaCl, 10 mM $MgCl_2$) for 3 days, and the products were analyzed using LC/MS. The products were identified as shown in panel **b.** Product **g** is an isomerization product of peptides **8** and **11**, possibly due to migration of the methacrylate to a different position in the peptide, as it shows the same molecular mass, but different retention times to product **c**.

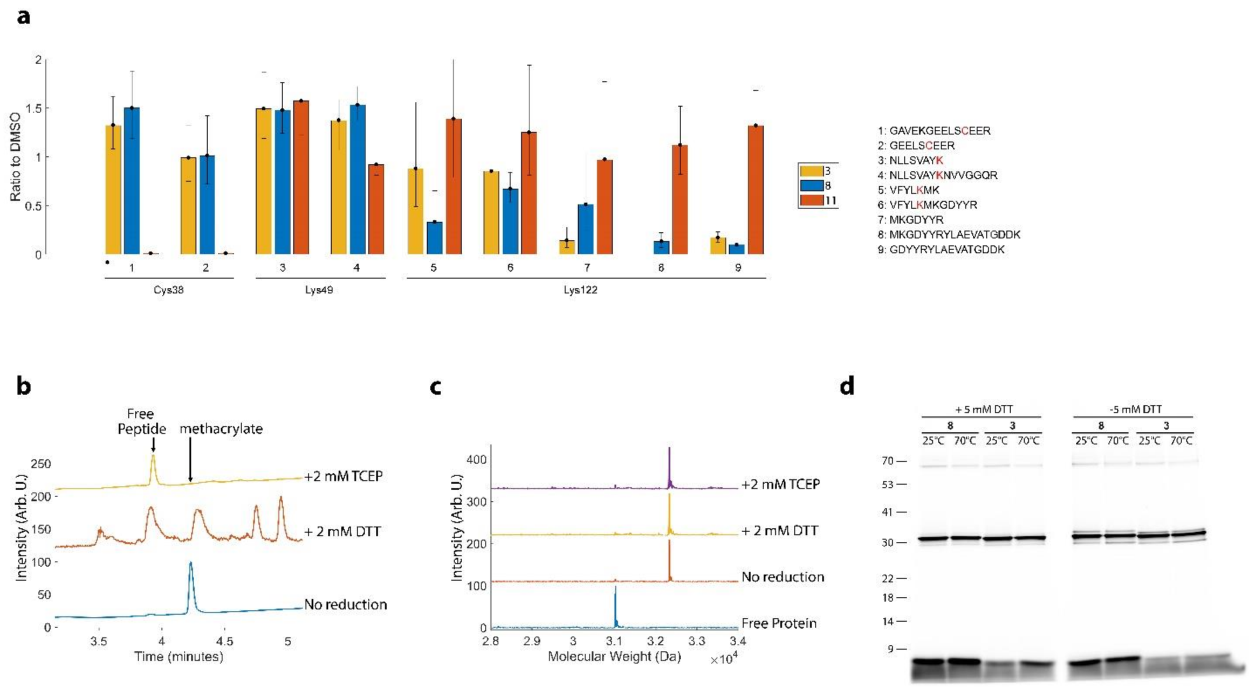

Supplementary Figure 4. Methacrylate peptides label different 14-3-3σ residues.
a) Following incubation with the peptides, samples of 14-3-3σ were trypsinized and tryptic peptides containing or following potential target residues were quantified using parallel reaction monitoring. 95% confidence intervals for the integrated signal for the peptides were calculated from n = 3 runs. The ratio to DMSO was calculated and the confidence interval for the ratio was calculated by standard error propagation. **b)** Peptide **8** was incubated with 2 mM of either TCEP or DTT for 130 minutes at room temperature and the products were characterized using LC/MS. **c)** 14-3-3σ was incubated with peptide **8** until fully labeled, followed by incubation with TCEP or DTT for 130 minutes at room temperature and analysis by LC/MS. **d)** 14-3-3σ was incubated with BODIPY-modified peptides until fully labeled. The samples were then denatured using Lithium Dodecyl Sulfate buffer in different conditions (with or without heating and with or without DTT) and analyzed by SDS-PAGE.

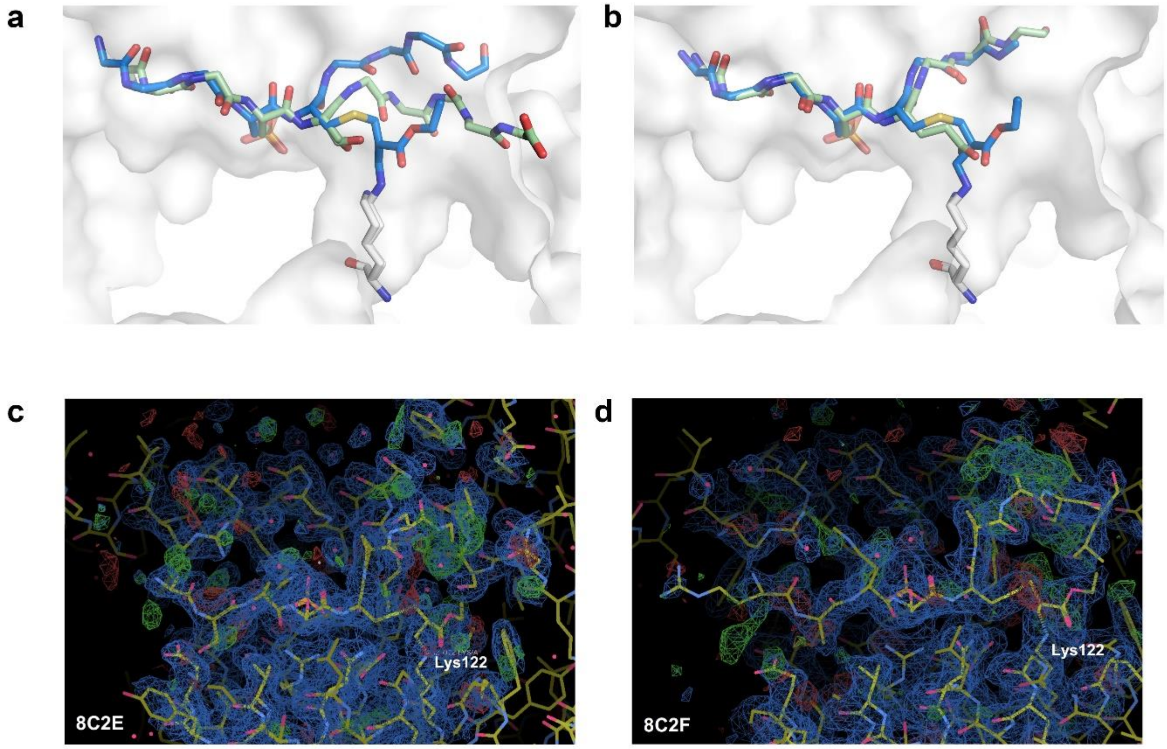

Supplementary Figure 5. Comparison of structures of covalent complexes and noncovalent complexes.

a) Comparison of the structure of the covalent complex between peptide **8** and 14-3-3σ (blue) with the noncovalent complex with the parent peptide (green). **b)** Comparison of the structure of the covalent complex between peptide **3** and 14-3-3σ (blue) with the noncovalent complex with the parent peptide (green). **c)** Portion of the electron density maps for peptide **3.** Displayed are 2Fo-Fc (blue mesh, contoured at 1.0 σ) and Fo-Fc maps (green and red meshes, contoured at 2.5 σ). **d)** Portion of the electron density maps for peptide **8.** Displayed are 2Fo-Fc (blue mesh, contoured at 1.0 σ) and Fo-Fc maps (green and red meshes, contoured at 2.5 σ).

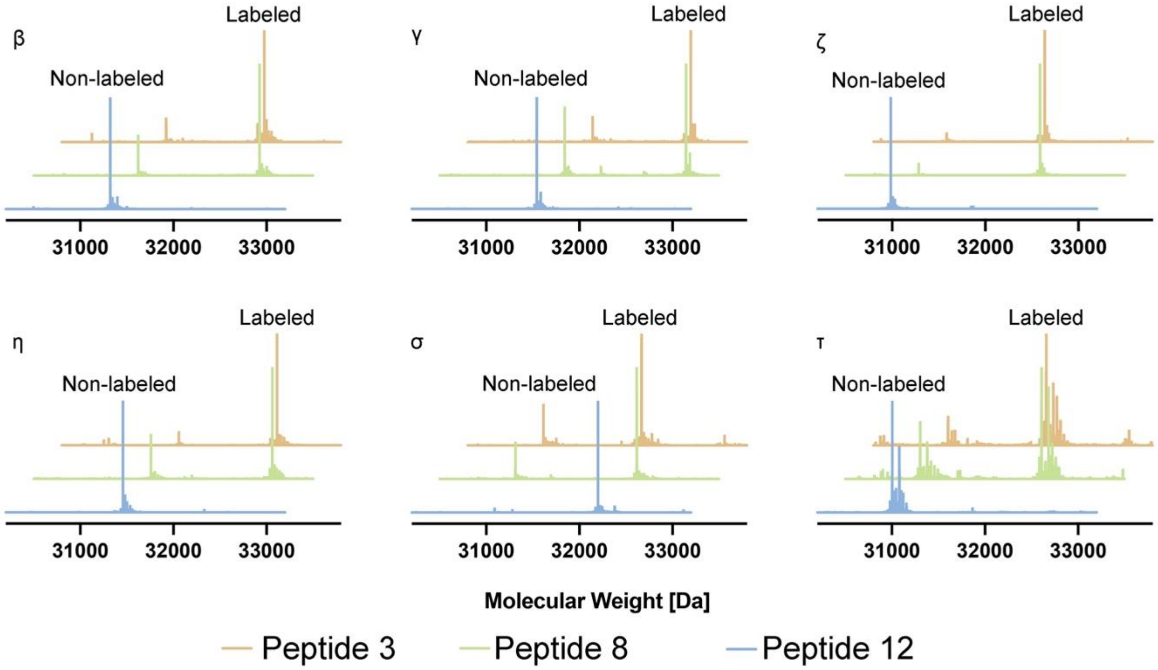

Supplementary Figure 6: Isoform selectivity of 14-3-3 covalent binding peptides

Binding of electrophilic peptides to 14-3-3 isoforms. Peptides were incubated with the various indicated isoforms overnight at room temperature, diluted and analyzed using intact protein LC/MS.

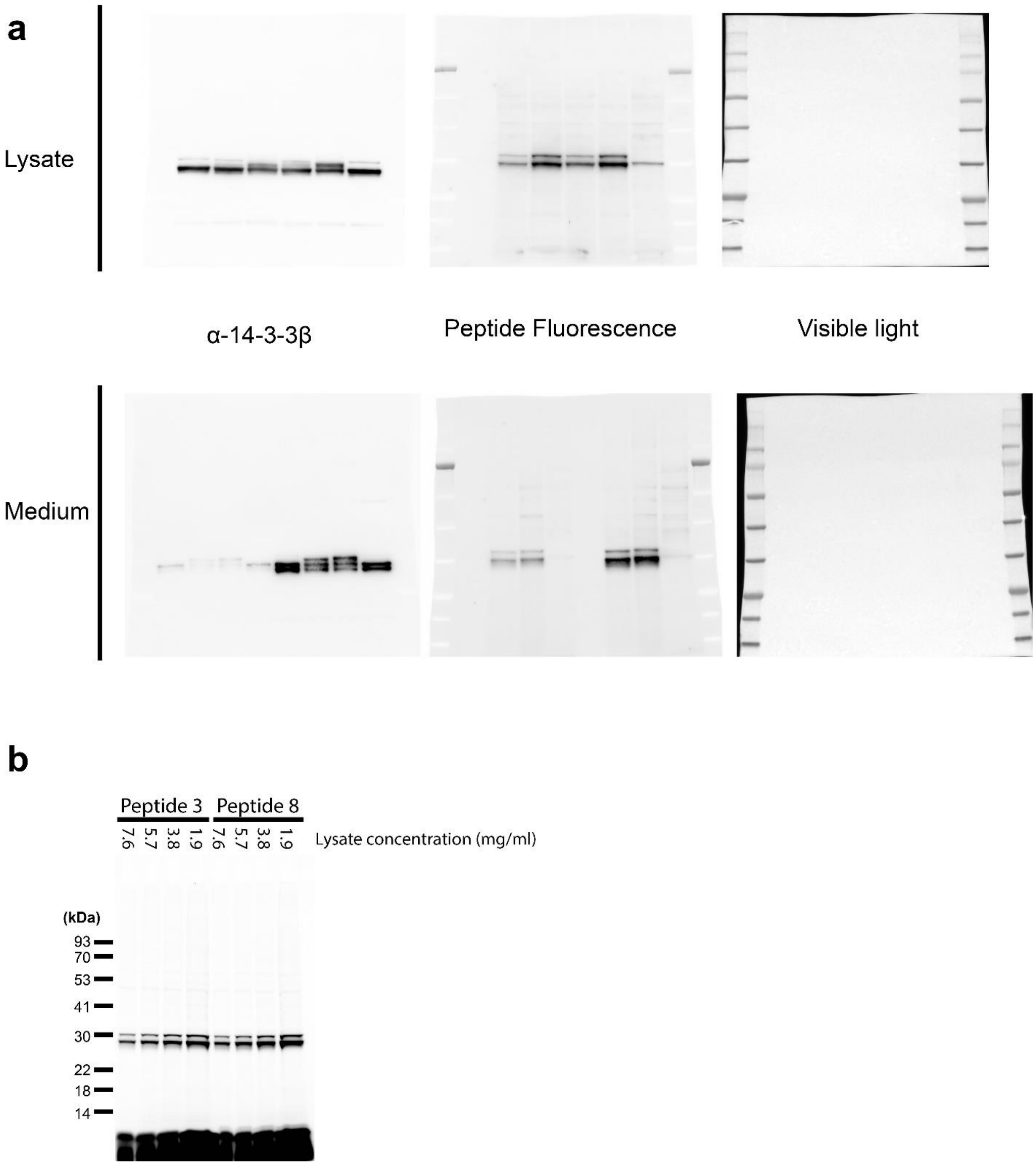

Supplementary Figure 7. Peptide binding selectivity in cell lysates and media.
a) Raw images for the western blot experiment depicted in figure 5. Experiments was repeated 3 times. Top row: images for experiment done on A549 lysates. Bottom row: Images from extracellular medium. **b)** Direct gel imaging for experiment in which 1 µM peptide was incubated with A549 lysates at the indicated concentrations for 22 hours at 25°C. The same amount of lysate was loaded in each lane (28.5 µg). The intense fluorescence at the bottom comes from unbound peptide.

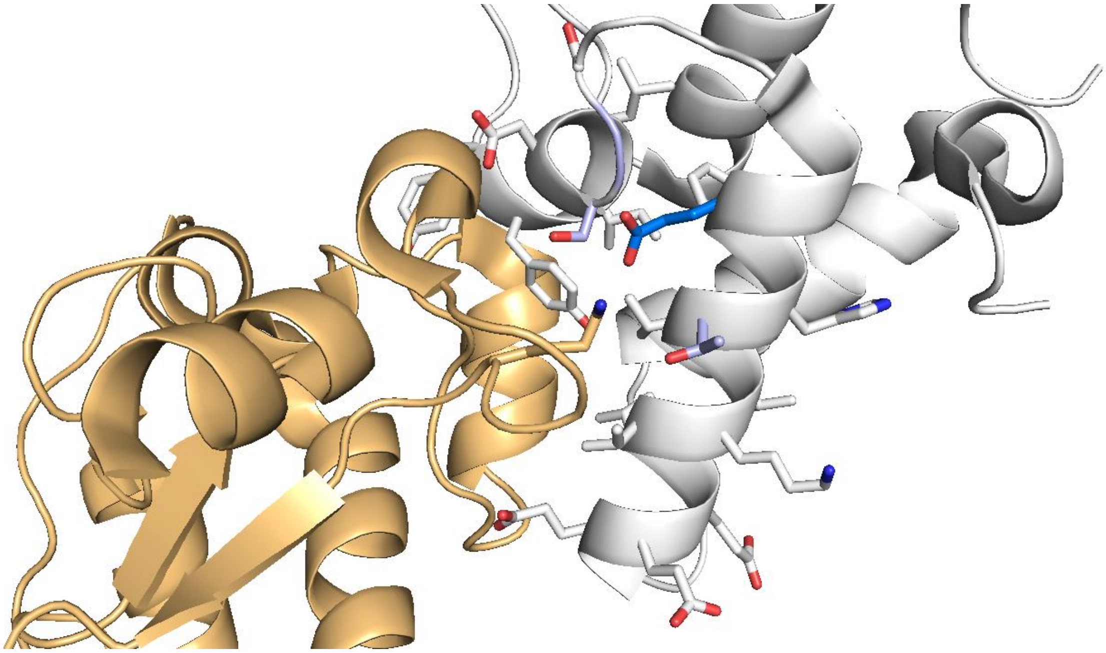

Supplementary Figure 8. Structure of the Im9-E9 complex (Im9: white; E9: gold).
Im9 residues mutated to cysteine and modeled as methacrylates are shown as white sticks. Residues that were mutated and gave interface RMSD below 1 Å are depicted in light blue. The selected residue (Glu41) is shown in blue, while the target residue in E9 (Lys122) is shown in gold.

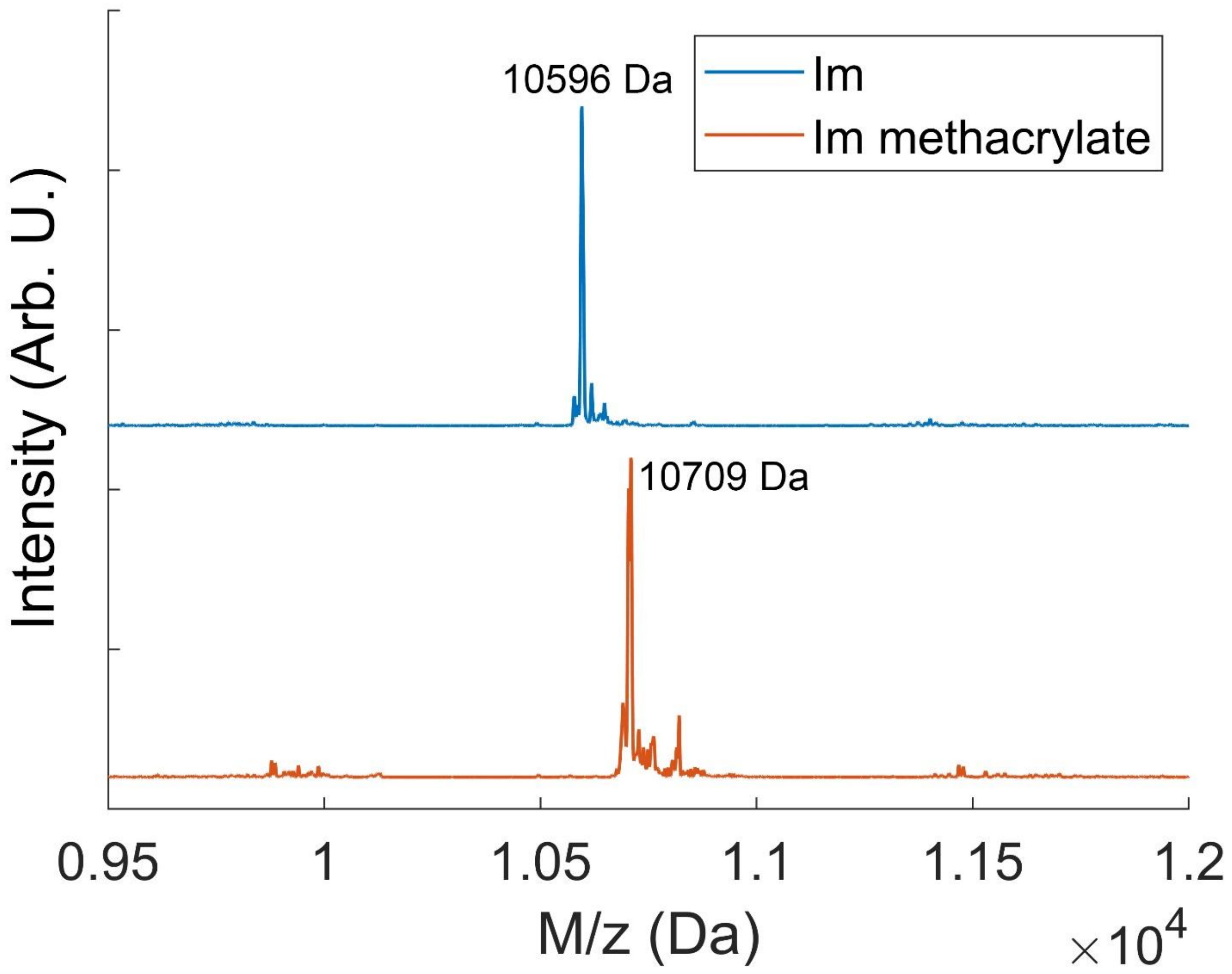

Supplementary Figure 9. Deconvoluted mass spectra of Im C23A/E41C mutant (Top) and the methacrylate-modified protein (bottom).

a

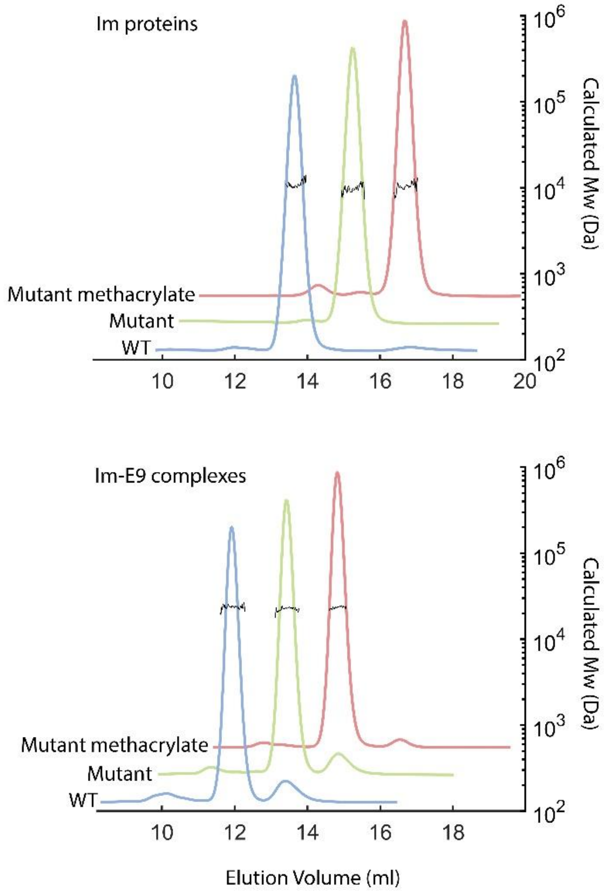

b

Protein	Mw from elution volume (kDa)	Mw from MALS (kDa)
Im	11.5 ± 0.8	11.0 ± 0.2
Im-mut	9.7 ± 1.3	9.92 ± 0.17
Im-mut methacrylate	10.3 ± 0.7	10.6 ± 0.2
Im-E9 complex	24.0 ± 1.0	23.7 ± 0.2
Im-mut-E9 complex	22.6 ± 0.7	22.8 ± 0.2
Covalent complex	23.4 ± 0.7	23.6 ± 0.1

Supplementary Figure 10. SEC-MALS evaluation of Im9/E9 complexes.
a) SEC-MALS measurements of Im proteins alone (top) and in complex with E9 (bottom), measured in Phosphate Buffered Saline, pH = 7.4. The colored plots show the normalized UV absorbance, and the black plots indicate the measured molecular weight from MALS. **b)** Calculated molecular weights of the constructs from both elution volume and MALS data.

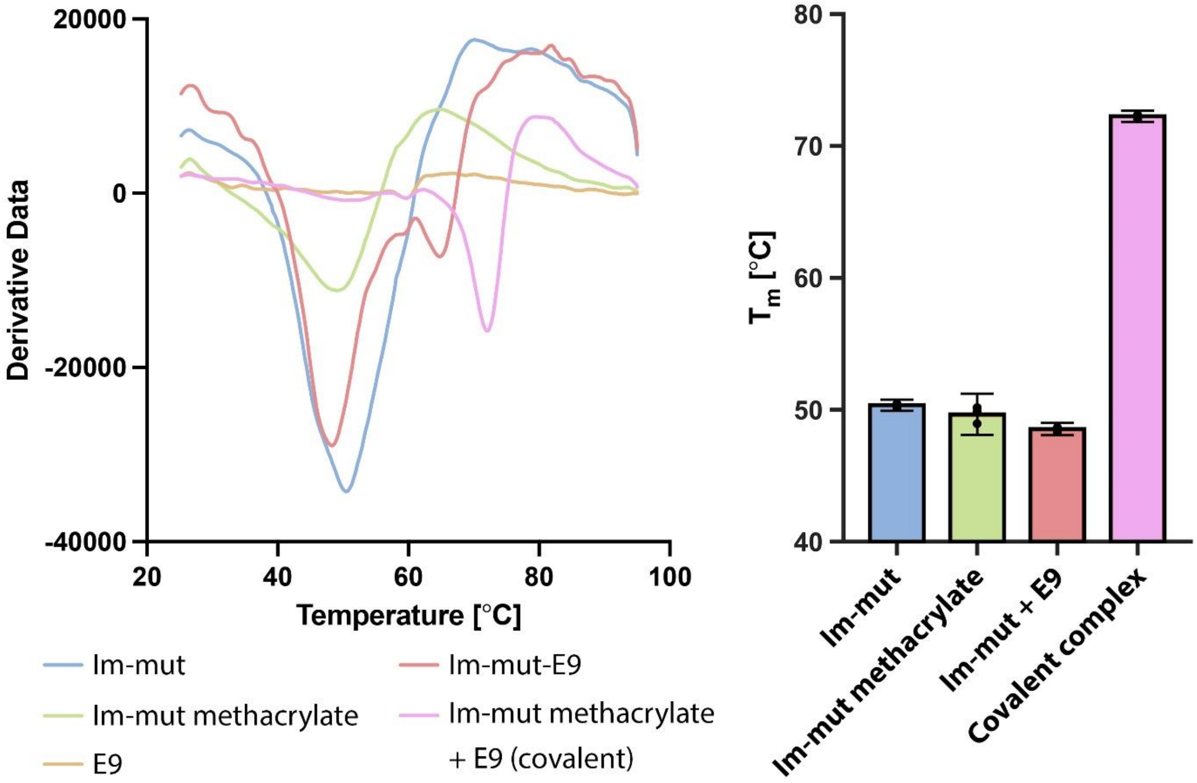

Supplementary Figure 11. Covalent complex formation significantly stabilizes complex thermal stability. Left: Average derivative data from 3 samples is shown for each condition. Right: measured melting temperature (calculated from maximum derivative) for different constructs. Error bars represent 95% confidence interval calculated using students T test (n=3 samples). Melting temperatures could not be calculated for E9 constructs alone which displayed no clear melting curve.

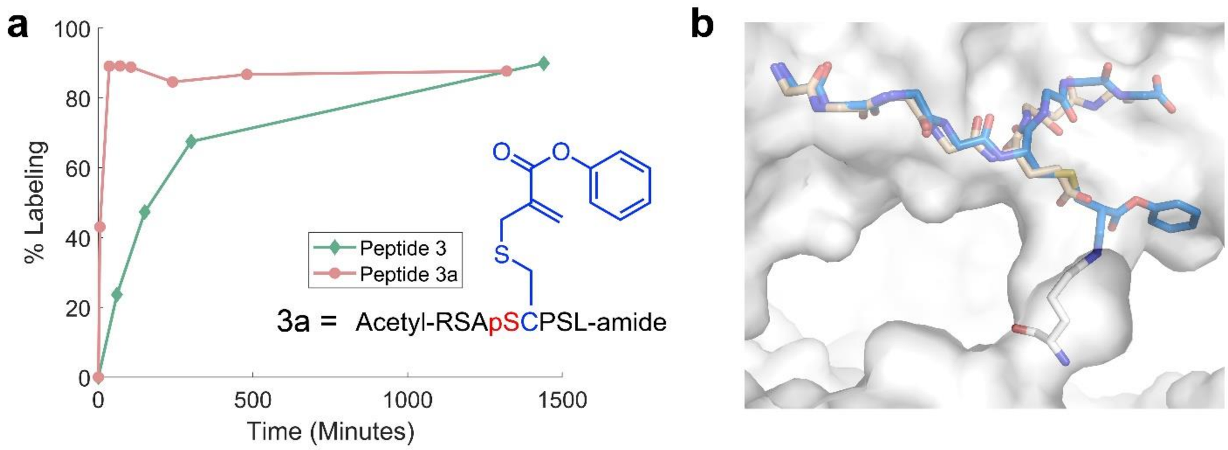

Supplementary Figure 12. Alternative electrophiles can tune the reaction kinetics. a) Labeling kinetics of 14-3-3σ (2 μM) by peptide **3** and phenyl ester derivative peptide **3a** (25°C, 5 μM peptide). **b)** CovPepDock model of peptide **3a** bound to 14-3-3σ (blue) overlaid on the structure of parent noncovalent peptide (teal).

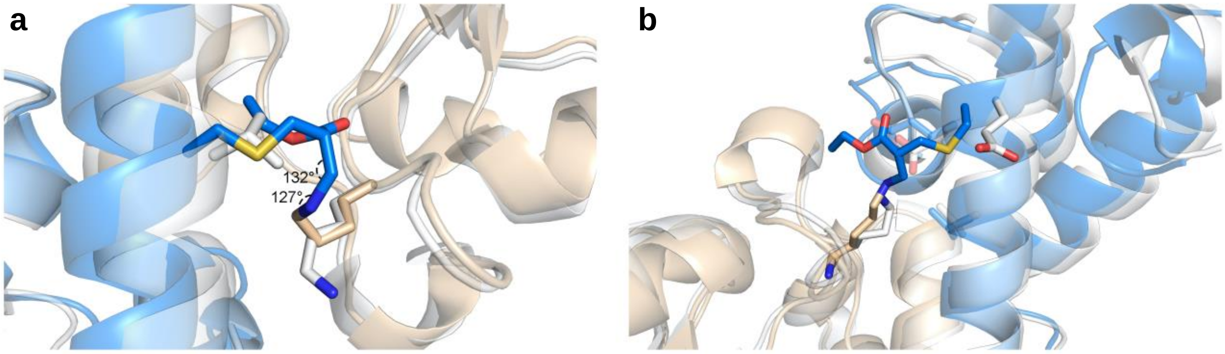

Supplementary Figure 13. Structural insights provided by CovPepDock for covalent protein reagents. a) Top-scoring model of the E9-Im9 complex when mutating Val34 of Im9 to the methacrylate warhead. While this position is located in close proximity to the target Lys97 (Cα-Cα distance = 7.5Å), none of the 10 top-scoring models had constraint score < 2, indicating a non-ideal covalent bond geometry. **B)** Docking model (RMSD = 0.758Å) of the E9-Im9 complex when mutating Glu42. While this position seems to be pointing away from the target Lys97, CovPepDock revealed a potential shift of the helix that enables the formation of the covalent bond.

Supplementary Tables

Supplementary Table 1. Modeling statistics for incorporation of an electrophilic residue to 14-3-3 binding peptides.

PDB	14-3-3 Target Lysine	Peptide Position	Best interface backbone RMSD (Å)[a]
3MHR	49	124	-
		125	2.197
		126	1.276
		127	0.574
		128	0.978
		129	0.498
		130	0.608
		131	0.609
		132	1.046
	122	126	3.279
		127	0.619
		128	0.647
		129	2.363
		130	0.843
		131	0.948
		132	0.444
		133	2.087
3IQU	122	257	-
		258	6.085
		259	0.969
		260	0.455
3P1N	122	372	4.810
		373	1.642
		374	0.438
4IEA	122	620	-
		621	1.279
		622	0.529
		623	1.920
		624	0.747
		625	1.068
4QLI	122	175	-
		176	3.688
		177	1.428
		178	0.640
		179	0.770
		180	1.411
7NFW	122	592	-
		593	3.979
		594	0.654
		595	0.317

[a] Best RMSD among the top-10 scoring models that also have a constraint score < 2. If no RMSD is listed it means that no models with constraint score < 2 were generated.

Supplementary Table 2. Data collection and refinement statistics for 14-3-3σ bound to 3 (PDB: 8C2E) and 8 (8C2F)

**PDB**	**8C2E**	**8C2F**
Protein	14-3-3σDc	14-3-3σDc
Peptide	**3**	**8**
Beam	DESY p11	DESY p11
Data Collection		
Wavelength (Å)	1.03322	1.03322
Space Group	C 2 2 21	C 2 2 21
Cell Dimensions		
a, b, c (Å)	82.5, 111.7, 63.1	82.8, 112.2, 63.0
α, β, γ (°)	90, 90, 90	90, 90, 90
Resolution (Å)	55.92 – 2.20 (2.27-2.20)	56.14-2.30 (2.38-2.30)
I / σ(I)	12.1 (6.6)	8.2 (5.0)
Completeness (%)	99.7 (99.4)	99.9 (100)
Redundancy	12.8 (10.4)	13.1 (13.1)
$CC_{1/2}$	0.991 (0.895)	0.980 (0.720)
Refinement		
Number of Reflections	15133	13362
R_{work} / R_{free}	0.175/0.221	0.201/0.267
Number of Atoms		
Protein	3516	3515
Ligand/Ion	105/4	150/4
Water	110	59
B-factors		
Protein	18.98	17.45
Ligand/ion	18.01/43.35	14.18/36.09
Water	23.72	18.83
R.M.S deviations		
Bond Lengths (Å)	0.0091	0.0072
Bond Angles (°)	1.515	1.397
Ramachandran		
Favored (%)	98.69	97.39
Outliers (%)	0.00	0.87
