# Supplementary material for: A simple method for developing lysine targeted covalent protein reagents
Source: Nat Commun. 2023 Dec 1;14:7933. doi: 10.1038/s41467-023-42632-5 (PMC10692228; doi:10.1038/s41467-023-42632-5)
Supplement: Supplementary file 3 — Reporting Summary [file 41467_2023_42632_MOESM3_ESM.pdf]

nature portfolio

Corresponding author(s): Nir London

Last updated by author(s): Sep 26, 2023

Reporting Summary

Nature Portfolio wishes to improve the reproducibility of the work that we publish. This form provides structure for consistency and transparency in reporting. For further information on Nature Portfolio policies, see our Editorial Policies and the Editorial Policy Checklist.

Statistics

For all statistical analyses, confirm that the following items are present in the figure legend, table legend, main text, or Methods section.

n/a	Confirmed	
☐	☒	The exact sample size (*n*) for each experimental group/condition, given as a discrete number and unit of measurement
☐	☒	A statement on whether measurements were taken from distinct samples or whether the same sample was measured repeatedly
☐	☒	The statistical test(s) used AND whether they are one- or two-sided<br>*Only common tests should be described solely by name; describe more complex techniques in the Methods section.*
☒	☐	A description of all covariates tested
☐	☒	A description of any assumptions or corrections, such as tests of normality and adjustment for multiple comparisons
☐	☒	A full description of the statistical parameters including central tendency (e.g. means) or other basic estimates (e.g. regression coefficient) AND variation (e.g. standard deviation) or associated estimates of uncertainty (e.g. confidence intervals)
☐	☒	For null hypothesis testing, the test statistic (e.g. *F*, *t*, *r*) with confidence intervals, effect sizes, degrees of freedom and *P* value noted<br>*Give P values as exact values whenever suitable.*
☒	☐	For Bayesian analysis, information on the choice of priors and Markov chain Monte Carlo settings
☒	☐	For hierarchical and complex designs, identification of the appropriate level for tests and full reporting of outcomes
☒	☐	Estimates of effect sizes (e.g. Cohen's *d*, Pearson's *r*), indicating how they were calculated

Our web collection on statistics for biologists contains articles on many of the points above.

Software and code

Policy information about availability of computer code

Data collection	Intact protein LCMS was measured using MassLynx version 4.2. LCMSMS data was collected using XCalibur version 4.4.16.14
Data analysis	Intact protein LCMS data was analyzed using MassLynx MaxEnt feature. Analysis of LCMSMS data was performed using MaxQuant 1.6.3.4, Skyline 22.2.0.351, and fragpipe 19.1. Crystallography data was analyzed using the following software: XIA2/DIALS, AIMLESS, MOLREP, RefmacS, Coot and AceDRG

For manuscripts utilizing custom algorithms or software that are central to the research but not yet described in published literature, software must be made available to editors and reviewers. We strongly encourage code deposition in a community repository (e.g. GitHub). See the Nature Portfolio guidelines for submitting code & software for further information.

Data

Policy information about availability of data

All manuscripts must include a data availability statement. This statement should provide the following information, where applicable:

- Accession codes, unique identifiers, or web links for publicly available datasets
- A description of any restrictions on data availability
- For clinical datasets or third party data, please ensure that the statement adheres to our policy

Crystal structures have been deposited to the protein data bank (PDB) using IDs 8C2E and 8C2F. Proteomics data was deposited to the ProteomeXchange Consortium via the PRIDE partner repository with the dataset identifiers PXD044257 and PXD044294. The rest of the data presented in this manuscript is given in the Source Data file.

Research involving human participants, their data, or biological material

Policy information about studies with human participants or human data. See also policy information about sex, gender (identity/presentation), and sexual orientation and race, ethnicity and racism.

Reporting on sex and gender	N/A
Reporting on race, ethnicity, or other socially relevant groupings	N/A
Population characteristics	N/A
Recruitment	N/A
Ethics oversight	N/A

Note that full information on the approval of the study protocol must also be provided in the manuscript.

Field-specific reporting

Please select the one below that is the best fit for your research. If you are not sure, read the appropriate sections before making your selection.

☒ Life sciences ☐ Behavioural & social sciences ☐ Ecological, evolutionary & environmental sciences

For a reference copy of the document with all sections, see nature.com/documents/nr-reporting-summary-flat.pdf

Life sciences study design

All studies must disclose on these points even when the disclosure is negative.

Sample size	Intact protein LCMS samples and western blot experiments were measured as individual samples and experiments were repeated 3 times to ensure reproducibility. For LC-MSMS experiments to determine labeling sites, three injections from each sample were performed. For Pull down proteomics experiments, four samples were prepared separately for each conditions. This was done because >200 proteins were identified, so data from at least 3 replicates was needed to calculate differences with reliable P-values. Quadruplicates were selected as a precaution in case some samples were damaged during the protocol, which incidentally did not happen.
Data exclusions	No Data was excluded.
Replication	Repetitions were performed within 2 weeks of each other.
Randomization	There was no need to perform any randomization in the experiments - proteomics pull down experiments were performed in several conditions, each one in quadruplicates.
Blinding	No blinding was performed because all experiments were performed on in vitro samples that were processed and analyzed in parallel,

Reporting for specific materials, systems and methods

We require information from authors about some types of materials, experimental systems and methods used in many studies. Here, indicate whether each material, system or method listed is relevant to your study. If you are not sure if a list item applies to your research, read the appropriate section before selecting a response.

Materials & experimental systems

n/a	Involved in the study	
☐	☒	Antibodies
☐	☒	Eukaryotic cell lines
☒	☐	Palaeontology and archaeology
☒	☐	Animals and other organisms
☒	☐	Clinical data
☒	☐	Dual use research of concern
☒	☐	Plants

Methods

n/a	Involved in the study	
☒	☐	ChIP-seq
☒	☐	Flow cytometry
☒	☐	MRI-based neuroimaging

Antibodies

Antibodies used

Anti 14-3-3 beta antibody (abcam ab 15260), anti-rabbit HRP (CST 7074S)

Validation

All our experiments showed a prominent band at the expected mass. Validation appears in the manufacturer's website was follows:
Ab15260: https://www.abcam.com/products/primary-antibodies/14-3-3-beta-antibody-ab15260.html
Single band appears at 28 kDa as expected in HeLa Cell lysate

CST 7074S: https://www.cellsignal.com/datasheet.jsp?productId=7074&images=1&size=A4
Validated with CST primary antibodies to react specifically with Rabbit antibodies

Eukaryotic cell lines

Policy information about cell lines and Sex and Gender in Research

Cell line source(s)

A549 cells were obtained from biological services of the Weizmann Institute

Authentication

No Authentication was performed

Mycoplasma contamination

Cells were tested for mycoplasma and confirmed negative

Commonly misidentified lines
(See ICLAC register)

None
