# Supplementary material for: A simple method for developing lysine targeted covalent protein reagents
Source: Nat Commun. 2023 Dec 1;14:7933. doi: 10.1038/s41467-023-42632-5 (PMC10692228; doi:10.1038/s41467-023-42632-5)

Peptide 1:


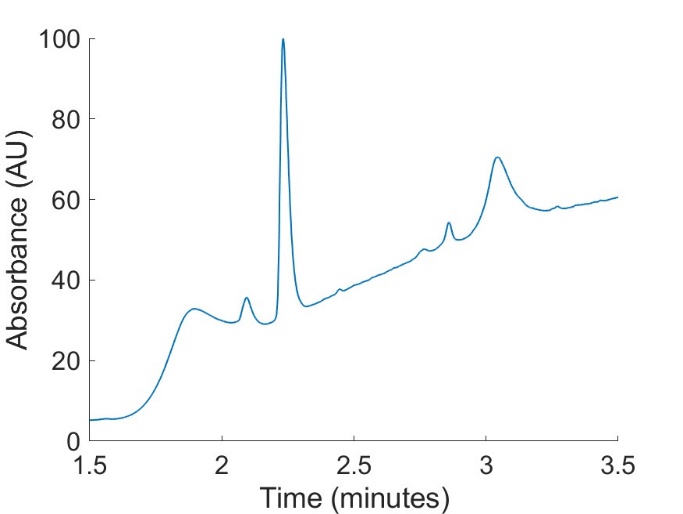

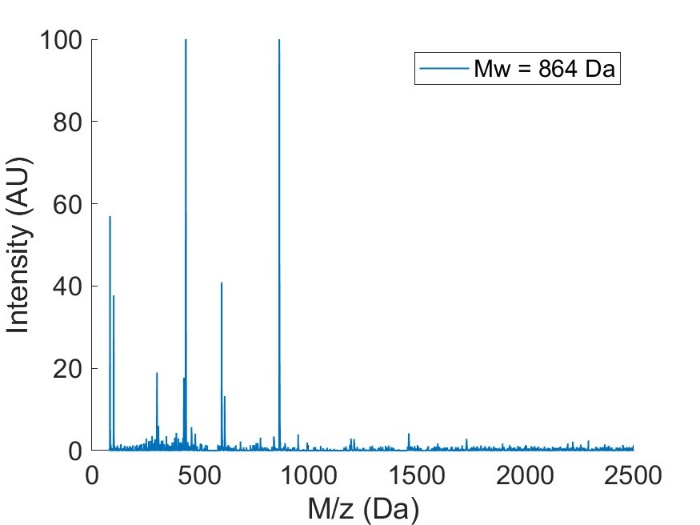


Peptide 2:


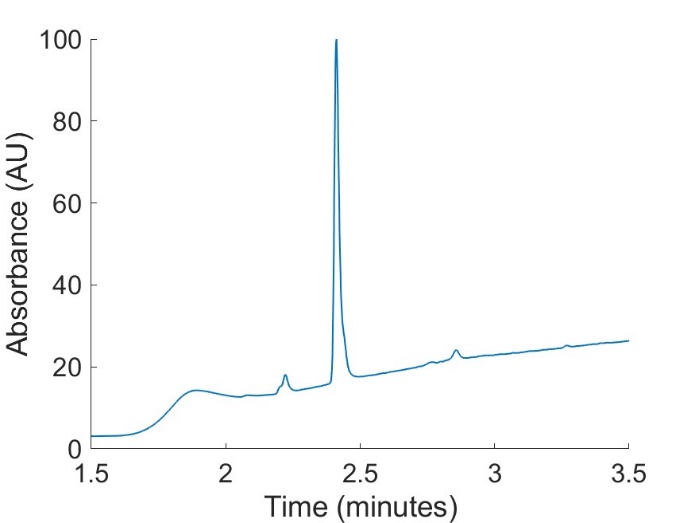

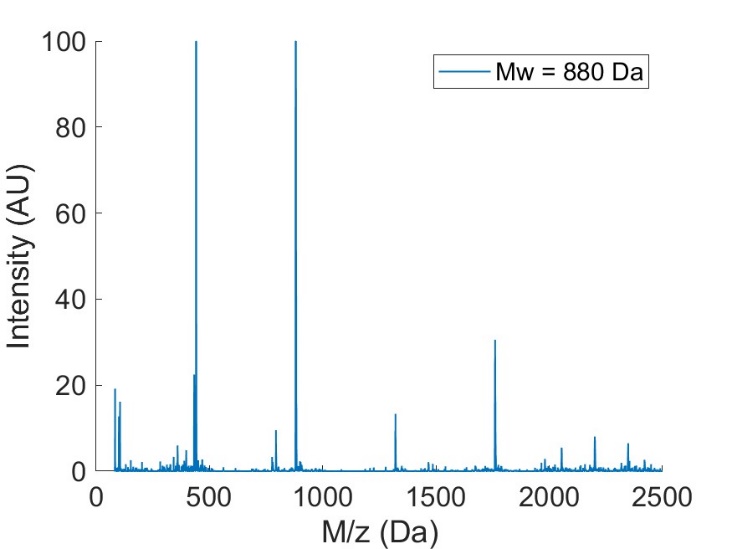


Peptide 3:


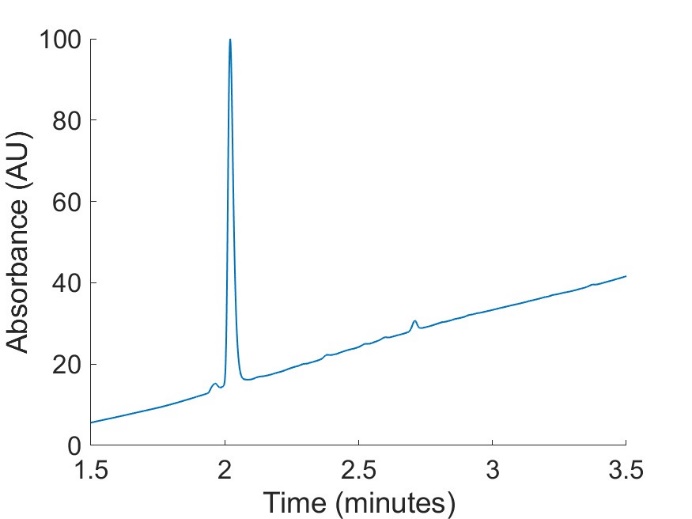

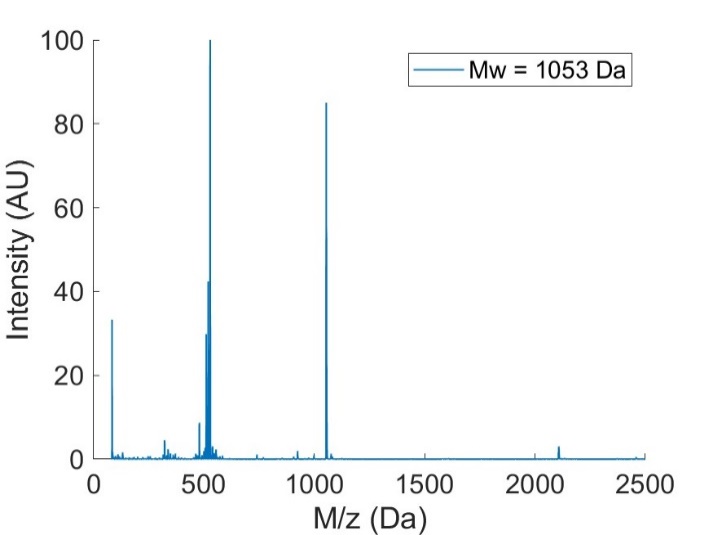


Peptide 4:


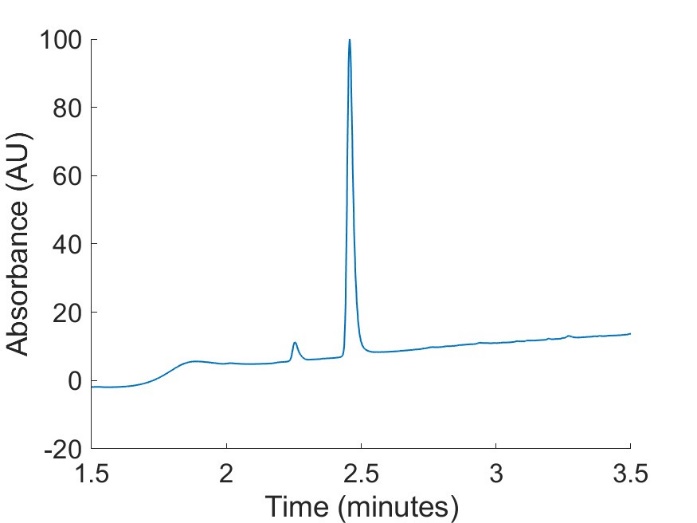

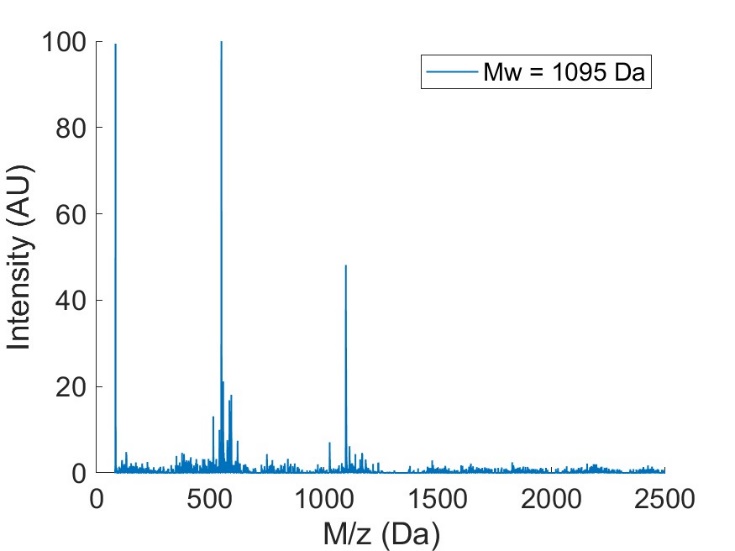


Peptide 5:


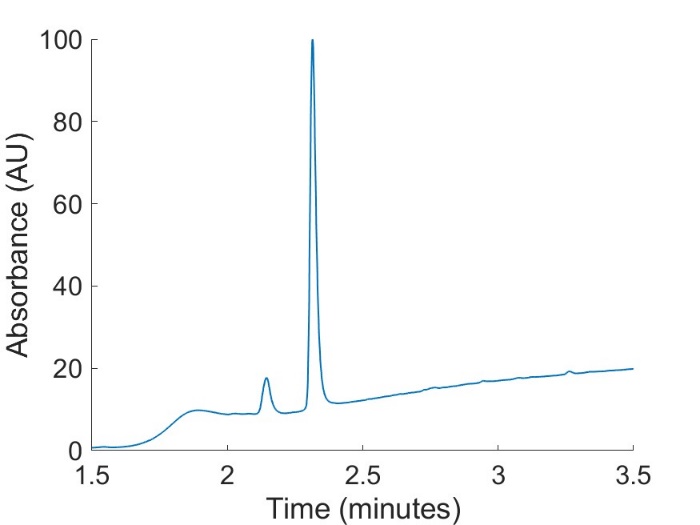

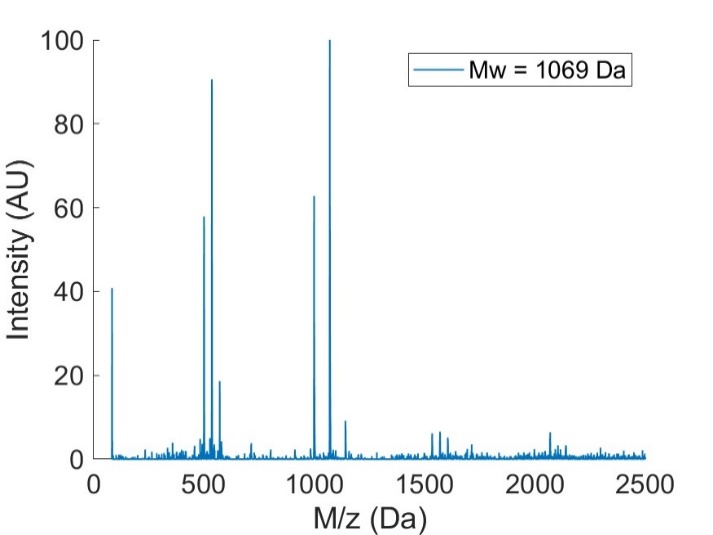


Peptide 6:


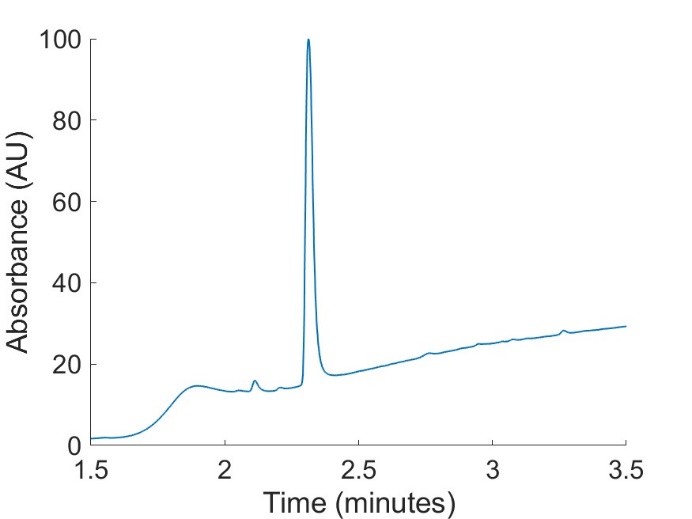

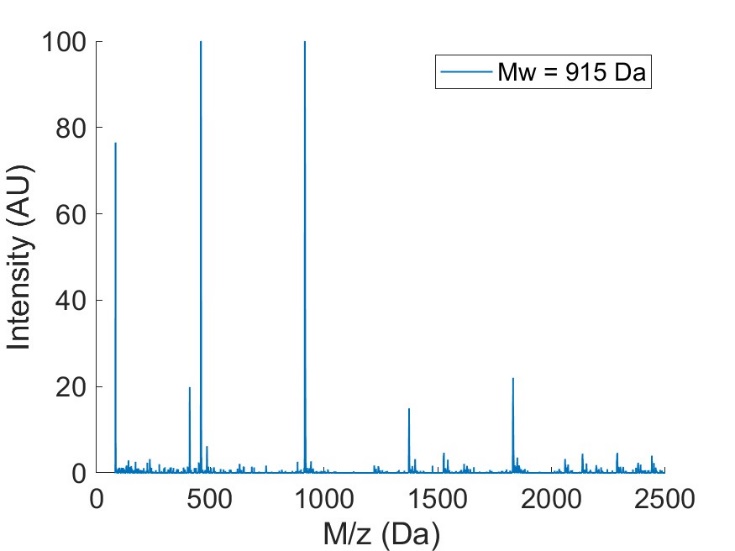


Peptide 7:


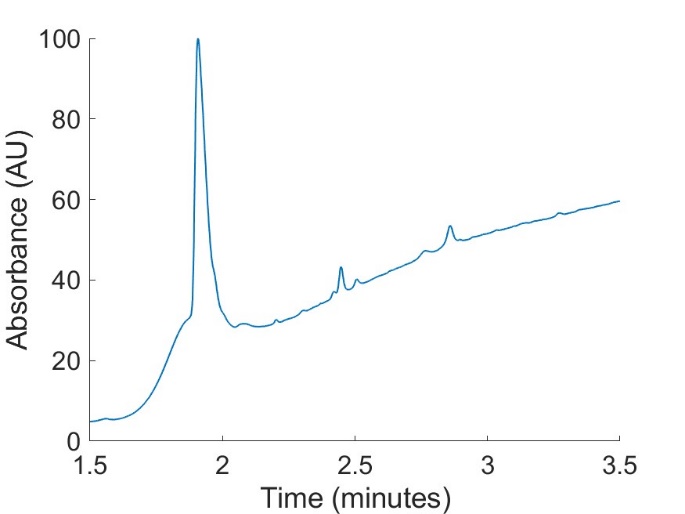

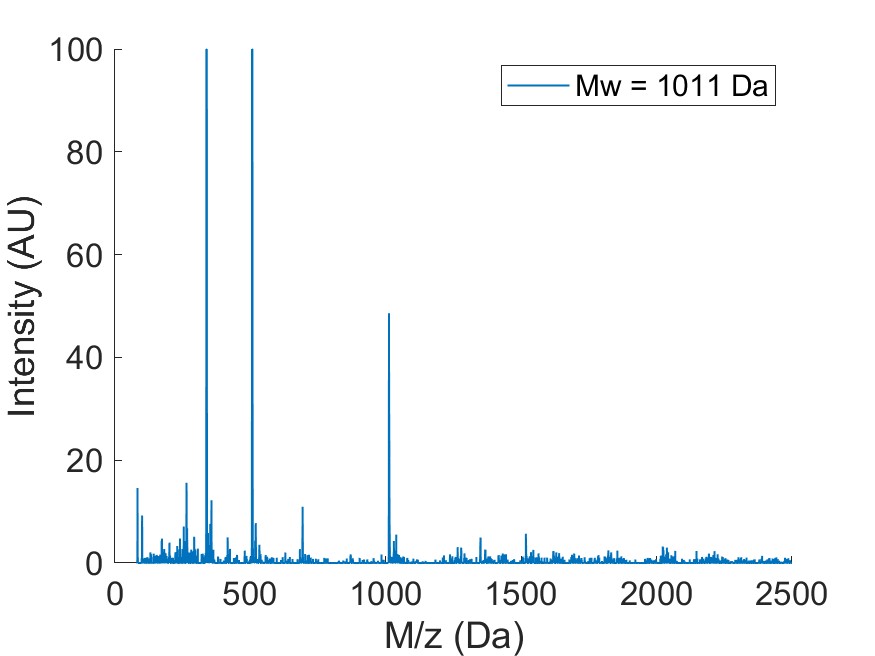


Peptide 8:


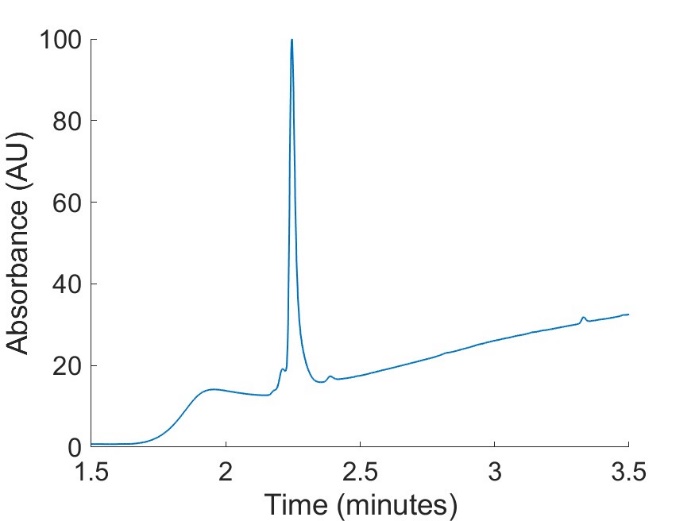

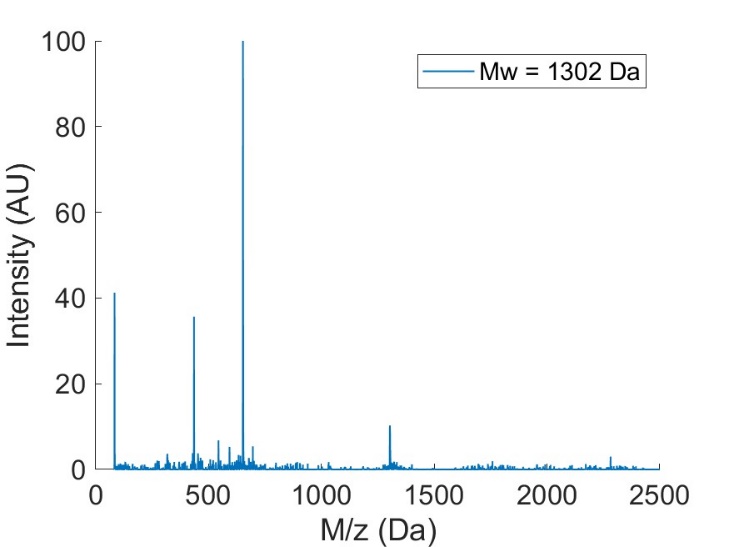


Peptide 9:


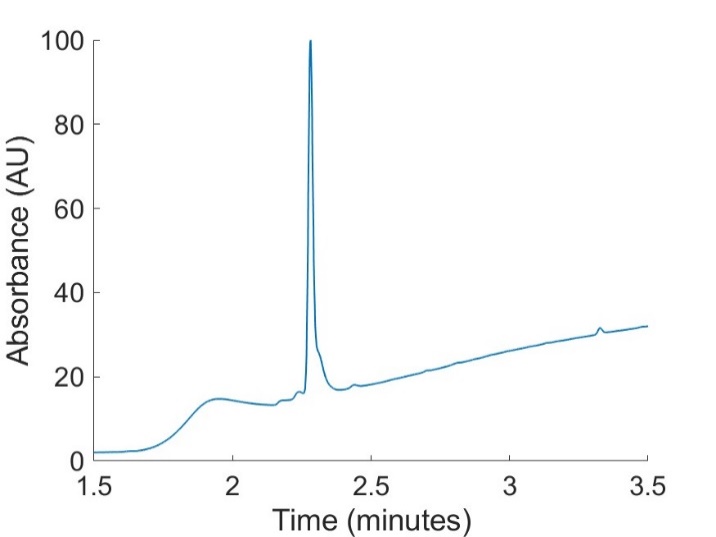

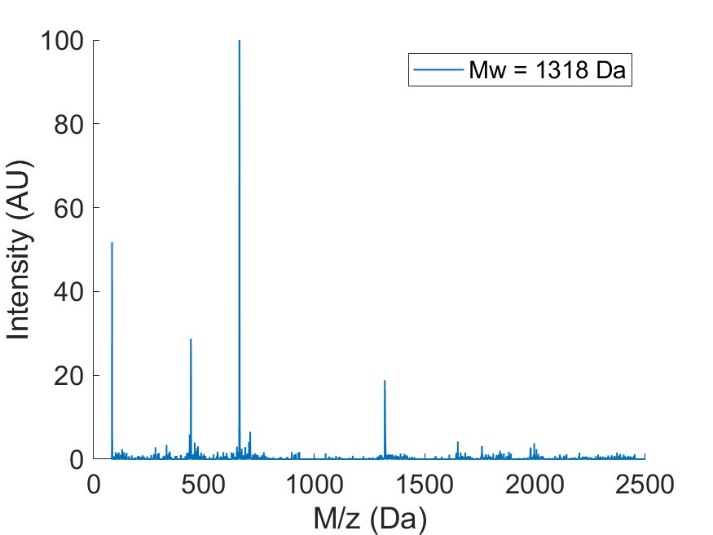


Peptide 10:


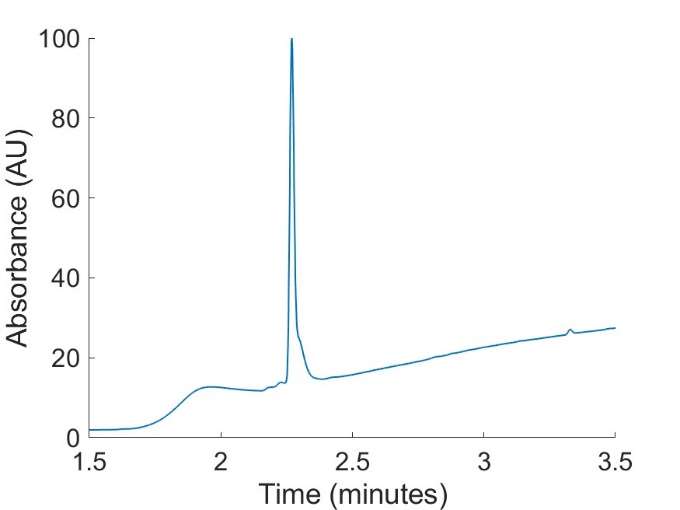

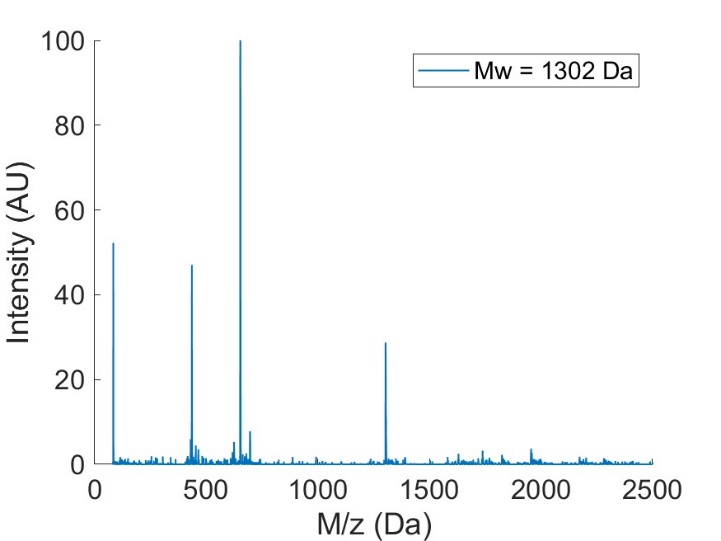


Peptide 11:


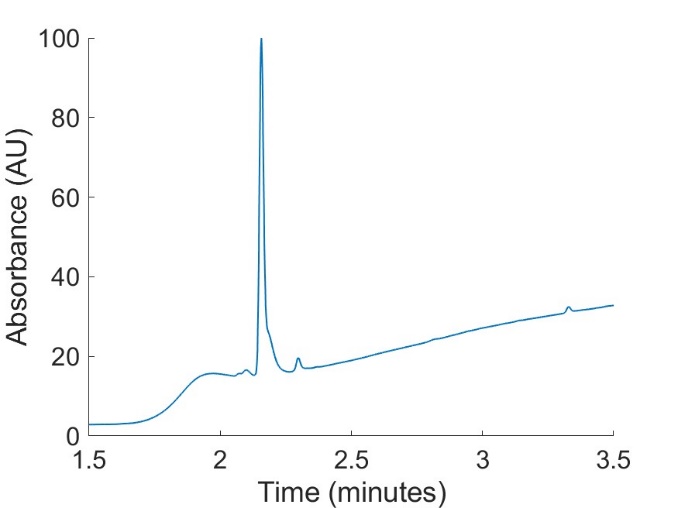

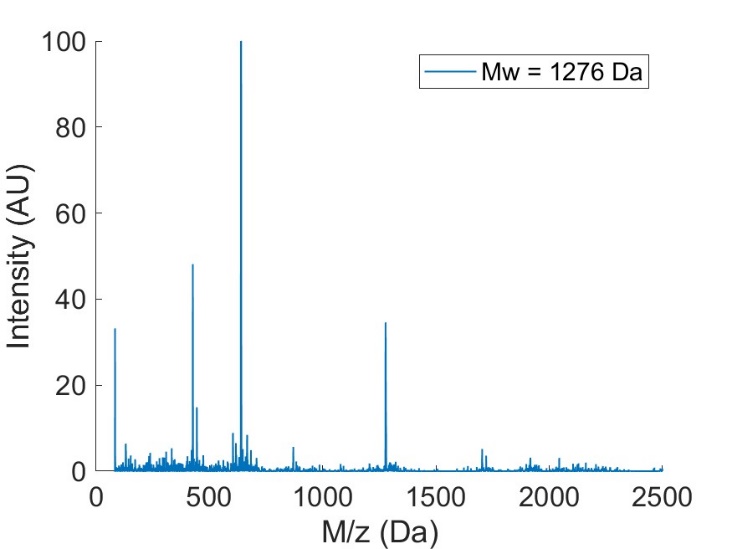


PropargylGly-peptide 3:


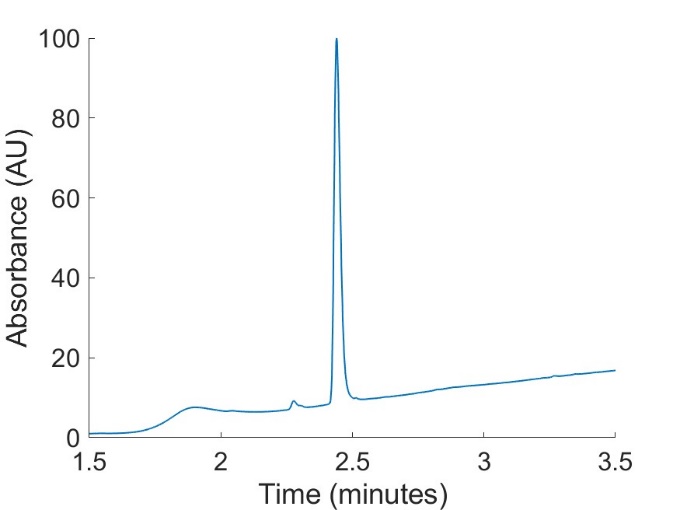

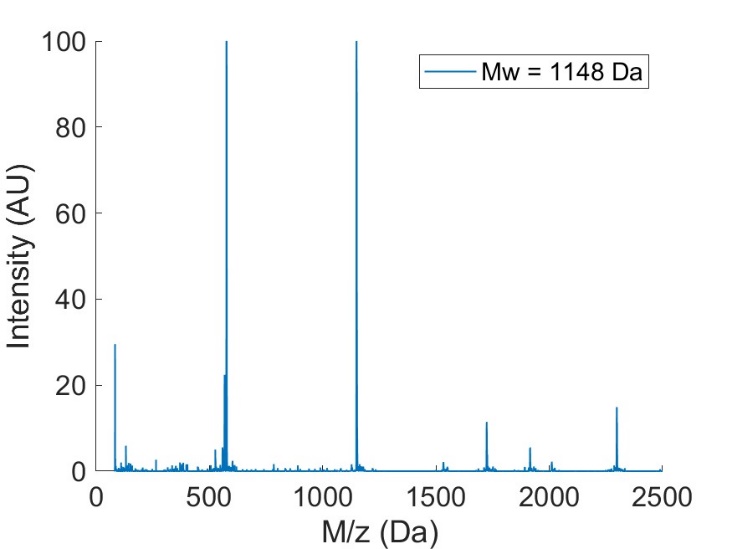


PropargylGly-peptide 8:


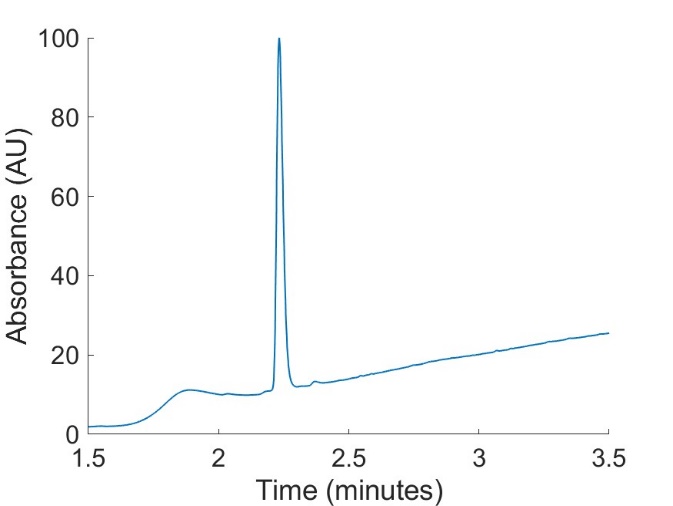

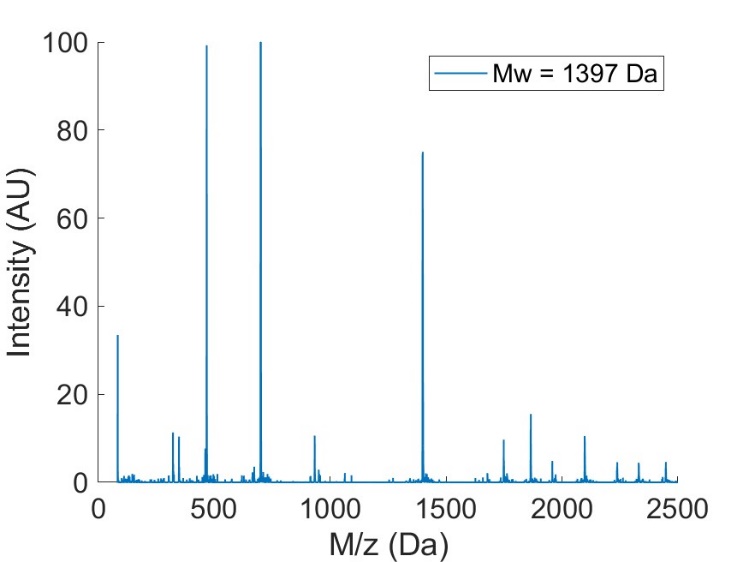


BDP-peptide 3:


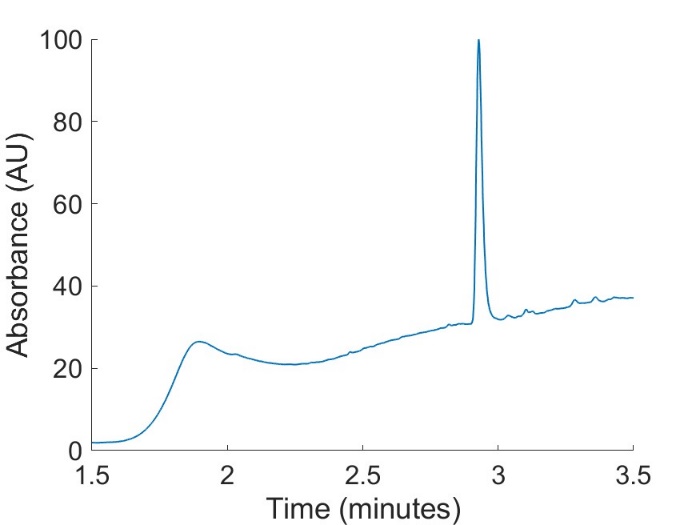

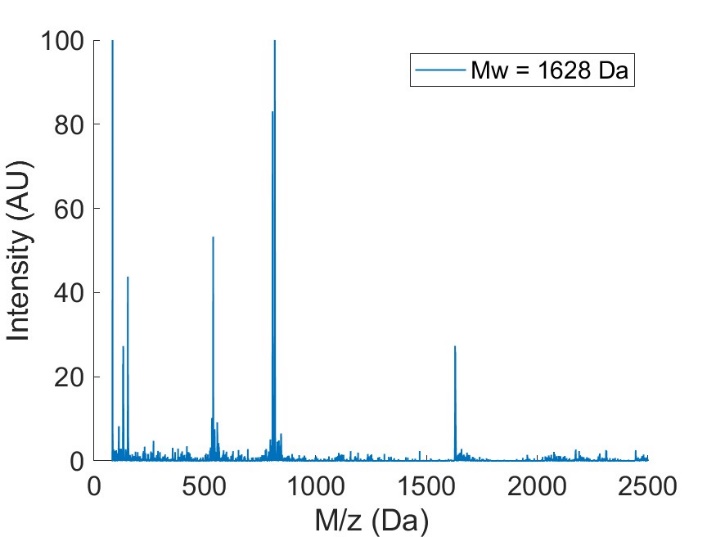


BDP-peptide 8:


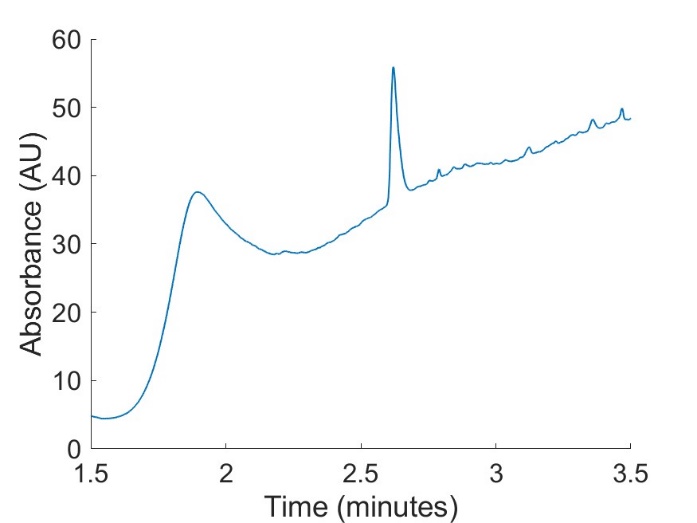

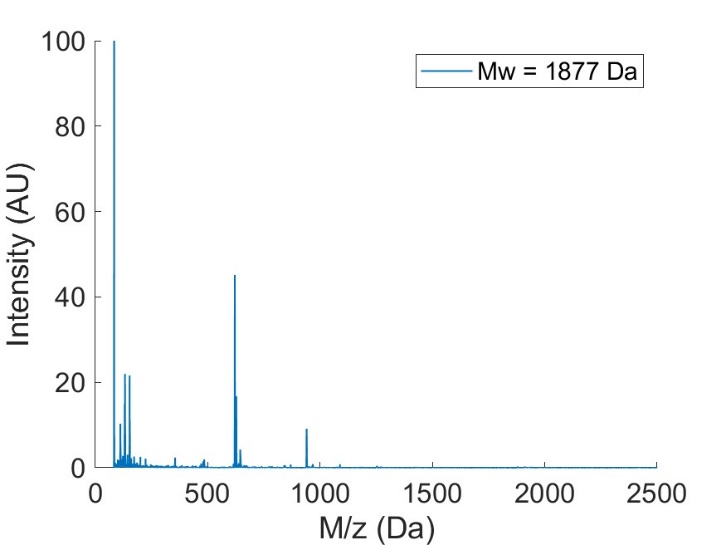


Biotin-Peptide 3:


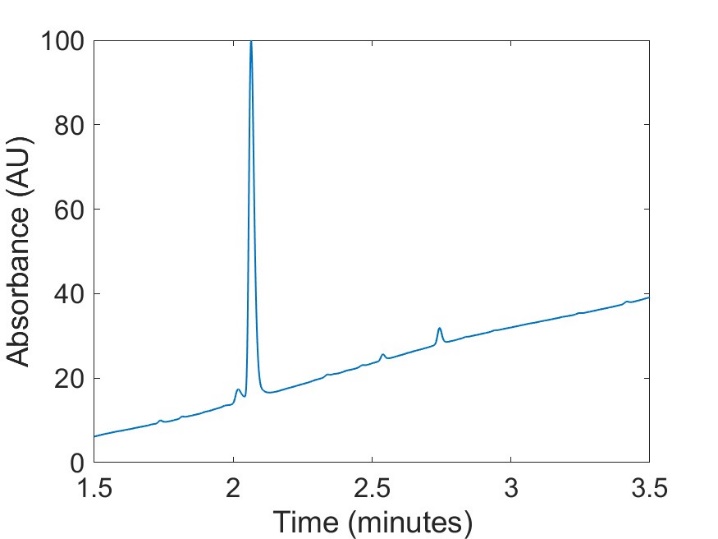

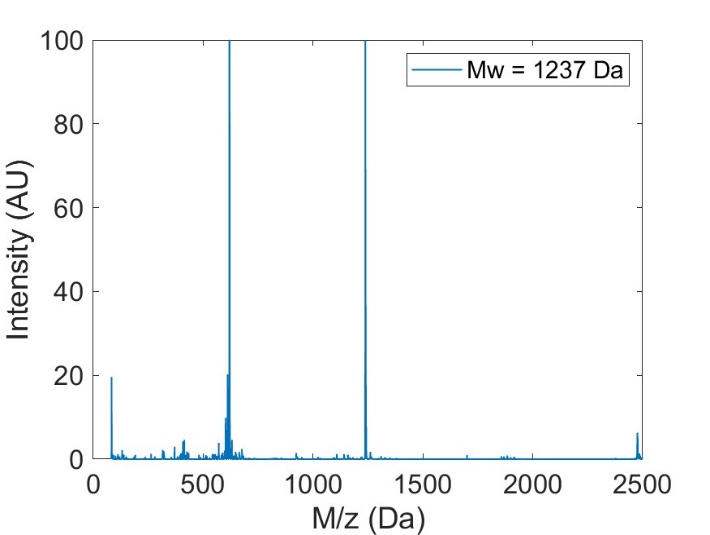


Biotin-Peptide 8:


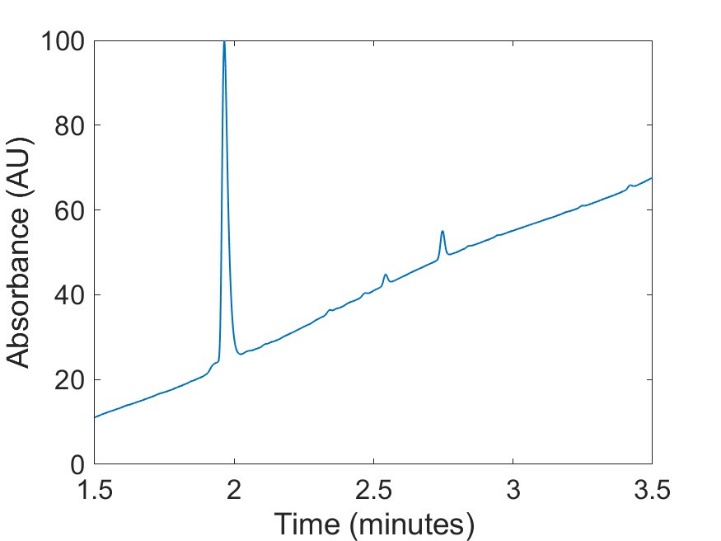

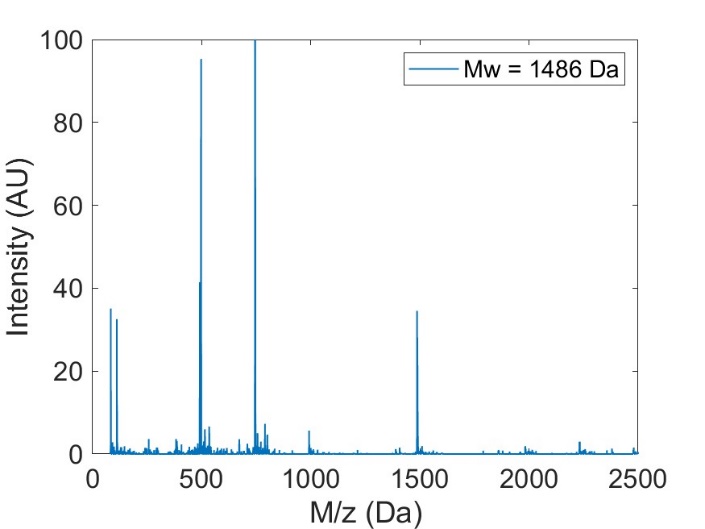

Supplement: Supplementary file 4 — Source data [file 41467_2023_42632_MOESM4_ESM.zip › Source Data/Purified peptide data/Supplementary File 1- purified peptides.docx]
