# Supplementary material for: A simple method for developing lysine targeted covalent protein reagents
Source: Nat Commun. 2023 Dec 1;14:7933. doi: 10.1038/s41467-023-42632-5 (PMC10692228; doi:10.1038/s41467-023-42632-5)

Peptide 1:

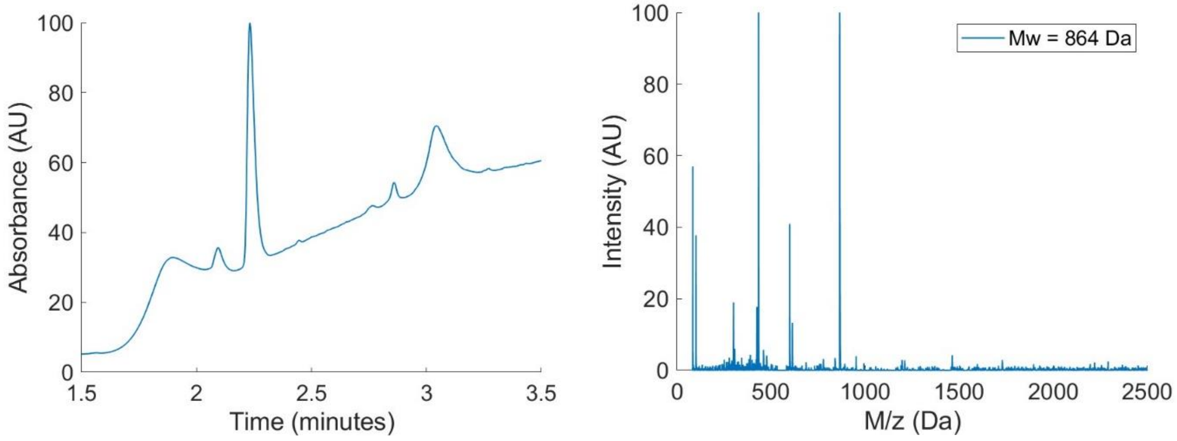

Peptide 2:

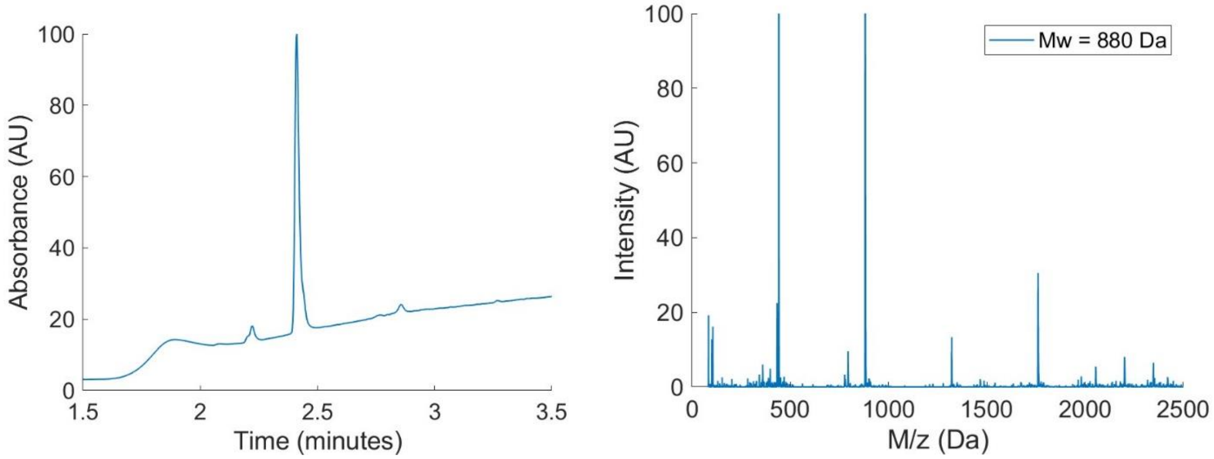

Peptide 3:

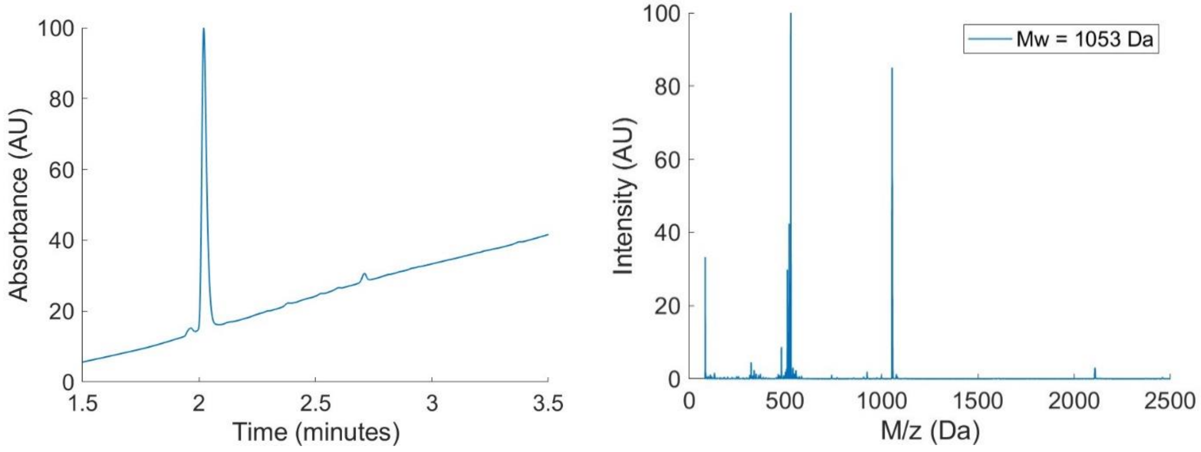

Peptide 4:

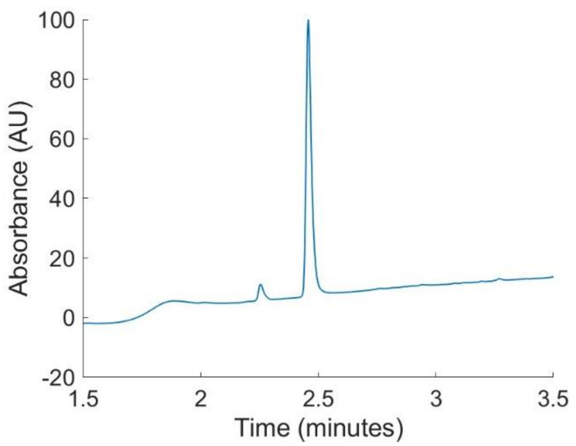

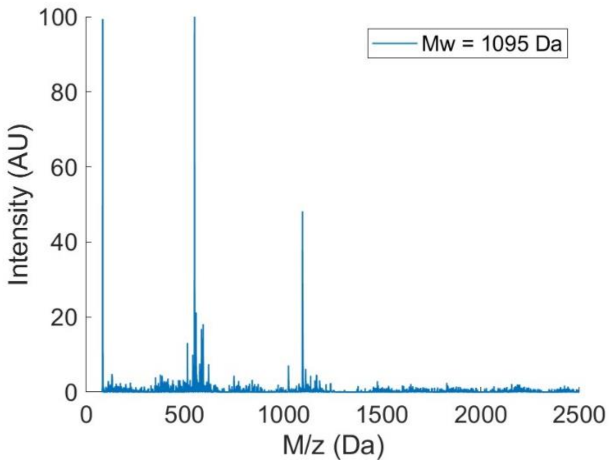

Peptide 5:

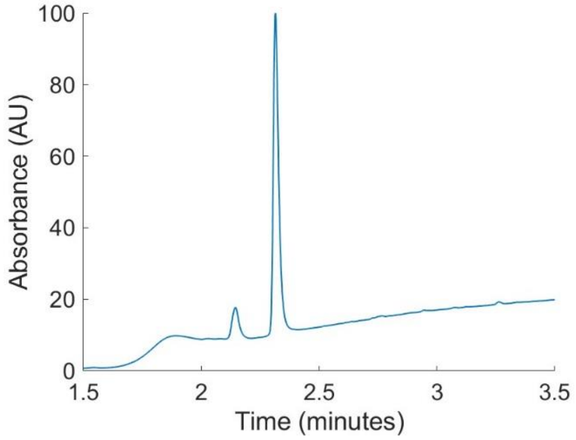

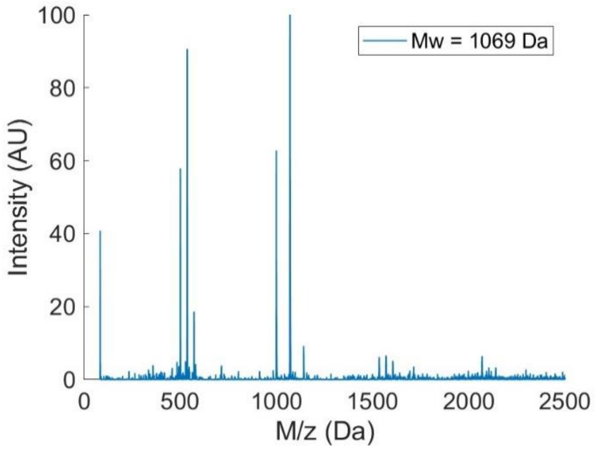

Peptide 6:

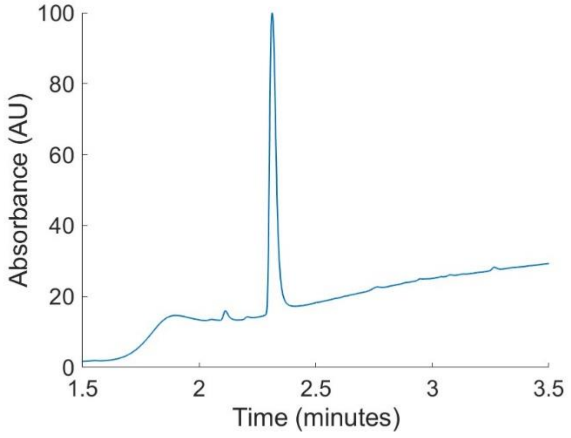

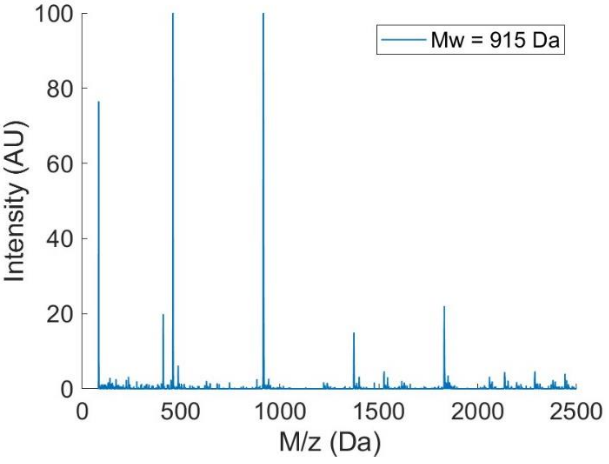

Peptide 7:

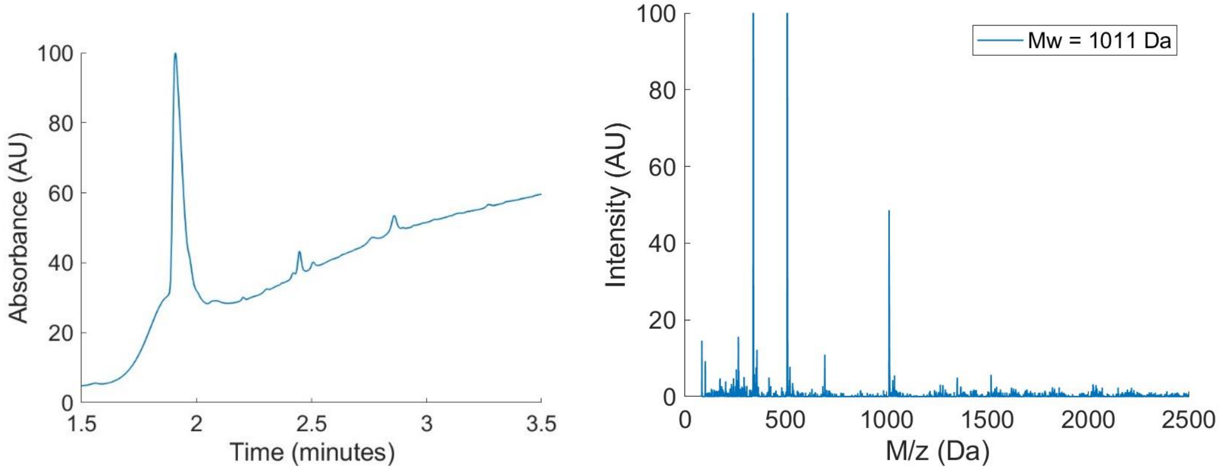

Peptide 8:

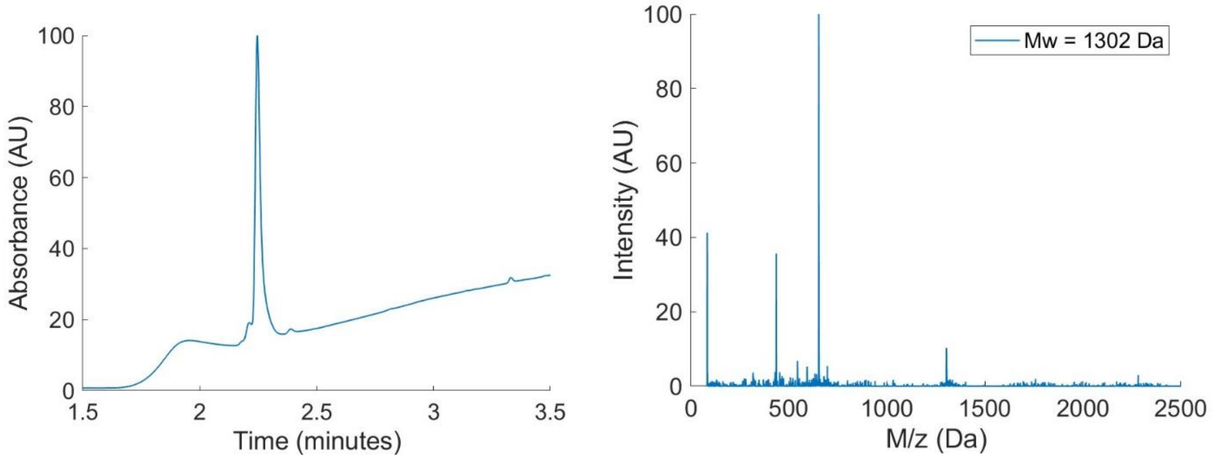

Peptide 9:

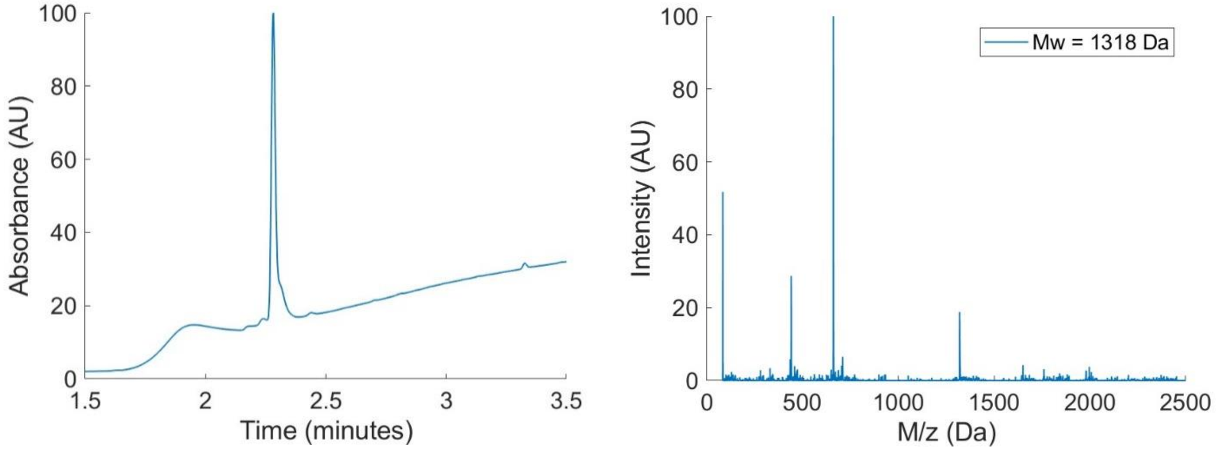

Peptide 10:

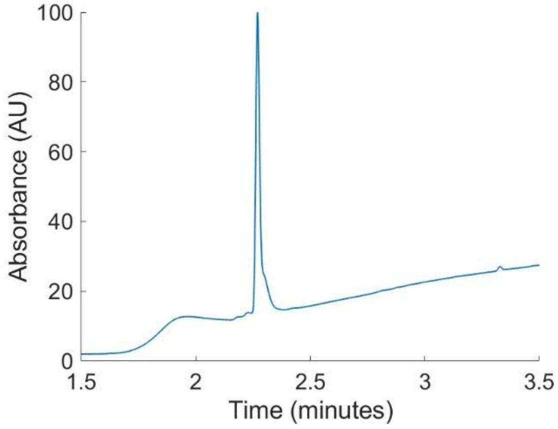

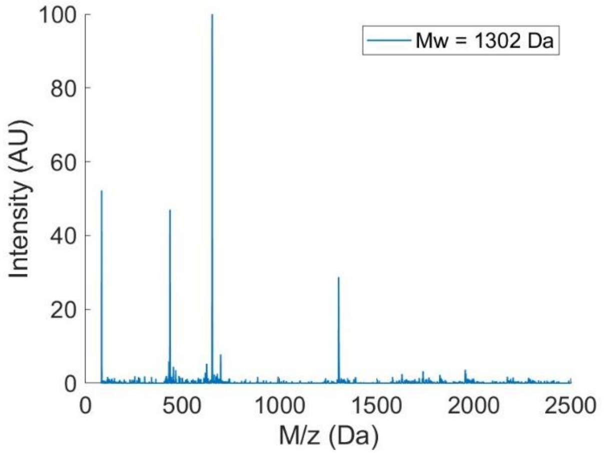

Peptide 11:

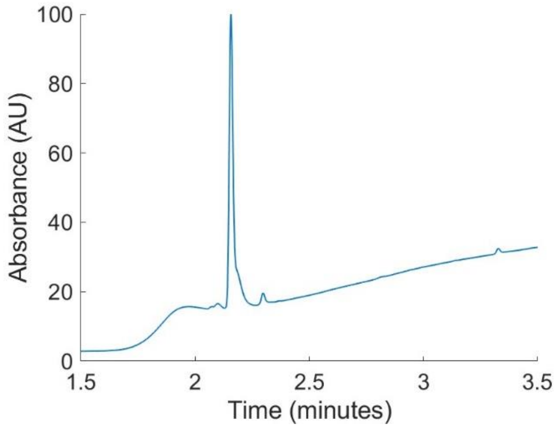

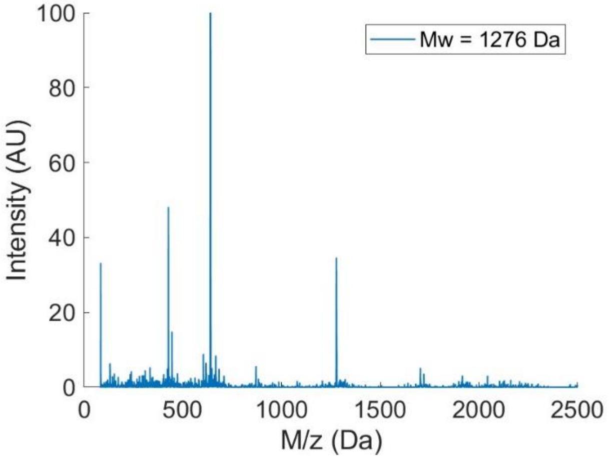

PropargylGly-peptide 3:

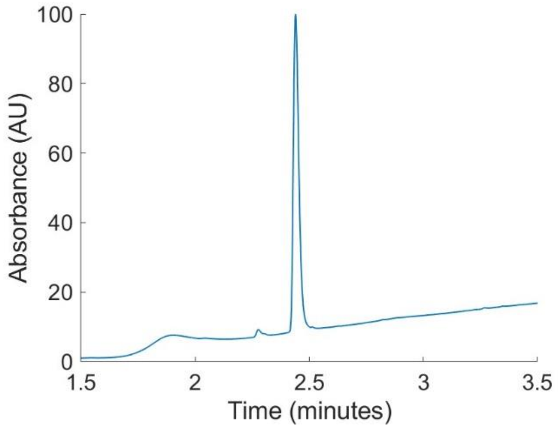

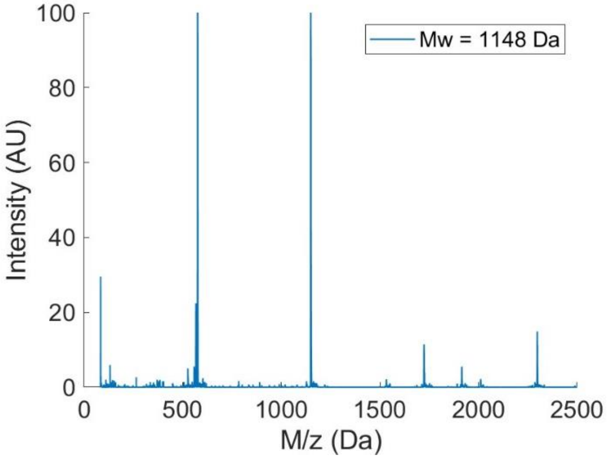

PropargylGly-peptide 8:

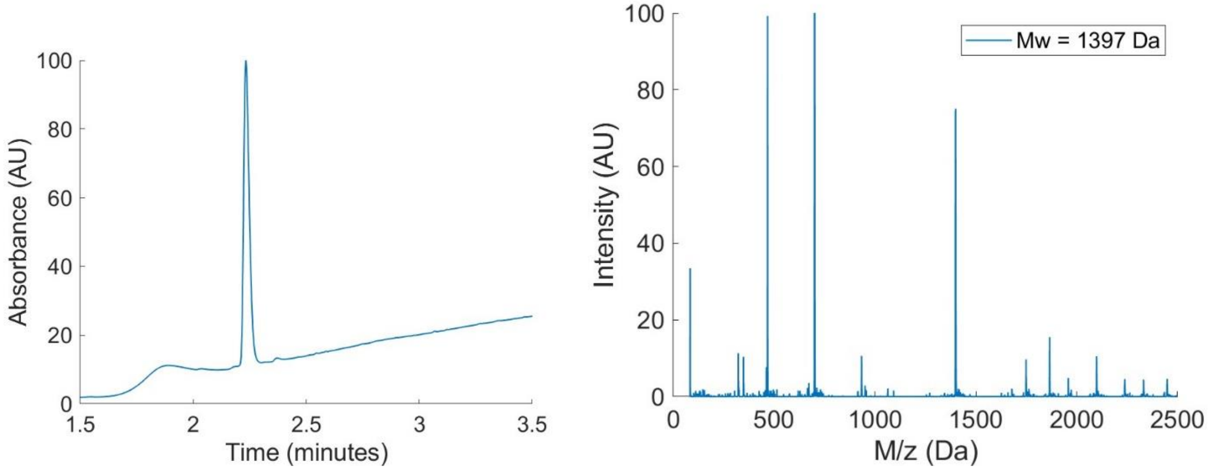

BDP-peptide 3:

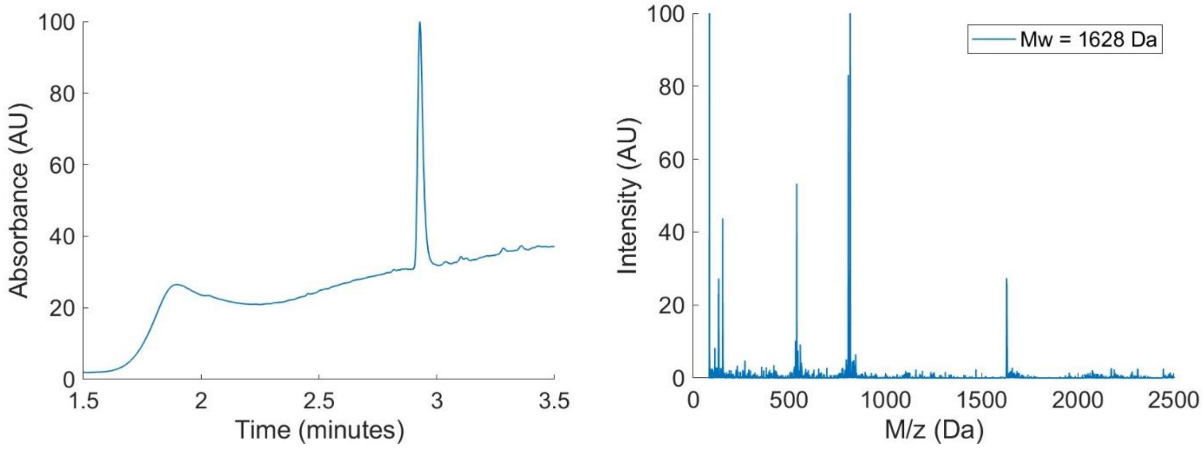

BDP-peptide 8:

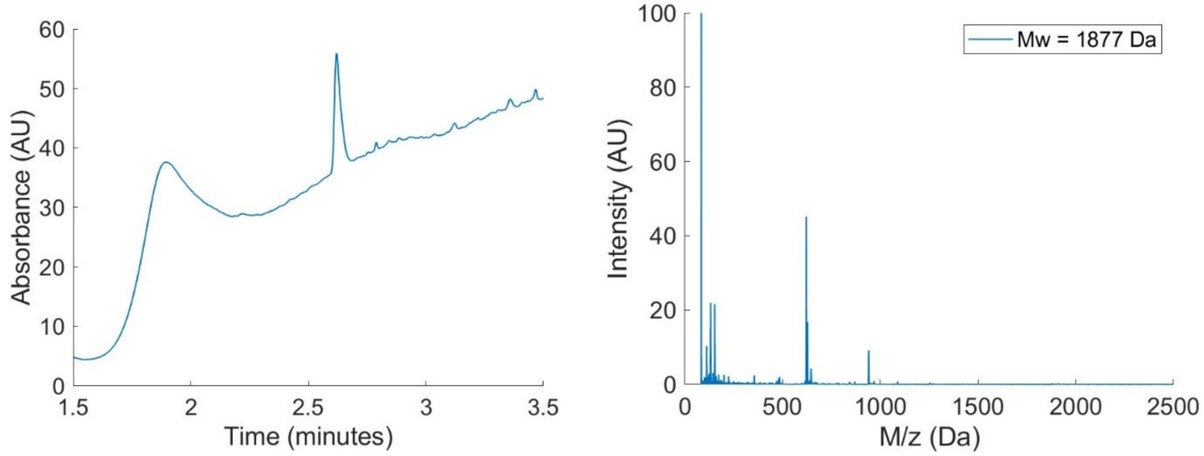

Biotin-Peptide 3:

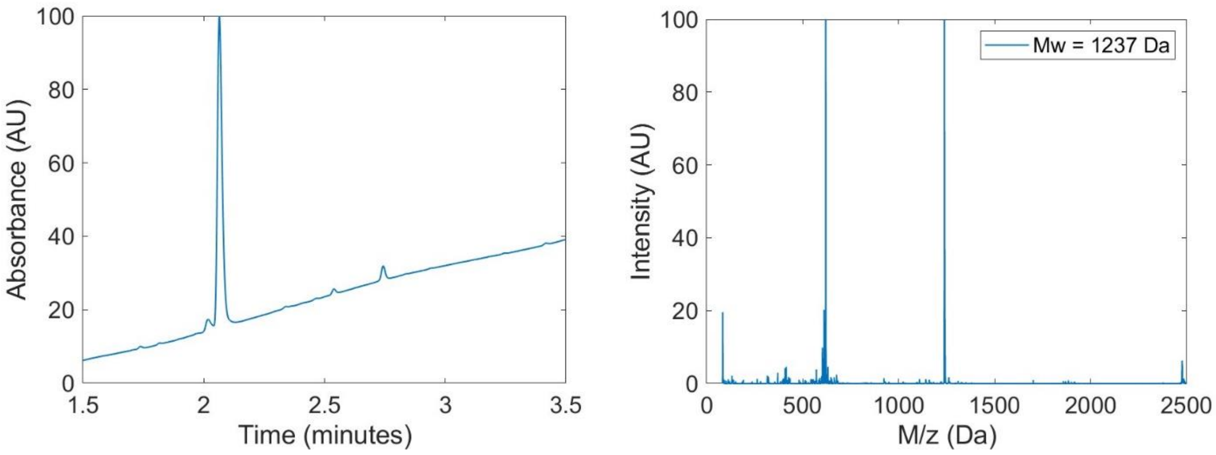

Biotin-Peptide 8:

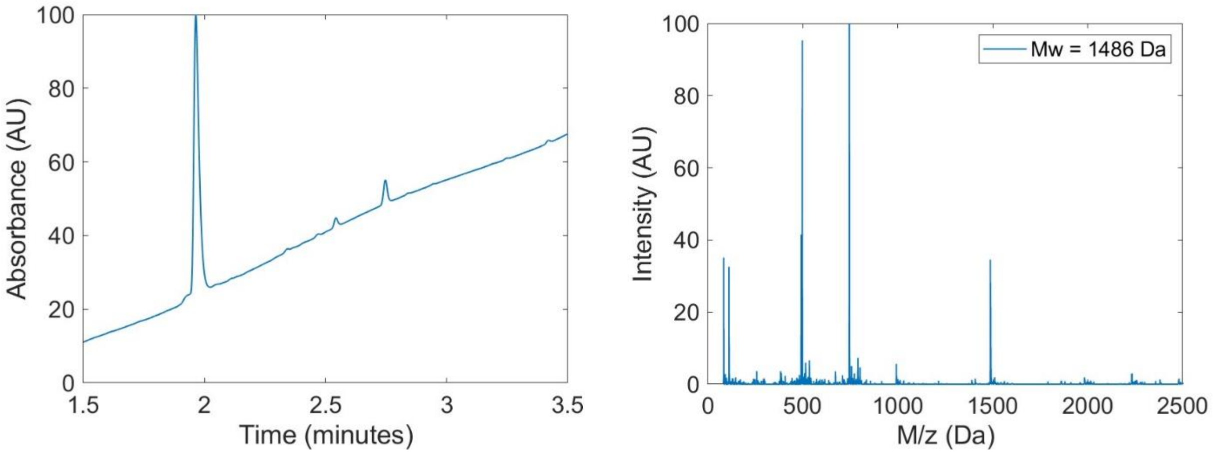

Supplement: Supplementary file 4 — Source data [file 41467_2023_42632_MOESM4_ESM.zip › Source Data/Purified peptide data/Supplementary File 1- purified peptides.pdf]
