# Supplementary figures and images for: A simple method for developing lysine targeted covalent protein reagents
Source: Nat Commun. 2023 Dec 1;14:7933. doi: 10.1038/s41467-023-42632-5 (PMC10692228; doi:10.1038/s41467-023-42632-5)

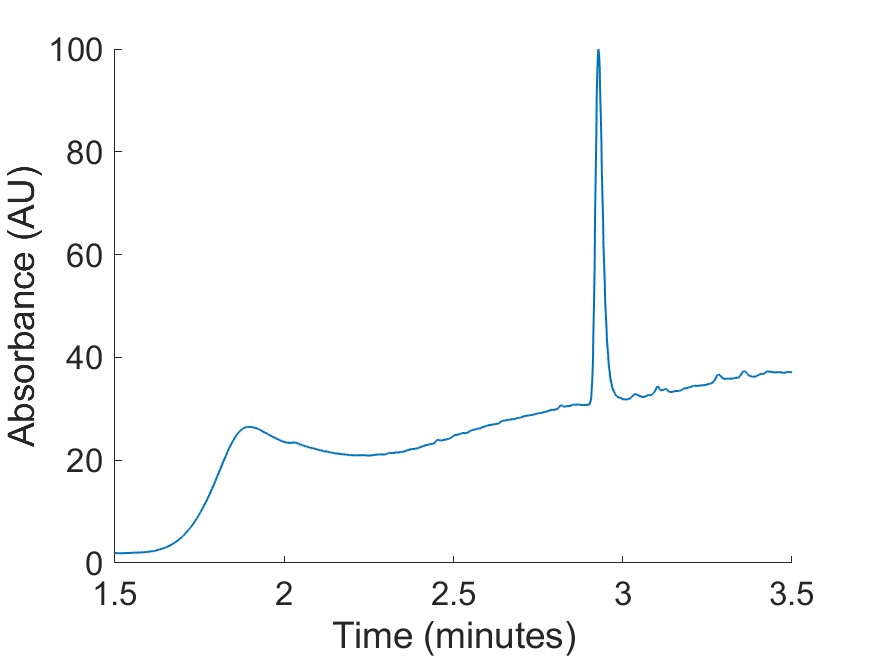

Supplement: Supplementary file 4 — Source data [file 41467_2023_42632_MOESM4_ESM.zip › Source Data/Purified peptide data/Chromatograms/BDP-propgly3.jpg]

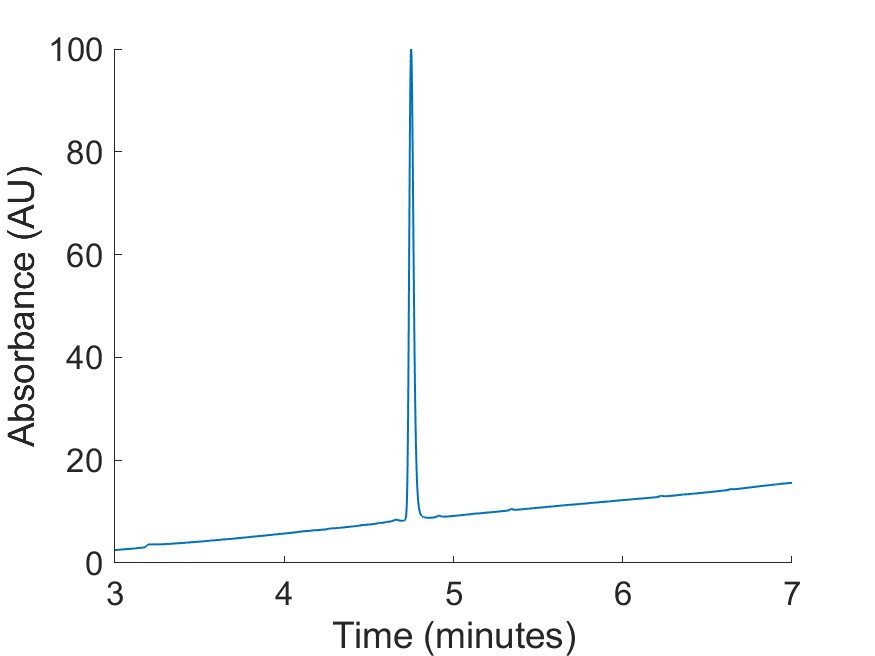

Supplement: Supplementary file 4 — Source data [file 41467_2023_42632_MOESM4_ESM.zip › Source Data/Purified peptide data/Chromatograms/BDP-propgly3ph.jpg]

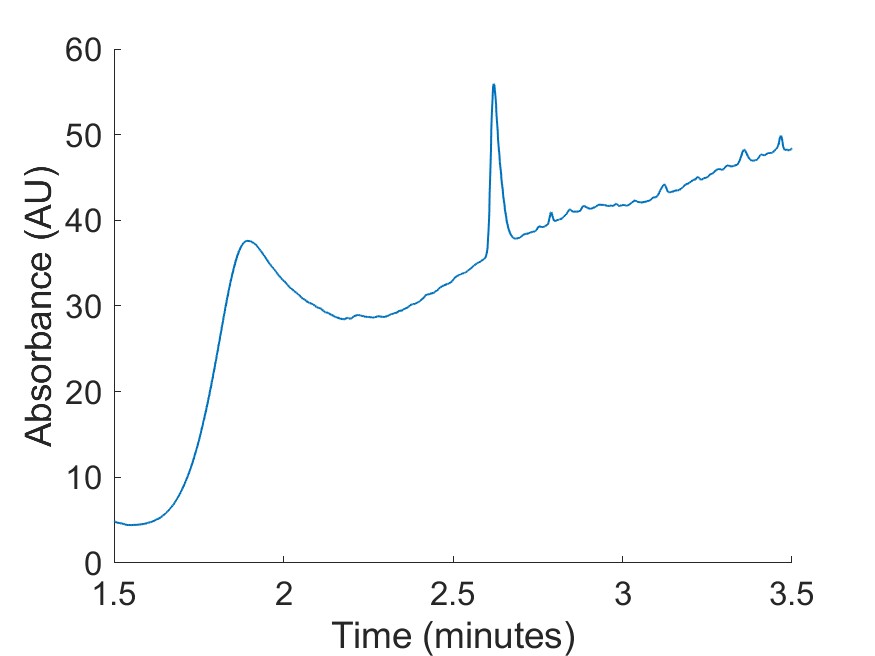

Supplement: Supplementary file 4 — Source data [file 41467_2023_42632_MOESM4_ESM.zip › Source Data/Purified peptide data/Chromatograms/BDP-propgly8.jpg]

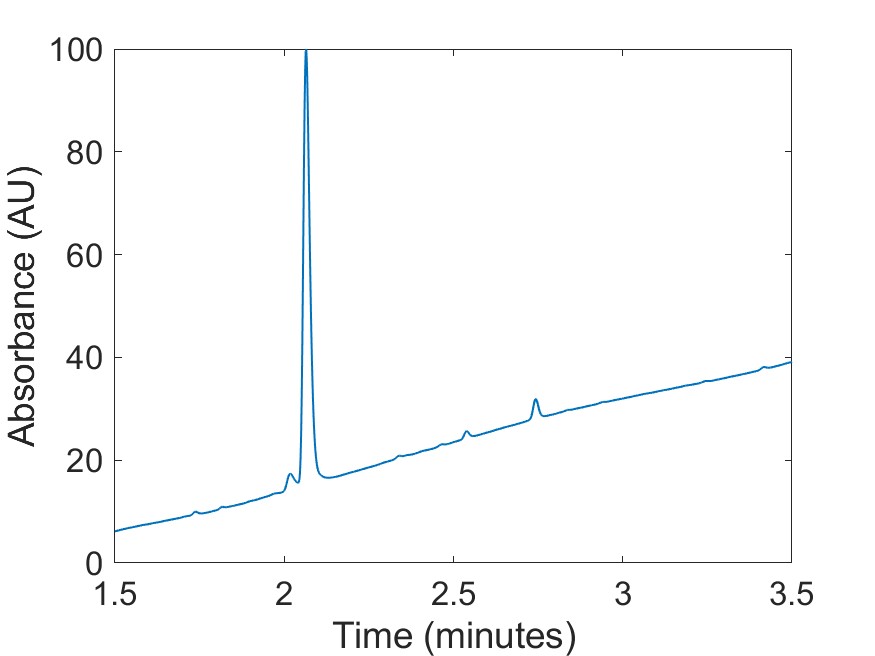

Supplement: Supplementary file 4 — Source data [file 41467_2023_42632_MOESM4_ESM.zip › Source Data/Purified peptide data/Chromatograms/Biotin-3.jpg]

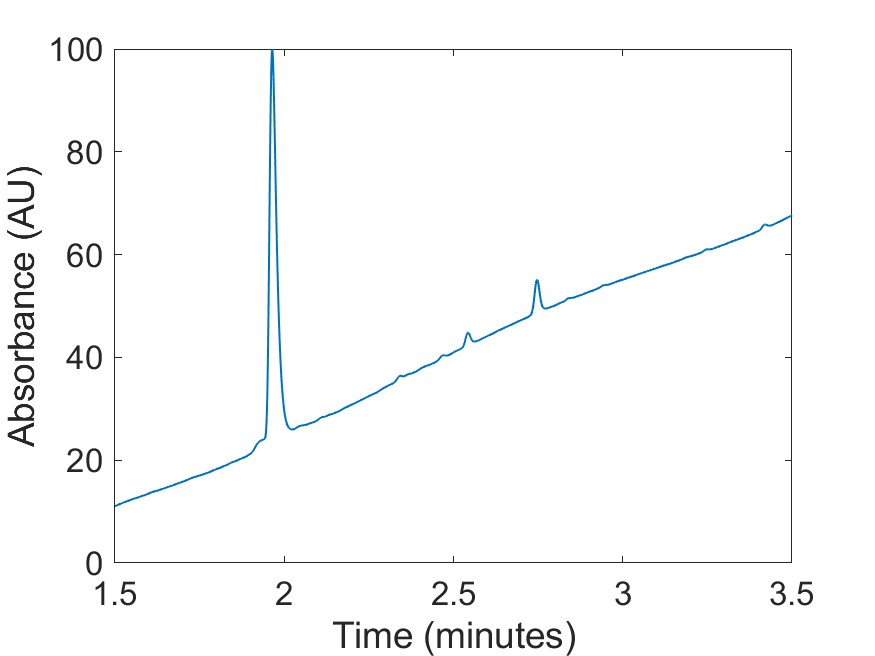

Supplement: Supplementary file 4 — Source data [file 41467_2023_42632_MOESM4_ESM.zip › Source Data/Purified peptide data/Chromatograms/Biotin-8.jpg]

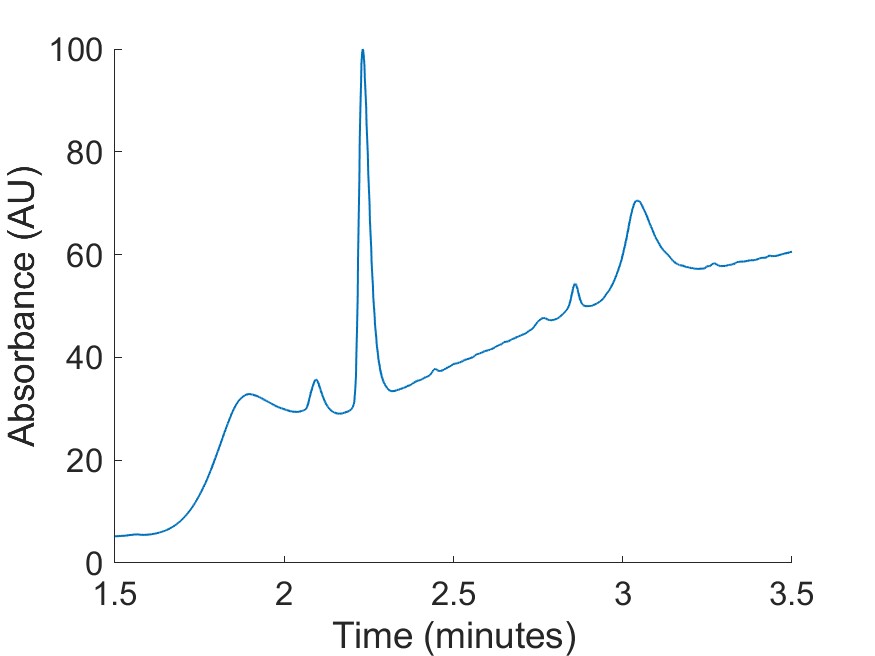

Supplement: Supplementary file 4 — Source data [file 41467_2023_42632_MOESM4_ESM.zip › Source Data/Purified peptide data/Chromatograms/Peptide_1.jpg]

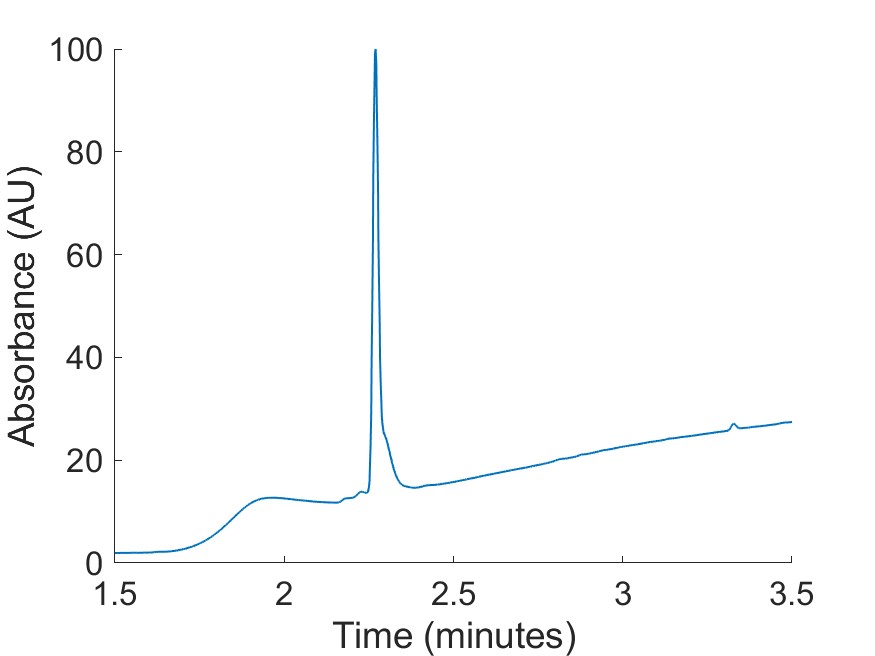

Supplement: Supplementary file 4 — Source data [file 41467_2023_42632_MOESM4_ESM.zip › Source Data/Purified peptide data/Chromatograms/Peptide_10.jpg]

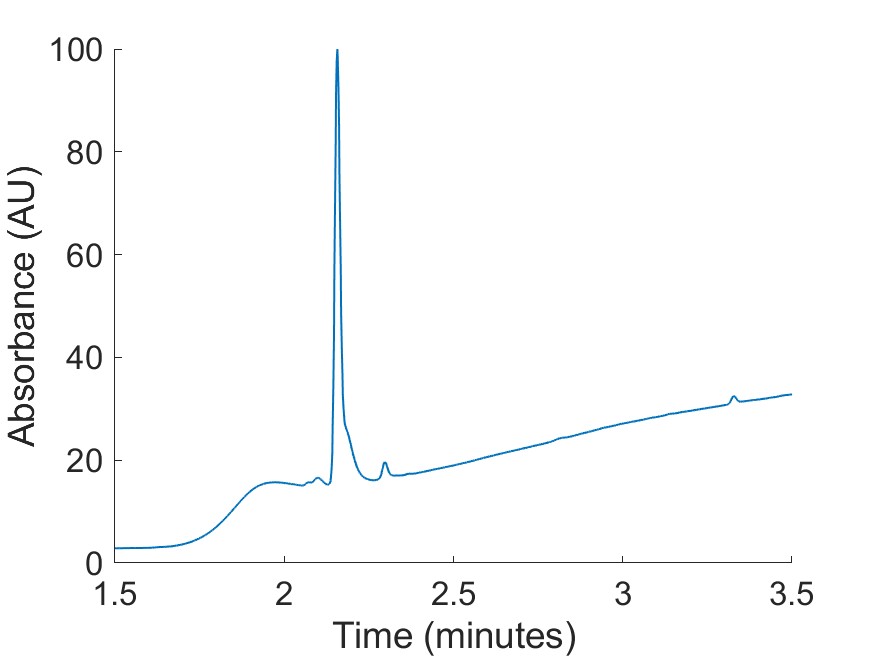

Supplement: Supplementary file 4 — Source data [file 41467_2023_42632_MOESM4_ESM.zip › Source Data/Purified peptide data/Chromatograms/Peptide_11.jpg]

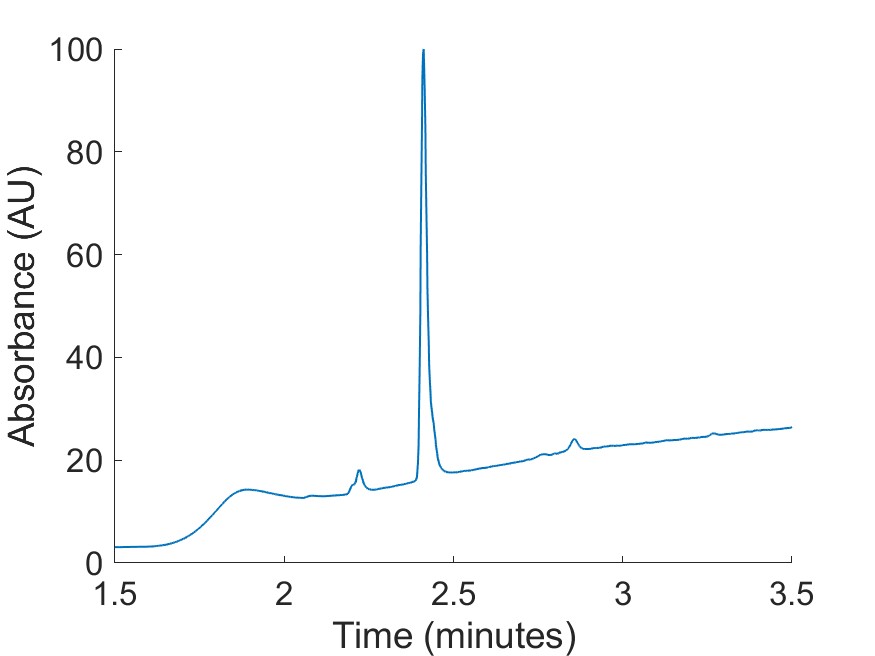

Supplement: Supplementary file 4 — Source data [file 41467_2023_42632_MOESM4_ESM.zip › Source Data/Purified peptide data/Chromatograms/Peptide_2.jpg]

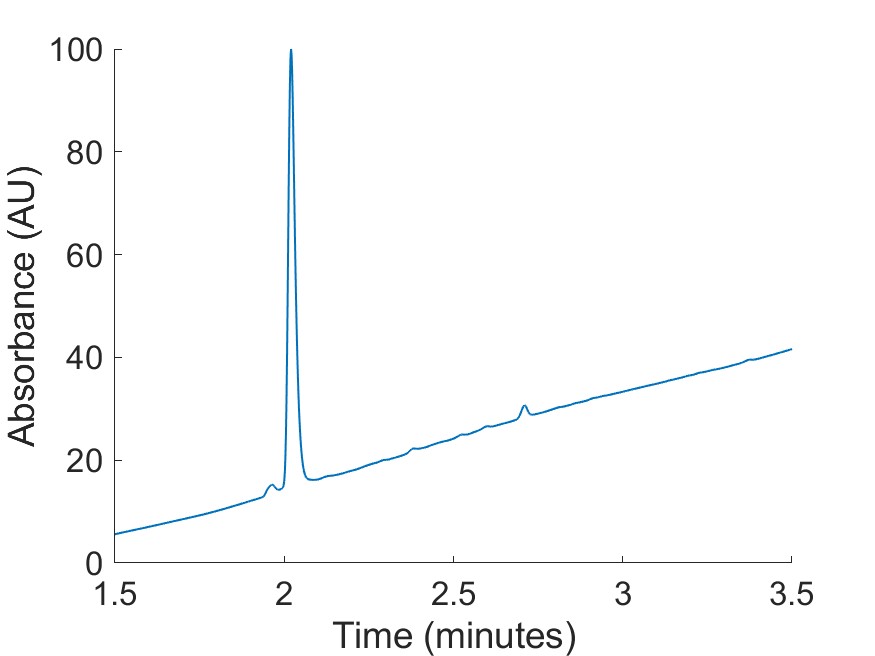

Supplement: Supplementary file 4 — Source data [file 41467_2023_42632_MOESM4_ESM.zip › Source Data/Purified peptide data/Chromatograms/Peptide_3.jpg]

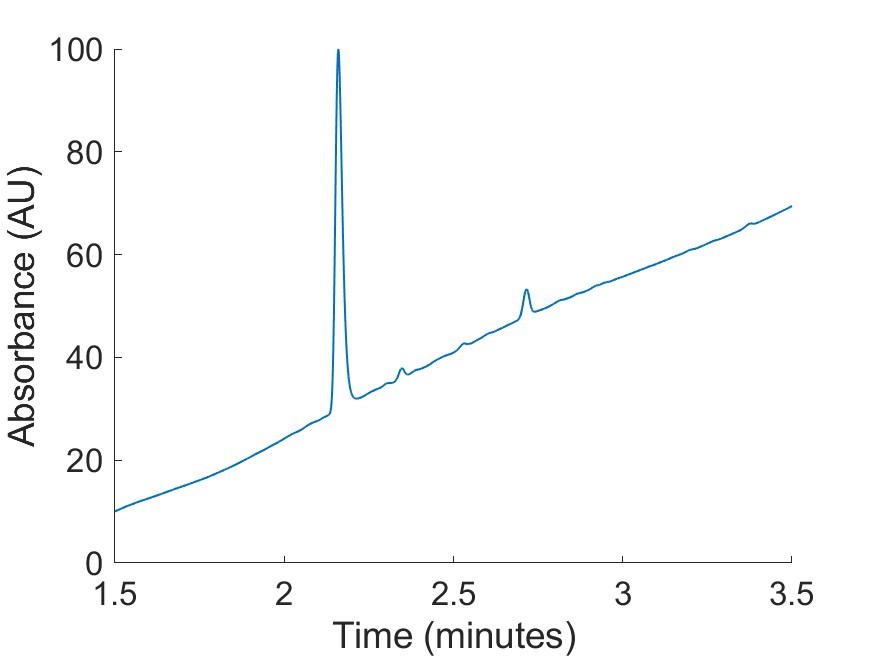

Supplement: Supplementary file 4 — Source data [file 41467_2023_42632_MOESM4_ESM.zip › Source Data/Purified peptide data/Chromatograms/Peptide_3ph.jpg]

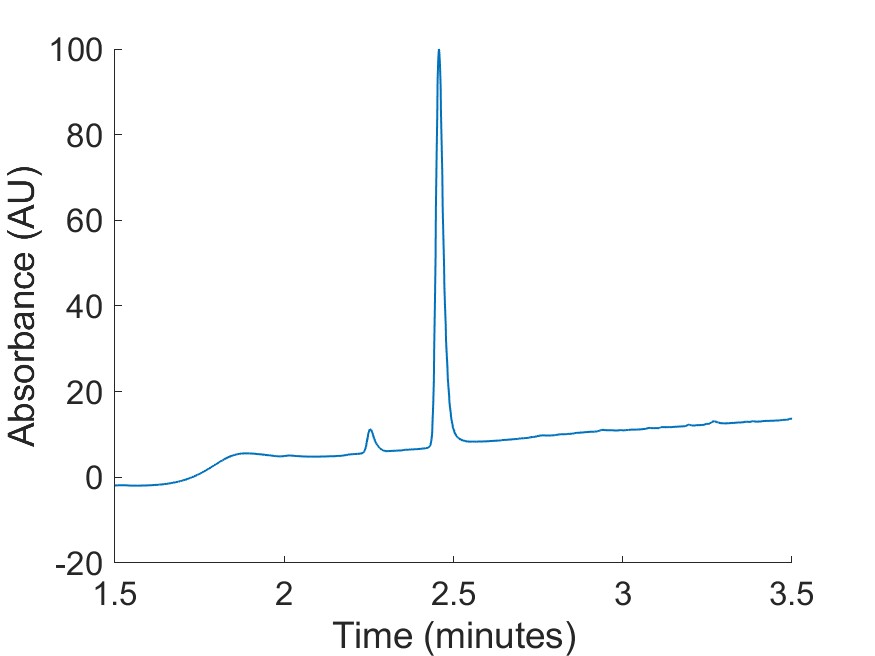

Supplement: Supplementary file 4 — Source data [file 41467_2023_42632_MOESM4_ESM.zip › Source Data/Purified peptide data/Chromatograms/Peptide_4.jpg]

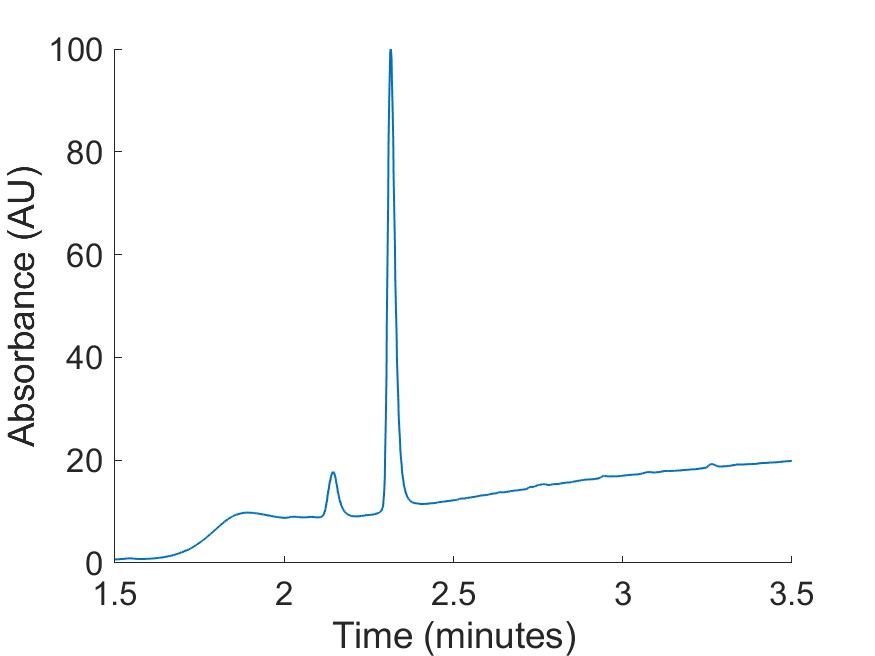

Supplement: Supplementary file 4 — Source data [file 41467_2023_42632_MOESM4_ESM.zip › Source Data/Purified peptide data/Chromatograms/Peptide_5.jpg]

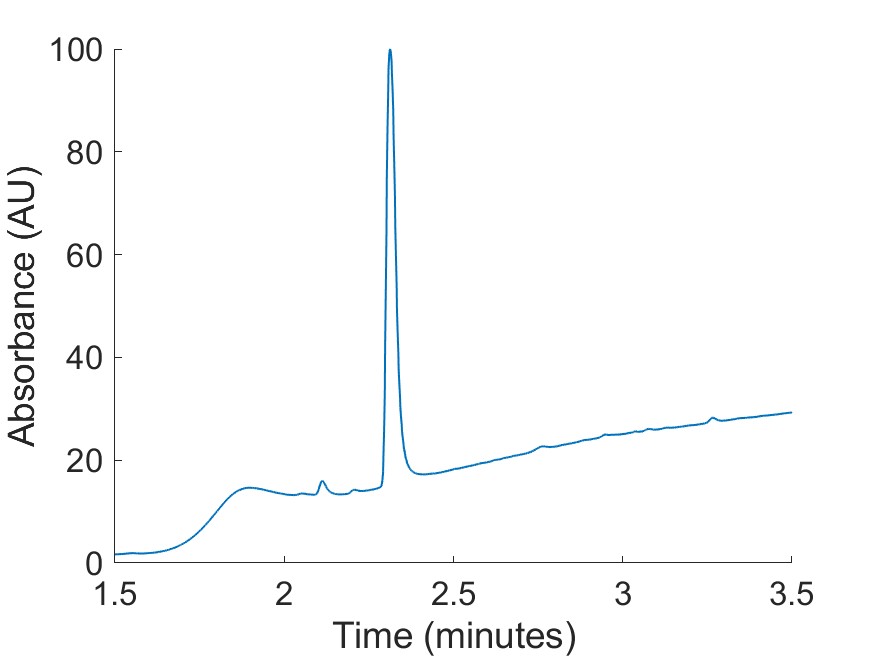

Supplement: Supplementary file 4 — Source data [file 41467_2023_42632_MOESM4_ESM.zip › Source Data/Purified peptide data/Chromatograms/Peptide_6.jpg]

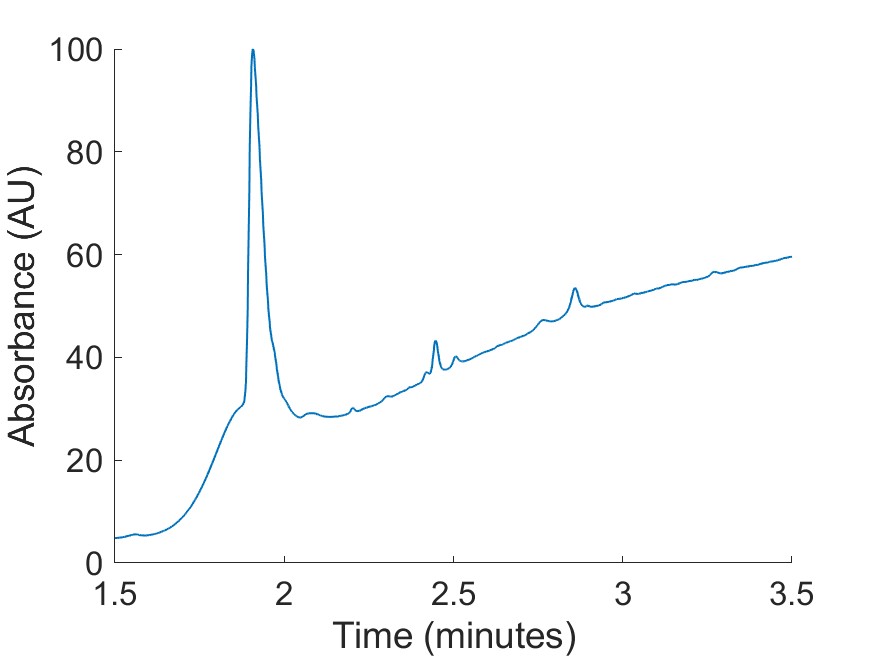

Supplement: Supplementary file 4 — Source data [file 41467_2023_42632_MOESM4_ESM.zip › Source Data/Purified peptide data/Chromatograms/Peptide_7.jpg]

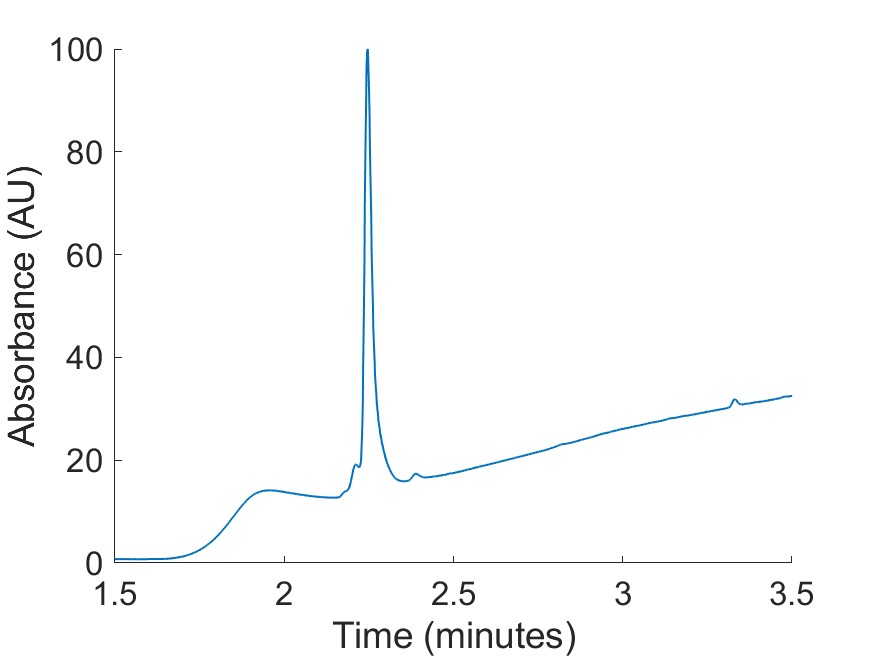

Supplement: Supplementary file 4 — Source data [file 41467_2023_42632_MOESM4_ESM.zip › Source Data/Purified peptide data/Chromatograms/Peptide_8.jpg]

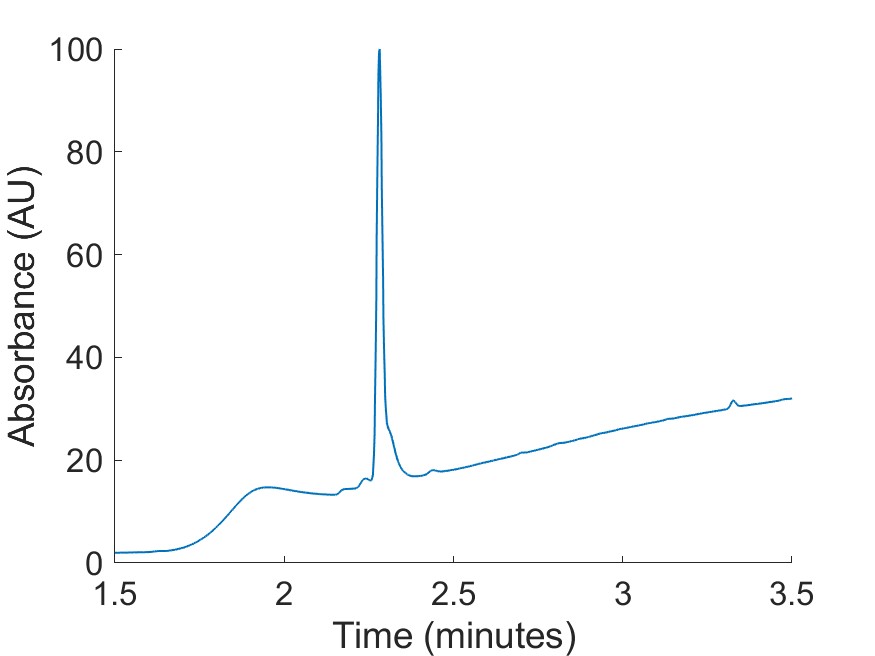

Supplement: Supplementary file 4 — Source data [file 41467_2023_42632_MOESM4_ESM.zip › Source Data/Purified peptide data/Chromatograms/Peptide_9.jpg]

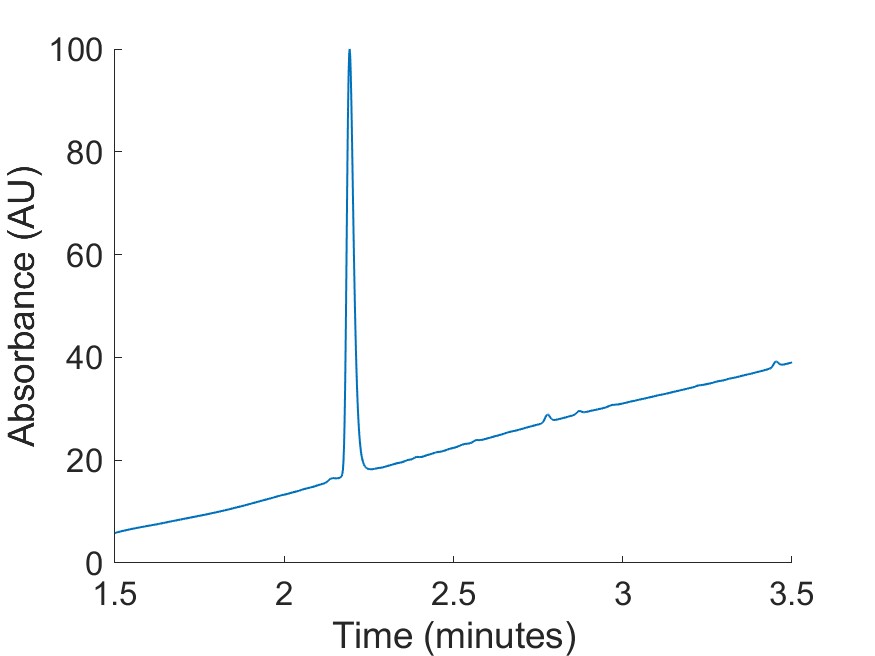

Supplement: Supplementary file 4 — Source data [file 41467_2023_42632_MOESM4_ESM.zip › Source Data/Purified peptide data/Chromatograms/PropGly-3ph.jpg]

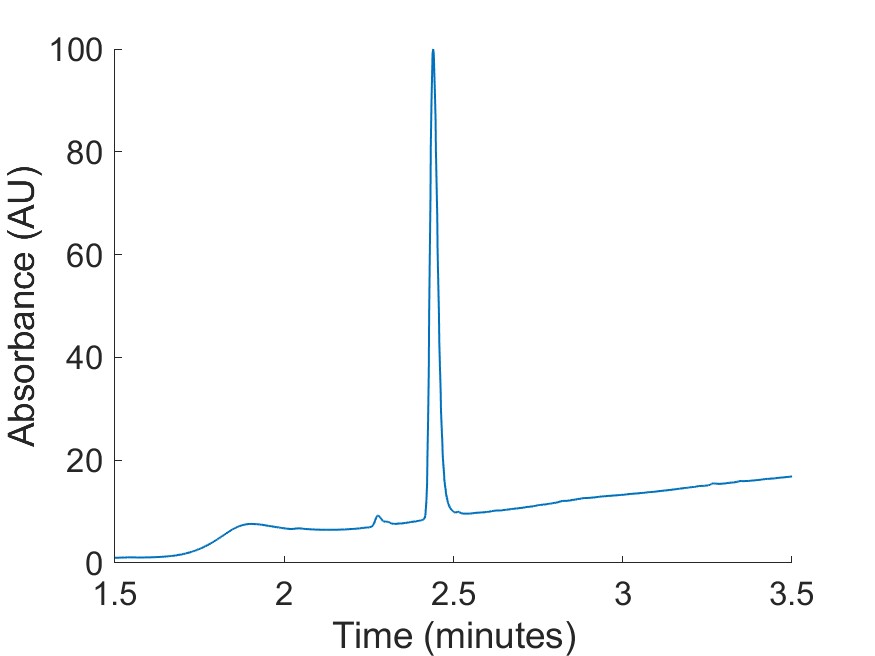

Supplement: Supplementary file 4 — Source data [file 41467_2023_42632_MOESM4_ESM.zip › Source Data/Purified peptide data/Chromatograms/Propgly3.jpg]

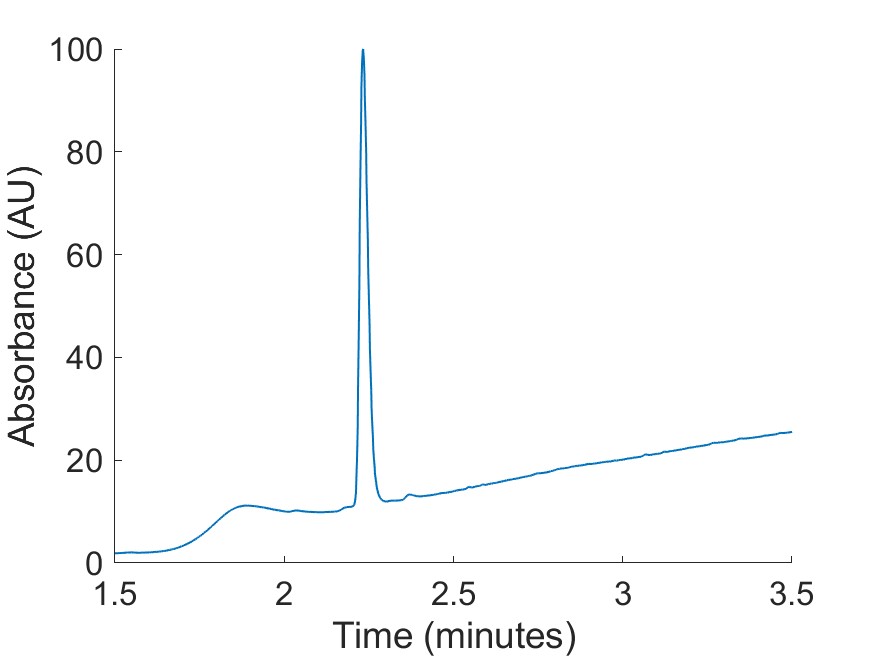

Supplement: Supplementary file 4 — Source data [file 41467_2023_42632_MOESM4_ESM.zip › Source Data/Purified peptide data/Chromatograms/Propgly8.jpg]

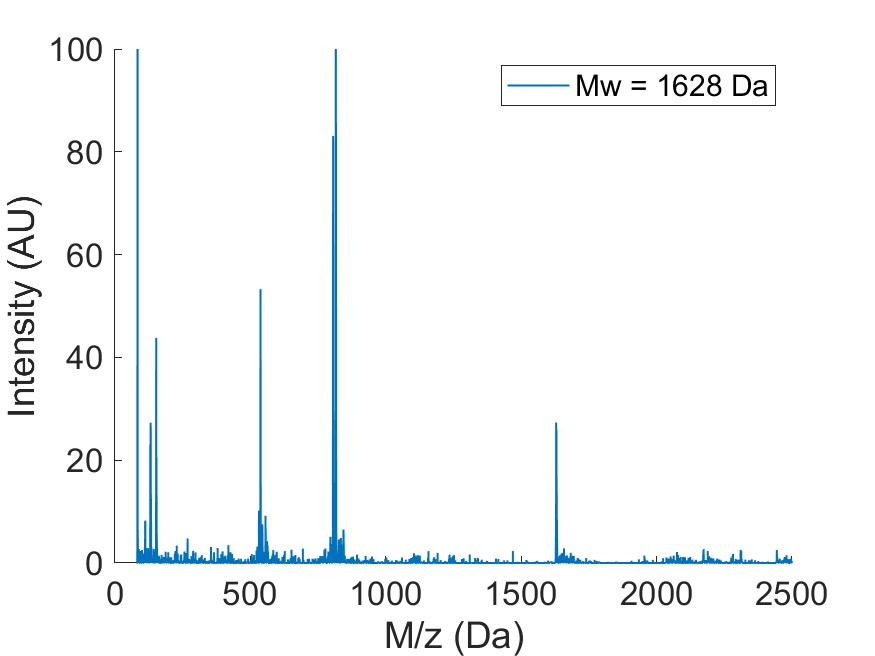

Supplement: Supplementary file 4 — Source data [file 41467_2023_42632_MOESM4_ESM.zip › Source Data/Purified peptide data/Spectra/BDP-Propgly3.jpg]

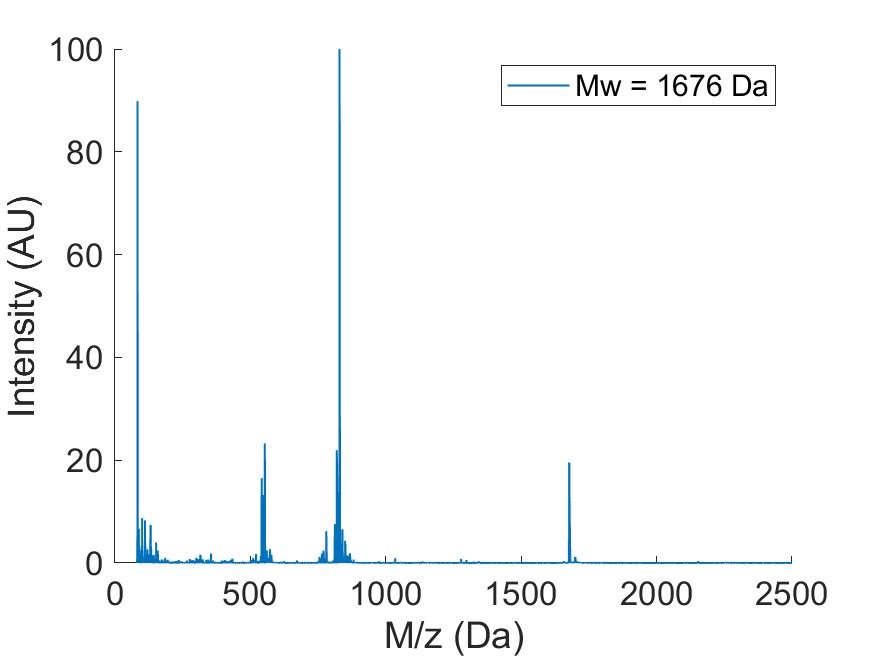

Supplement: Supplementary file 4 — Source data [file 41467_2023_42632_MOESM4_ESM.zip › Source Data/Purified peptide data/Spectra/BDP-Propgly3ph.jpg]

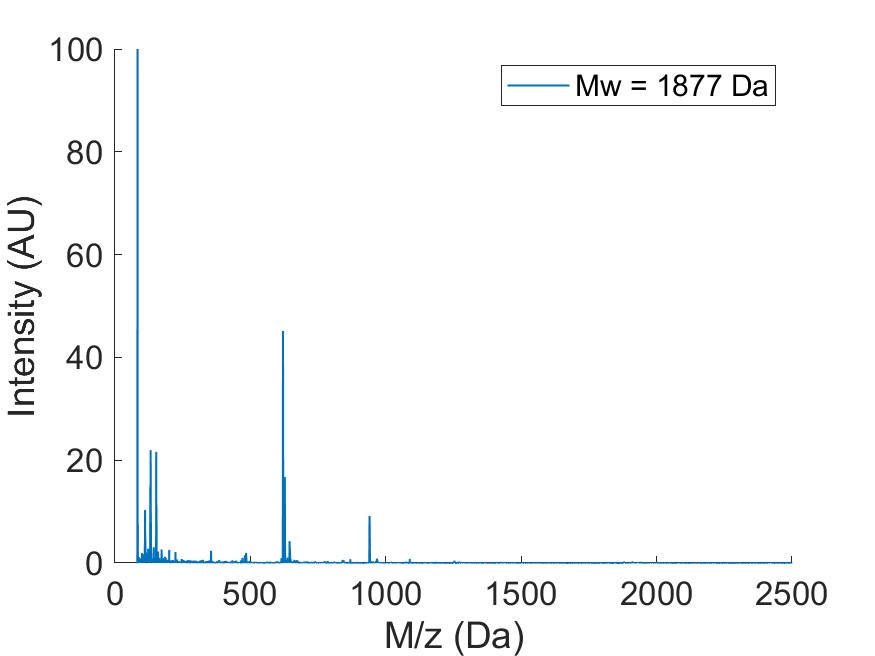

Supplement: Supplementary file 4 — Source data [file 41467_2023_42632_MOESM4_ESM.zip › Source Data/Purified peptide data/Spectra/BDP-Propgly8.jpg]

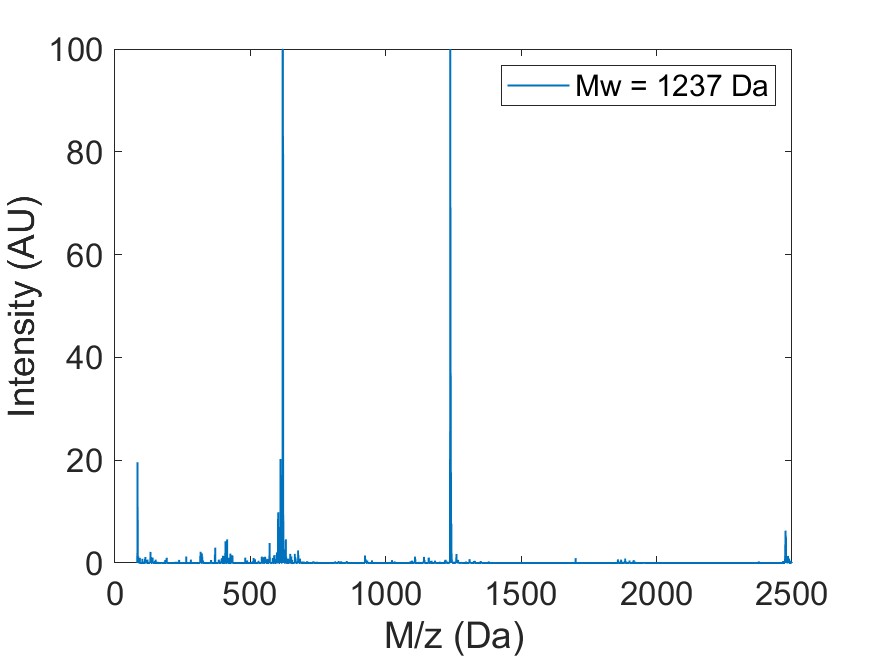

Supplement: Supplementary file 4 — Source data [file 41467_2023_42632_MOESM4_ESM.zip › Source Data/Purified peptide data/Spectra/Biotin-3.jpg]

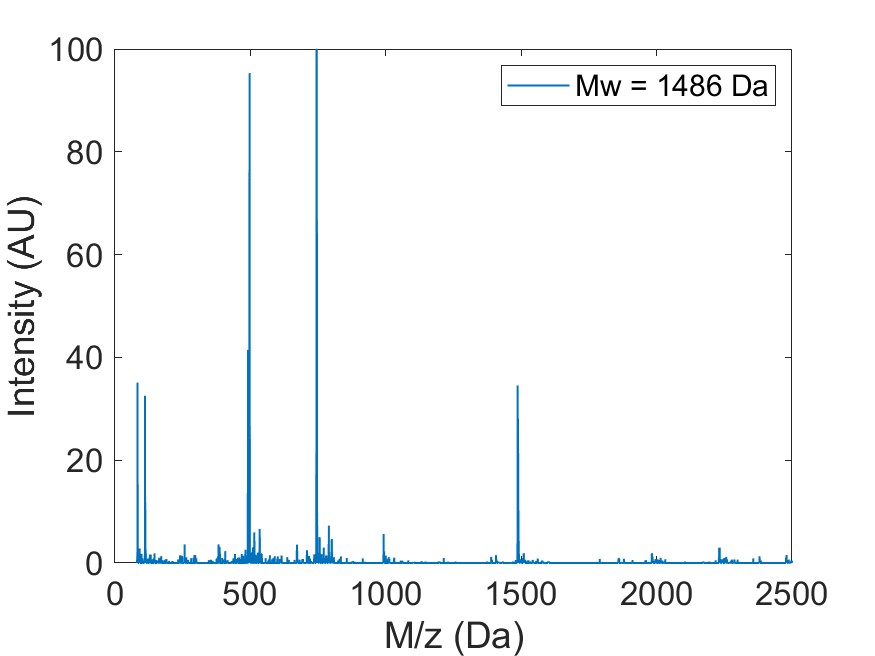

Supplement: Supplementary file 4 — Source data [file 41467_2023_42632_MOESM4_ESM.zip › Source Data/Purified peptide data/Spectra/Biotin-8.jpg]

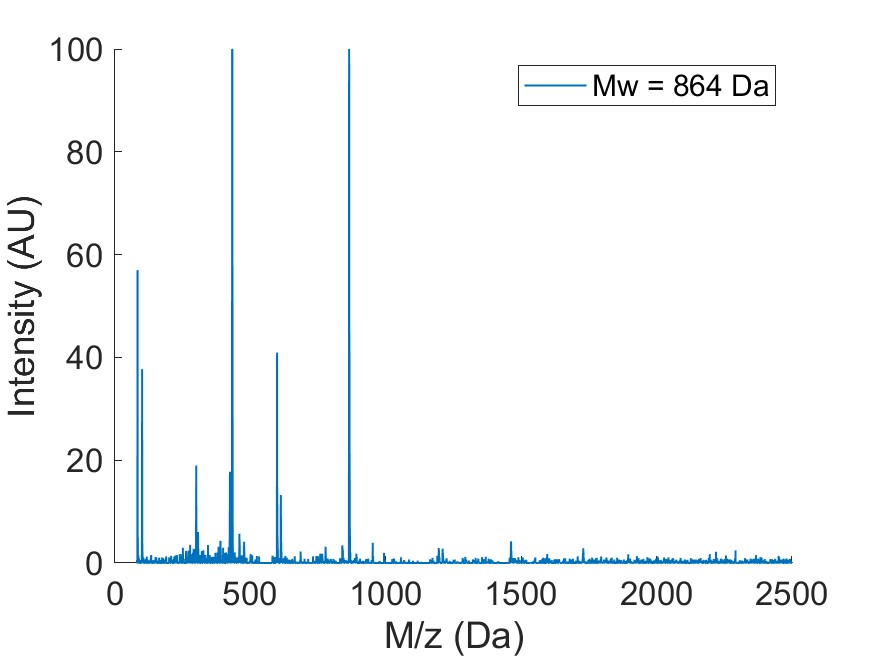

Supplement: Supplementary file 4 — Source data [file 41467_2023_42632_MOESM4_ESM.zip › Source Data/Purified peptide data/Spectra/Peptide 1.jpg]

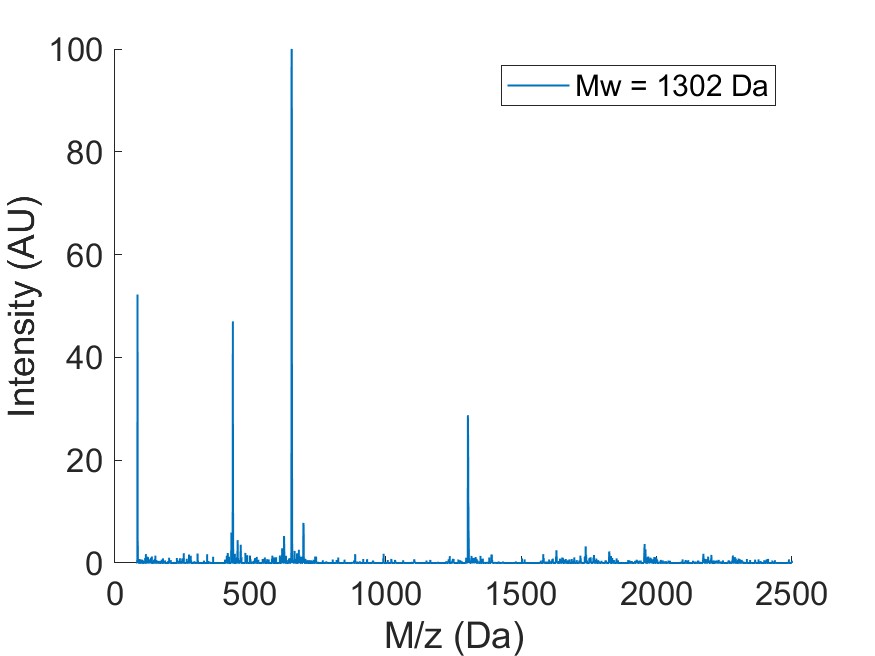

Supplement: Supplementary file 4 — Source data [file 41467_2023_42632_MOESM4_ESM.zip › Source Data/Purified peptide data/Spectra/Peptide 10.jpg]

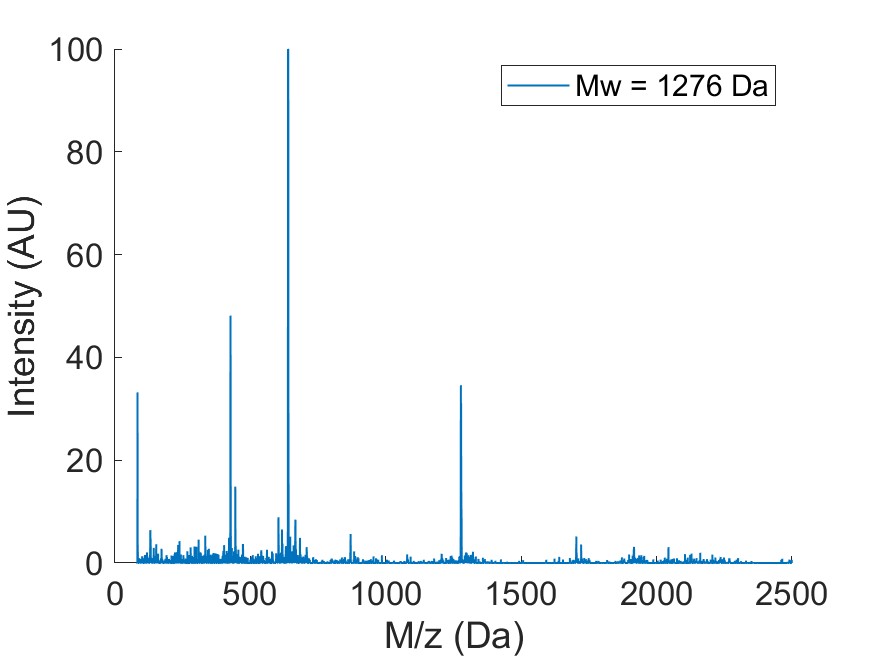

Supplement: Supplementary file 4 — Source data [file 41467_2023_42632_MOESM4_ESM.zip › Source Data/Purified peptide data/Spectra/Peptide 11.jpg]

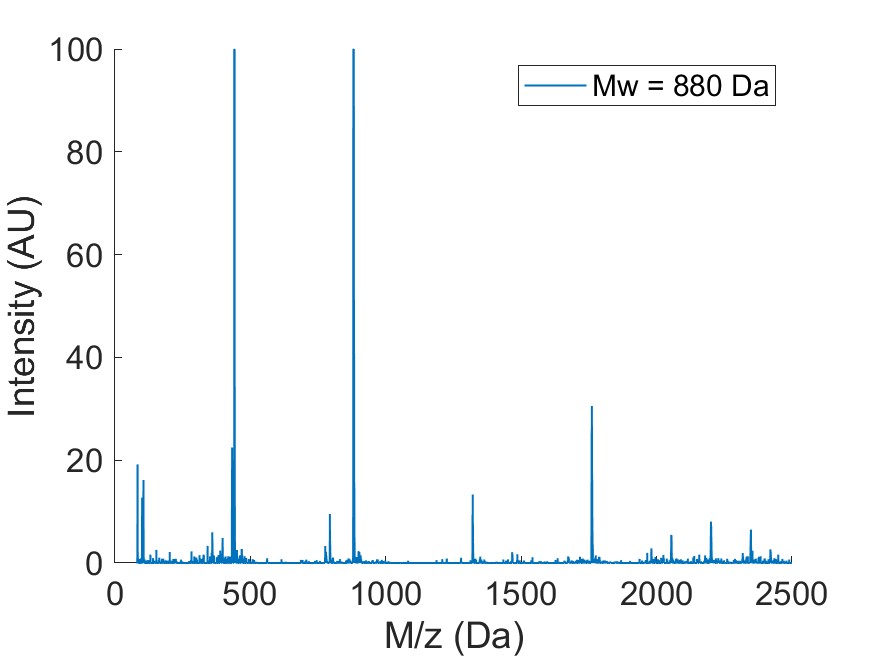

Supplement: Supplementary file 4 — Source data [file 41467_2023_42632_MOESM4_ESM.zip › Source Data/Purified peptide data/Spectra/Peptide 2.jpg]

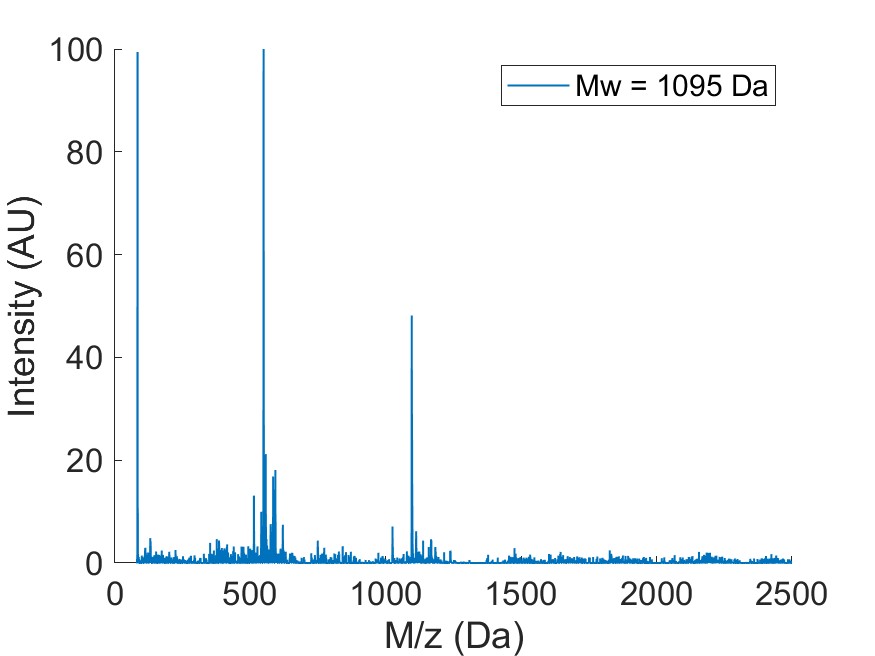

Supplement: Supplementary file 4 — Source data [file 41467_2023_42632_MOESM4_ESM.zip › Source Data/Purified peptide data/Spectra/Peptide 4.jpg]

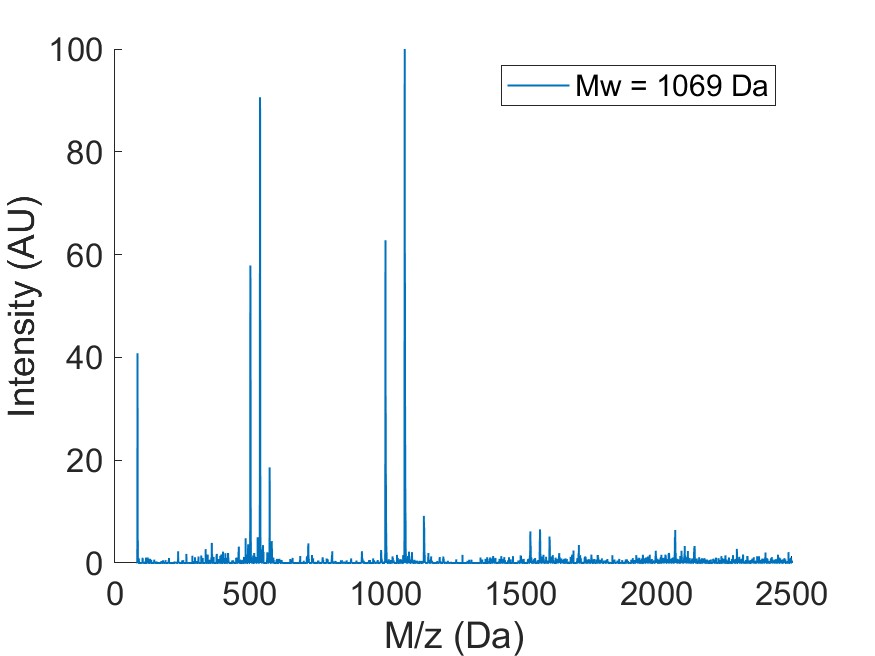

Supplement: Supplementary file 4 — Source data [file 41467_2023_42632_MOESM4_ESM.zip › Source Data/Purified peptide data/Spectra/Peptide 5.jpg]

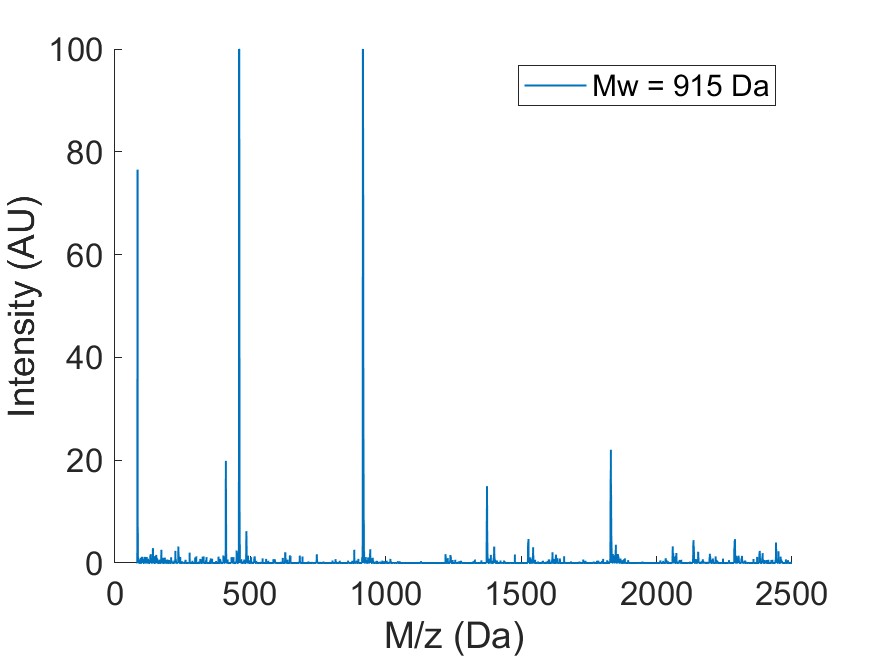

Supplement: Supplementary file 4 — Source data [file 41467_2023_42632_MOESM4_ESM.zip › Source Data/Purified peptide data/Spectra/Peptide 6.jpg]

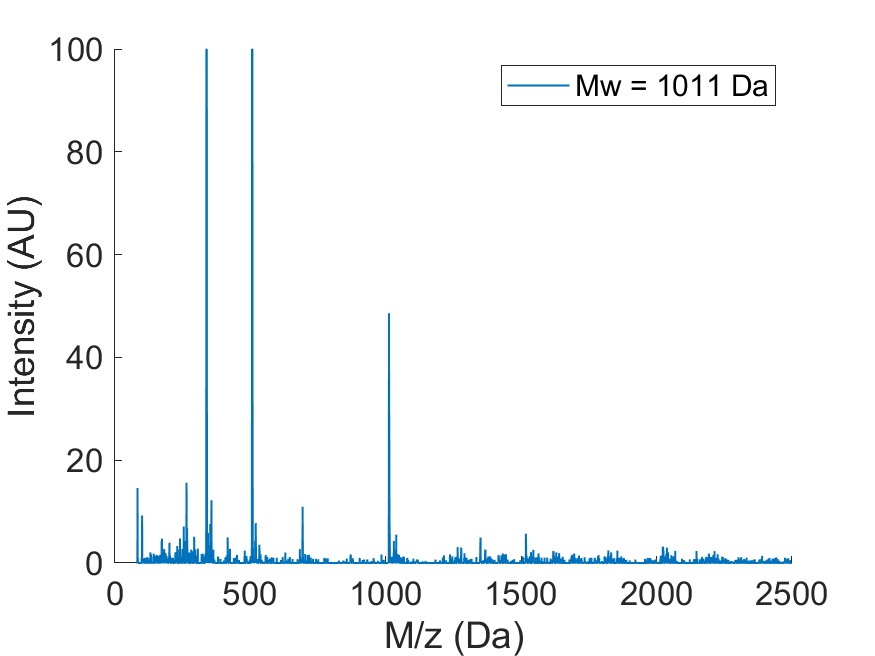

Supplement: Supplementary file 4 — Source data [file 41467_2023_42632_MOESM4_ESM.zip › Source Data/Purified peptide data/Spectra/Peptide 7.jpg]

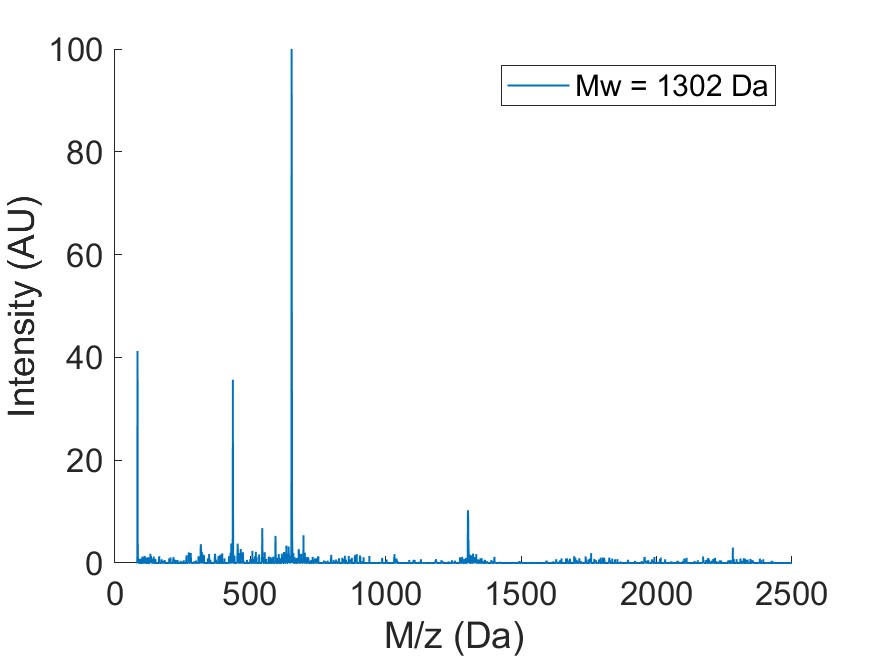

Supplement: Supplementary file 4 — Source data [file 41467_2023_42632_MOESM4_ESM.zip › Source Data/Purified peptide data/Spectra/Peptide 8.jpg]

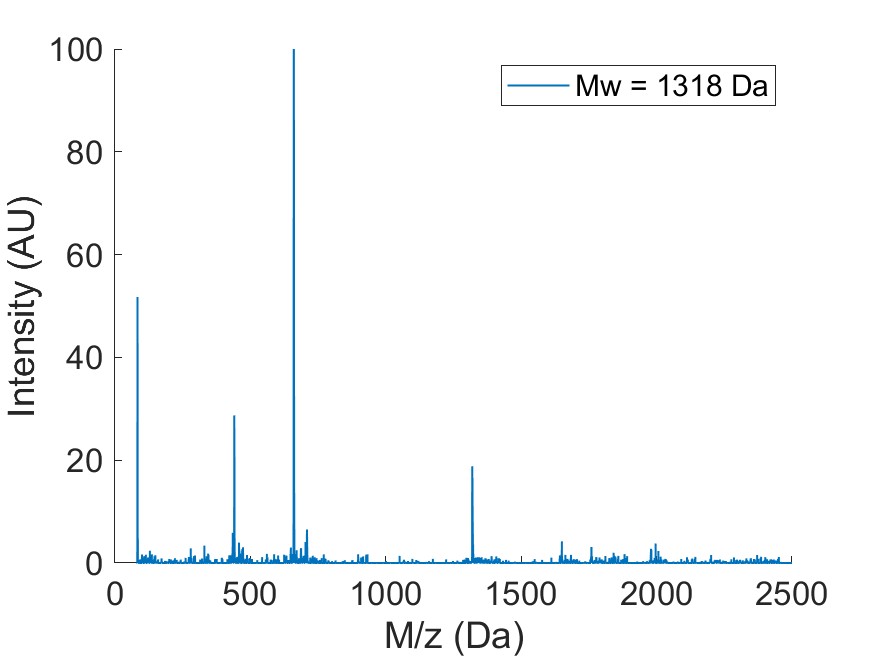

Supplement: Supplementary file 4 — Source data [file 41467_2023_42632_MOESM4_ESM.zip › Source Data/Purified peptide data/Spectra/Peptide 9.jpg]

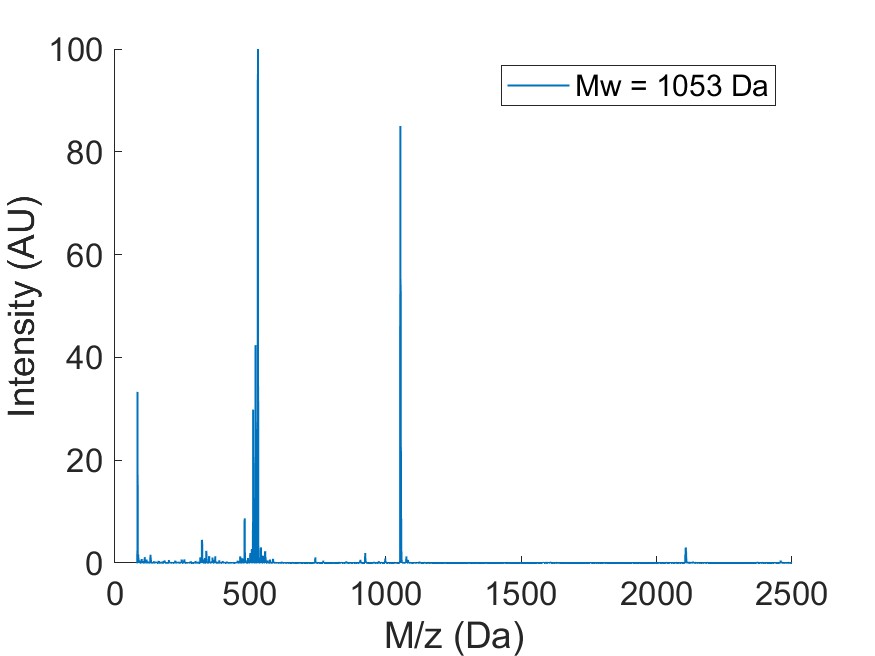

Supplement: Supplementary file 4 — Source data [file 41467_2023_42632_MOESM4_ESM.zip › Source Data/Purified peptide data/Spectra/Peptide_3.jpg]

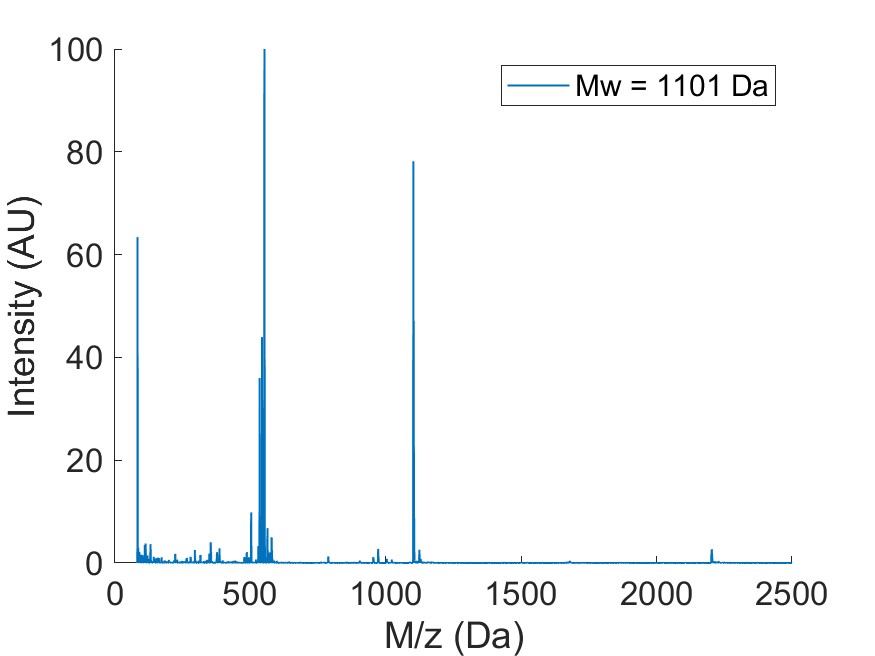

Supplement: Supplementary file 4 — Source data [file 41467_2023_42632_MOESM4_ESM.zip › Source Data/Purified peptide data/Spectra/Peptide_3ph.jpg]

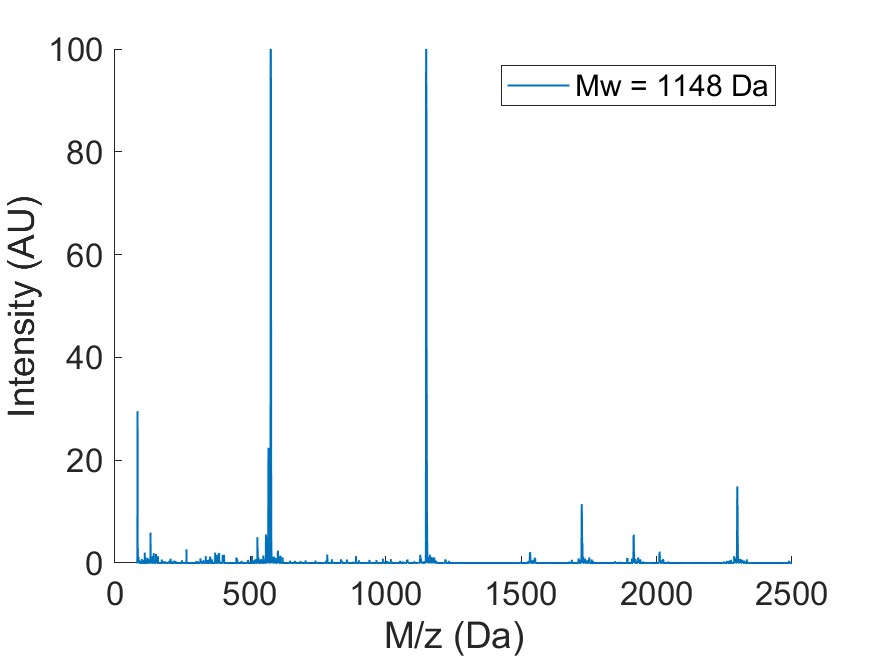

Supplement: Supplementary file 4 — Source data [file 41467_2023_42632_MOESM4_ESM.zip › Source Data/Purified peptide data/Spectra/Propgly3.jpg]

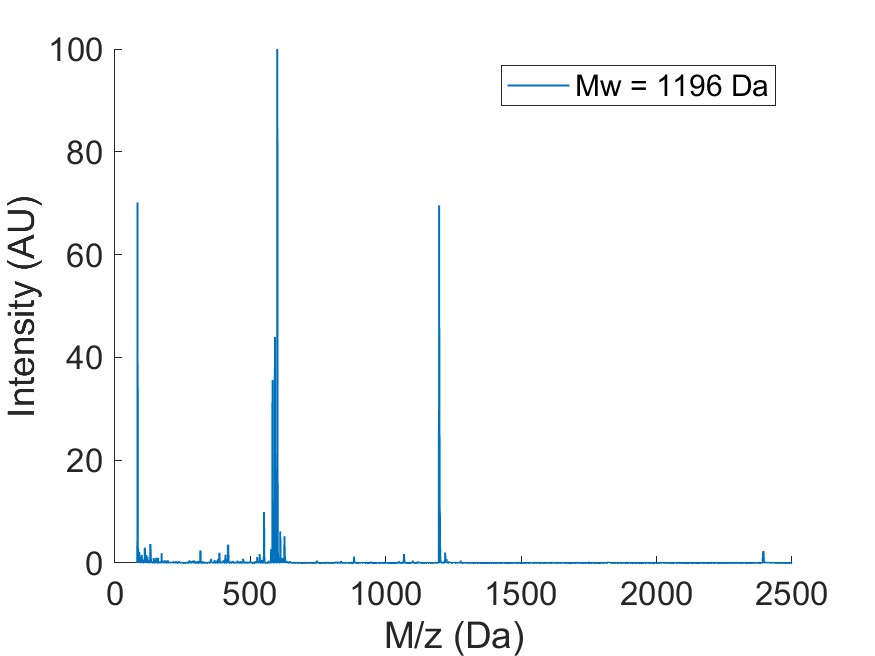

Supplement: Supplementary file 4 — Source data [file 41467_2023_42632_MOESM4_ESM.zip › Source Data/Purified peptide data/Spectra/Propgly3ph.jpg]

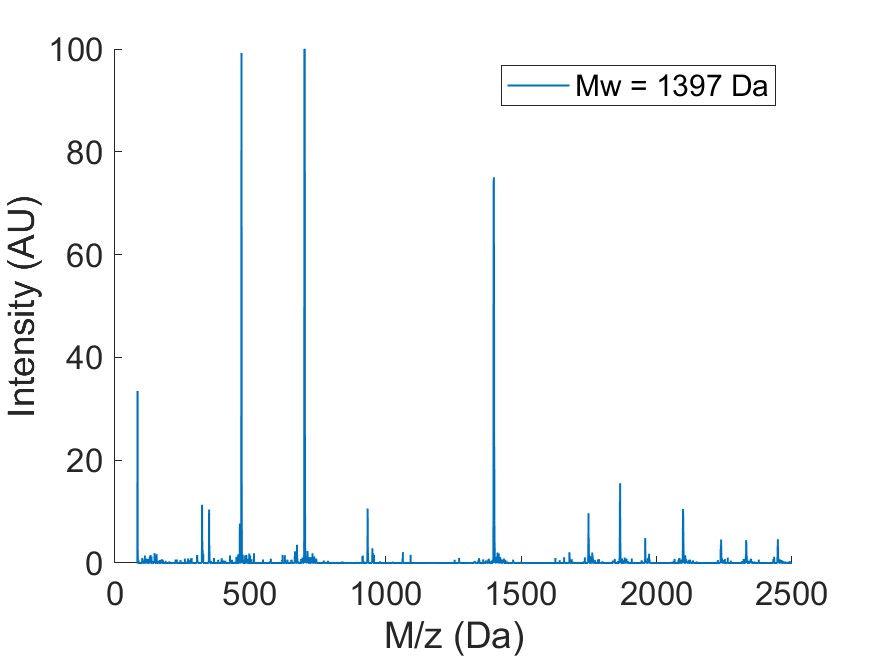

Supplement: Supplementary file 4 — Source data [file 41467_2023_42632_MOESM4_ESM.zip › Source Data/Purified peptide data/Spectra/Propgly8.jpg]

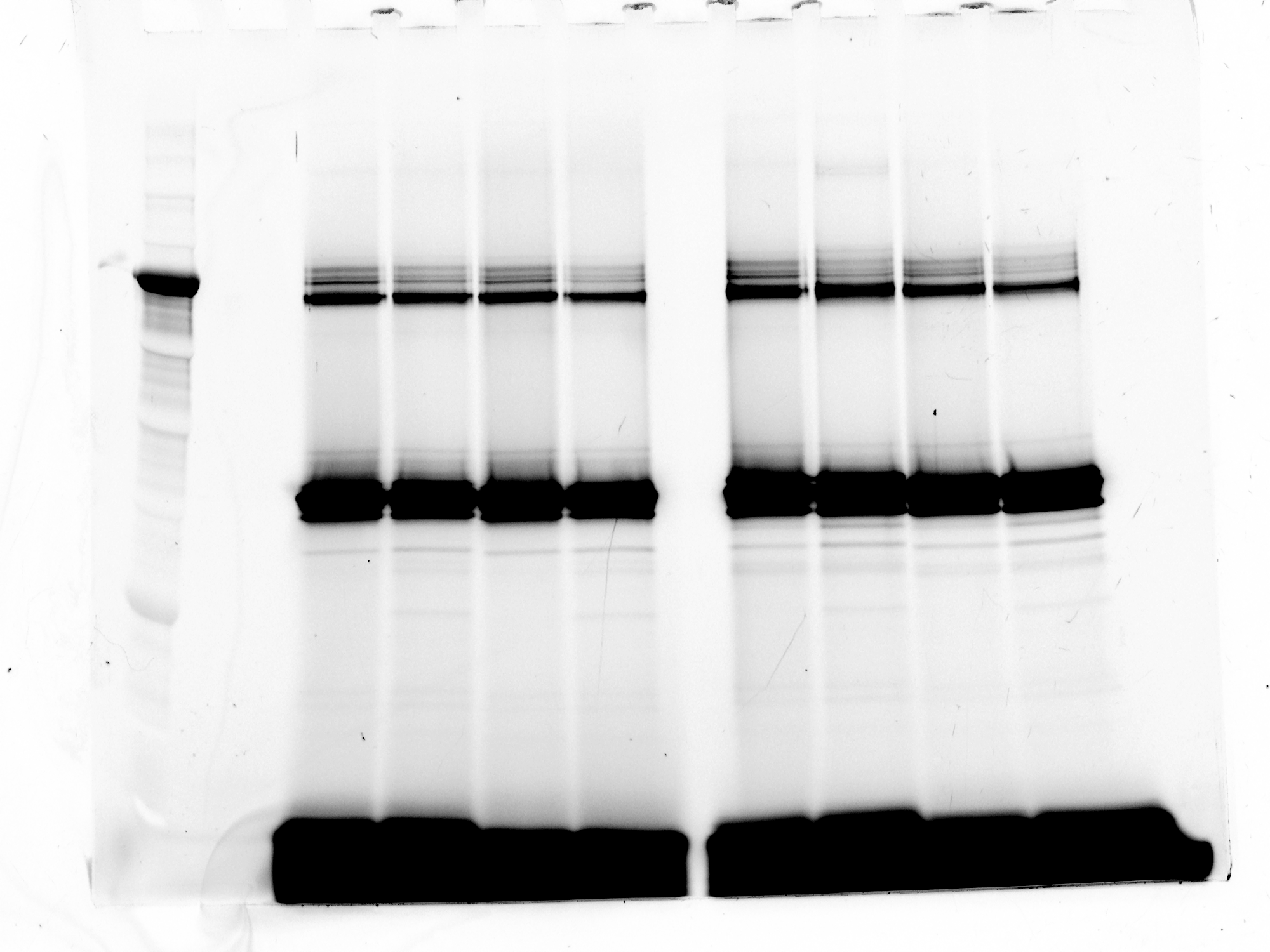

Supplement: Supplementary file 4 — Source data [file 41467_2023_42632_MOESM4_ESM.zip › Source Data/Supplementary figure 4/Panel D/Gel.tif]

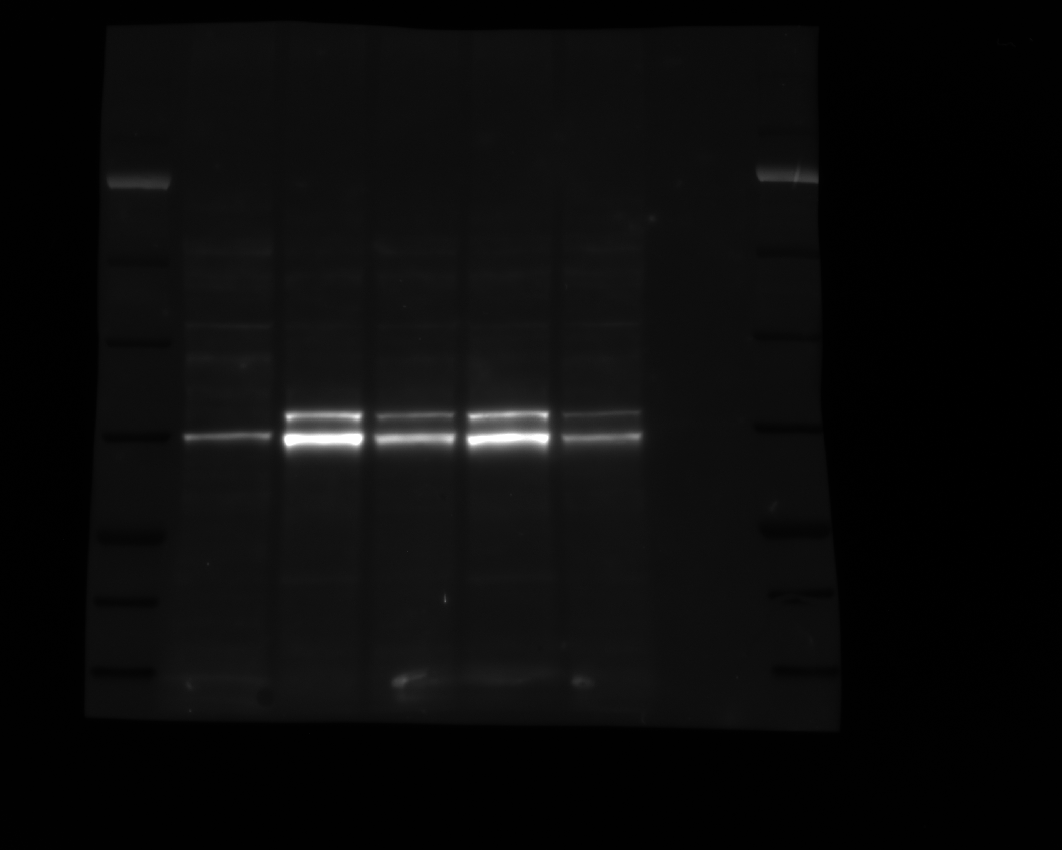

Supplement: Supplementary file 4 — Source data [file 41467_2023_42632_MOESM4_ESM.zip › Source Data/Supplementary figure 7/Panel A/Lysate/2023-07-11 London 09h35m42s(Alexa 546).tif]

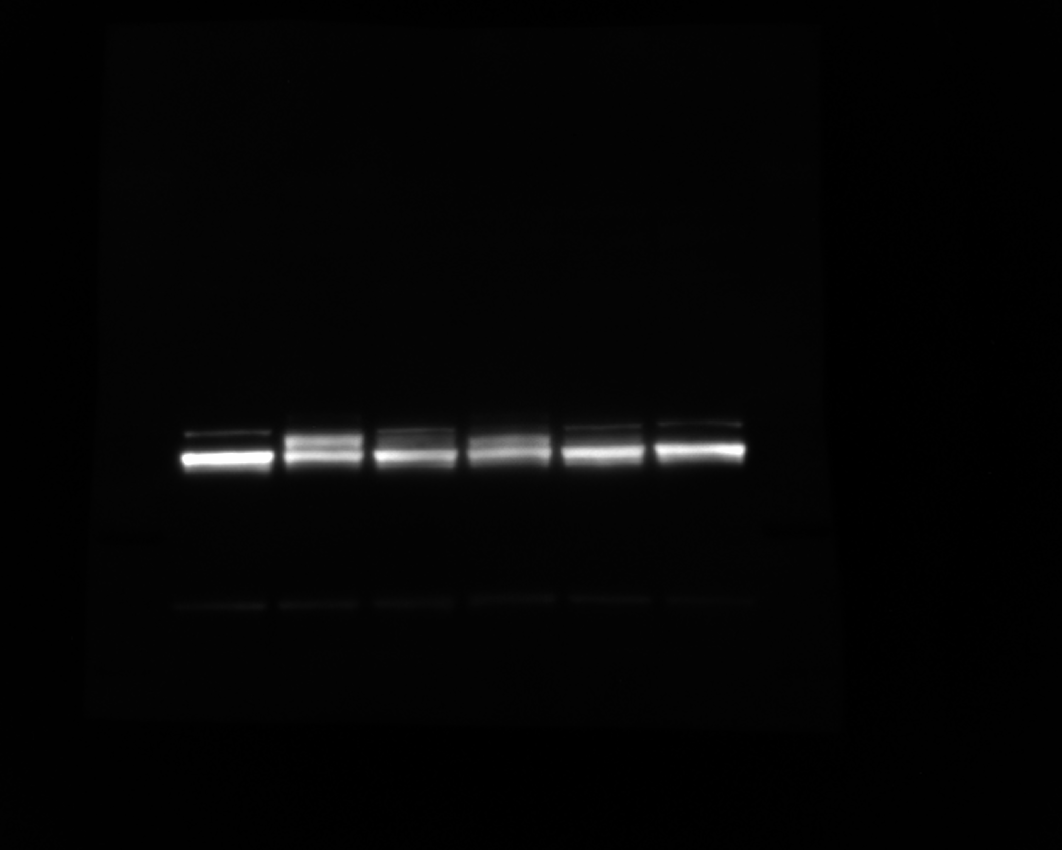

Supplement: Supplementary file 4 — Source data [file 41467_2023_42632_MOESM4_ESM.zip › Source Data/Supplementary figure 7/Panel A/Lysate/2023-07-11 London 09h35m42s(Chemiluminescence).tif]

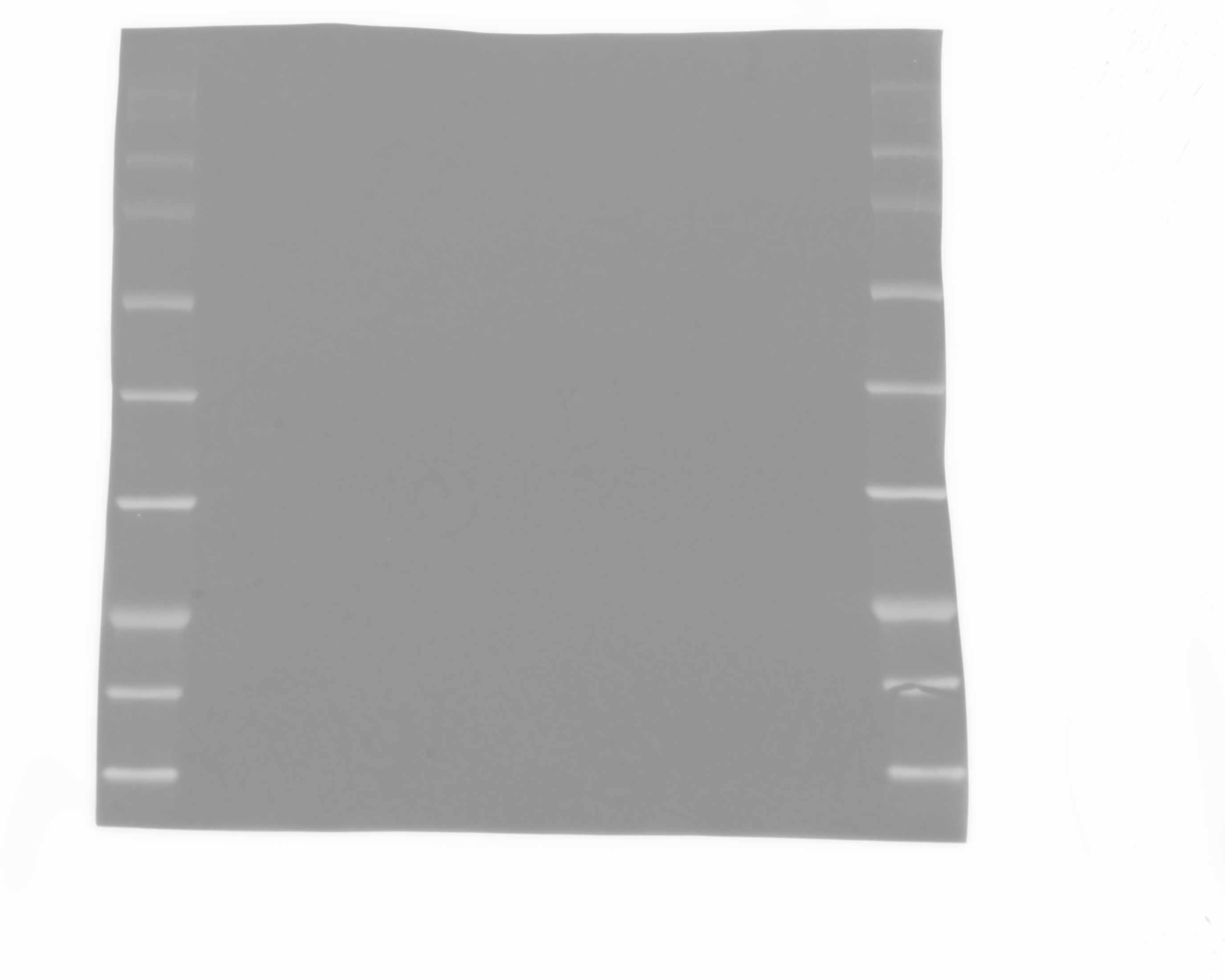

Supplement: Supplementary file 4 — Source data [file 41467_2023_42632_MOESM4_ESM.zip › Source Data/Supplementary figure 7/Panel A/Lysate/2023-07-11 London 09h35m42s(Colorimetric).raw16.tif]

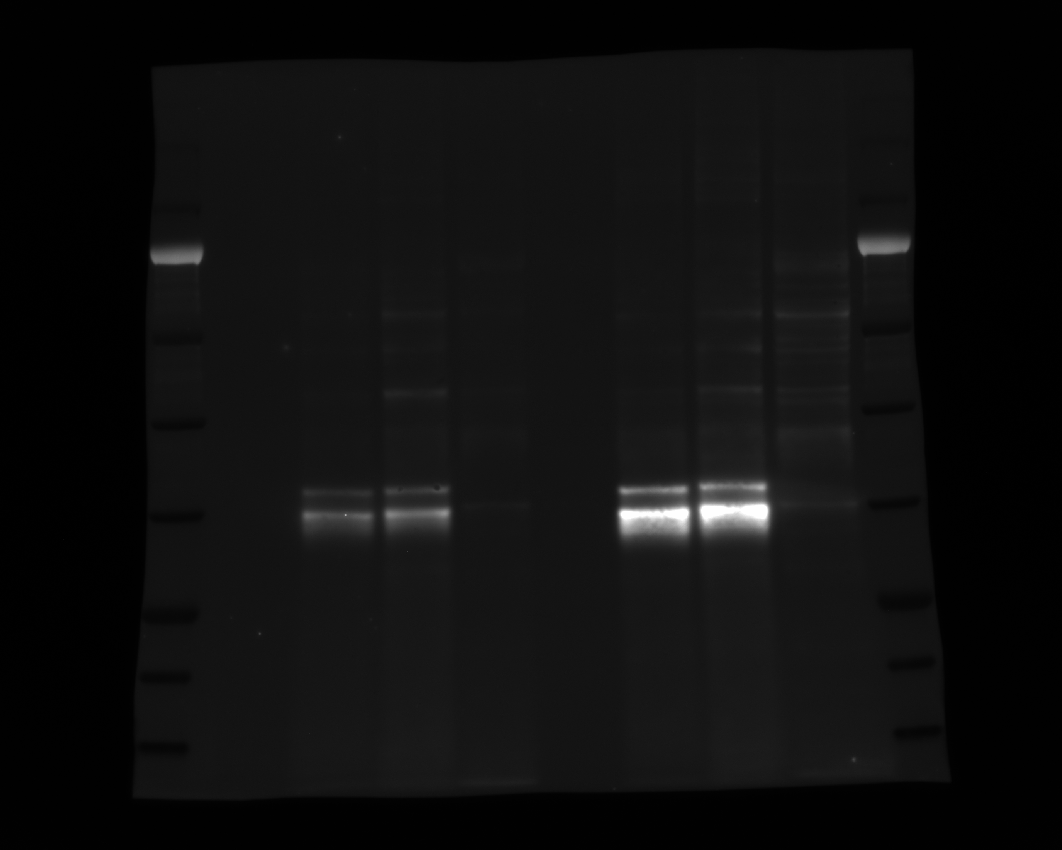

Supplement: Supplementary file 4 — Source data [file 41467_2023_42632_MOESM4_ESM.zip › Source Data/Supplementary figure 7/Panel A/Medium/2023-07-11 London 09h48m41s(Alexa 546).tif]

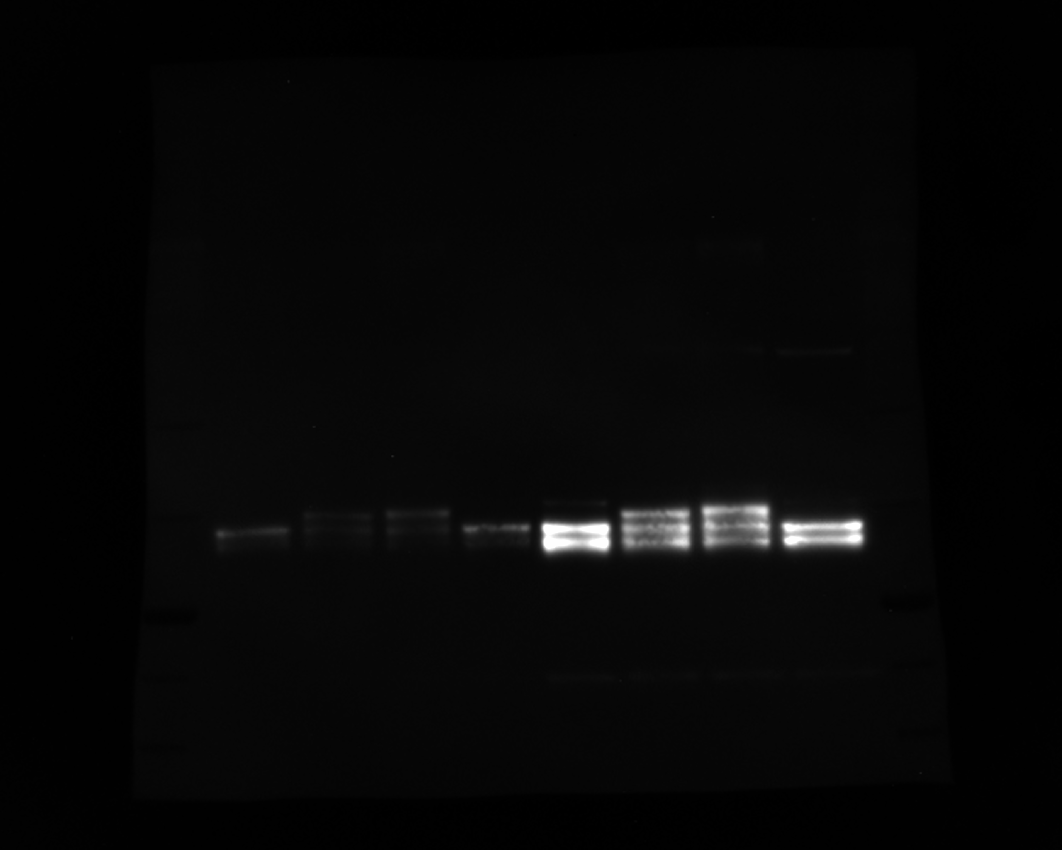

Supplement: Supplementary file 4 — Source data [file 41467_2023_42632_MOESM4_ESM.zip › Source Data/Supplementary figure 7/Panel A/Medium/2023-07-11 London 09h48m41s(Chemiluminescence).tif]

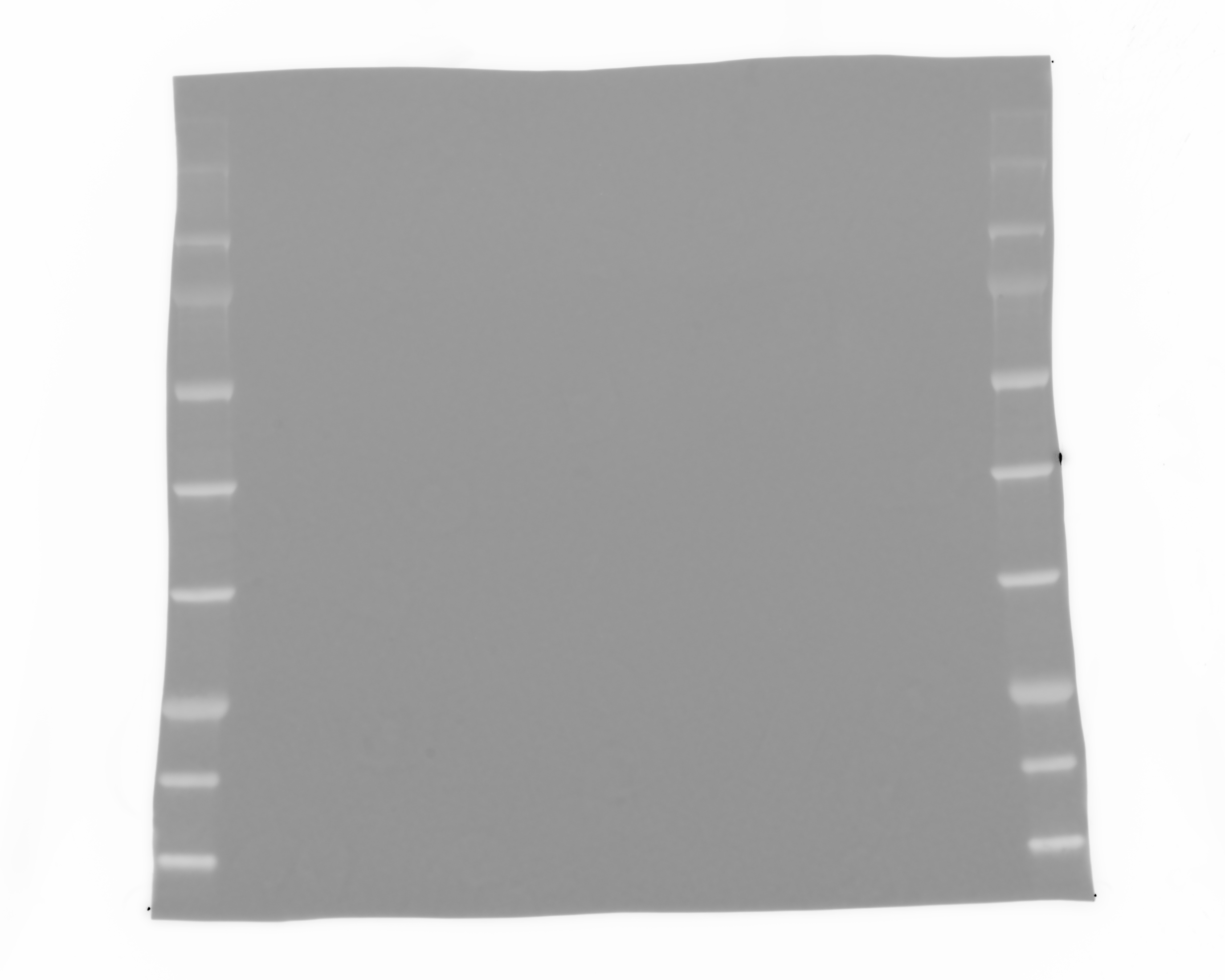

Supplement: Supplementary file 4 — Source data [file 41467_2023_42632_MOESM4_ESM.zip › Source Data/Supplementary figure 7/Panel A/Medium/2023-07-11 London 09h48m41s(Colorimetric).raw16.tif]

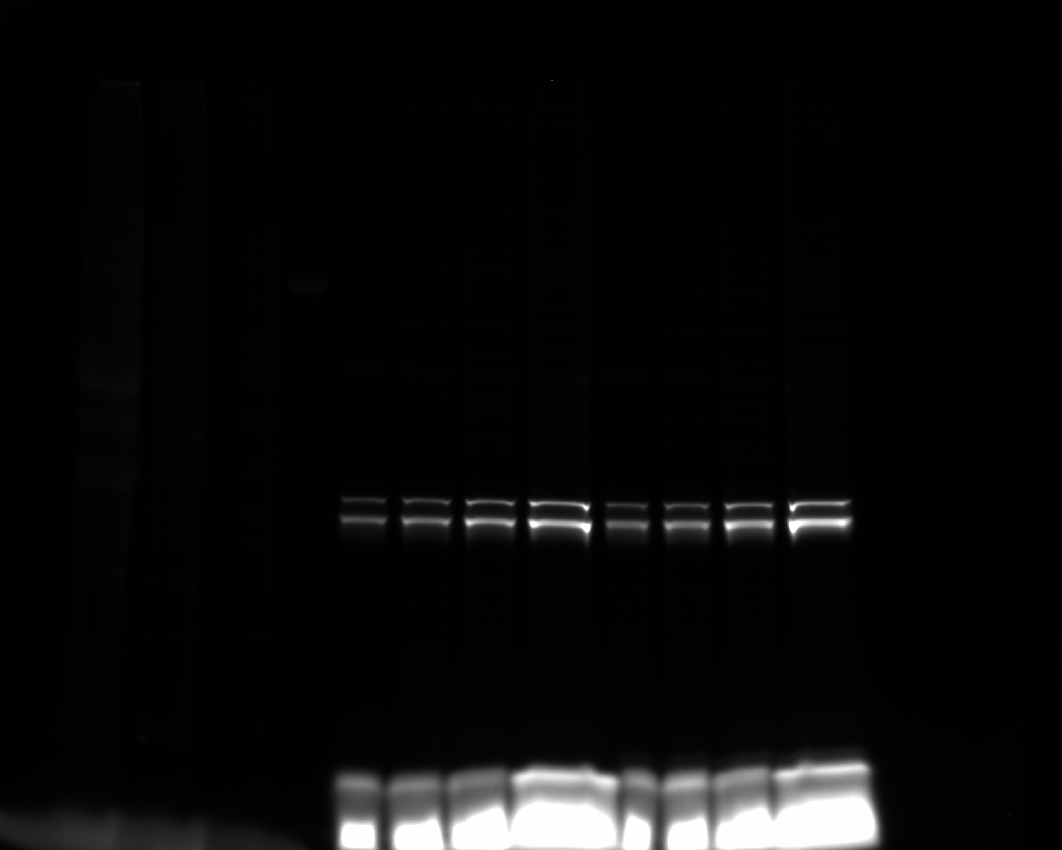

Supplement: Supplementary file 4 — Source data [file 41467_2023_42632_MOESM4_ESM.zip › Source Data/Supplementary figure 7/Panel B/2023-07-11 London 13h43m51s(Alexa 546).tif]

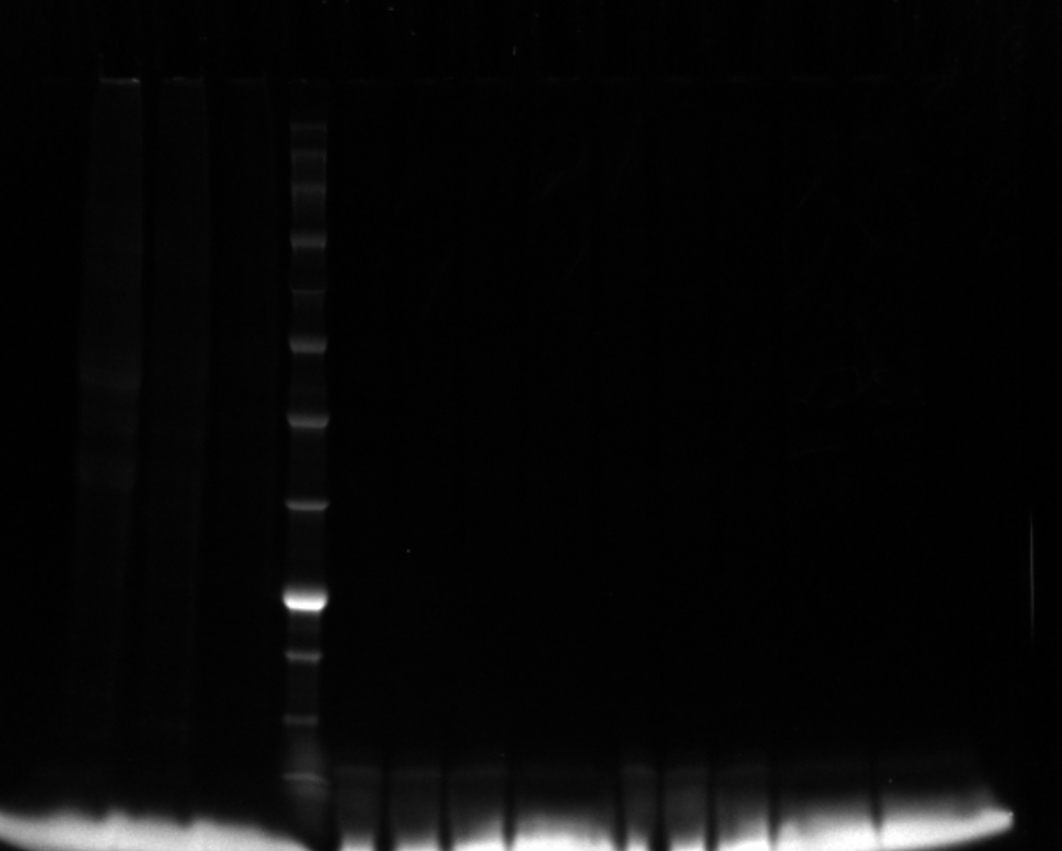

Supplement: Supplementary file 4 — Source data [file 41467_2023_42632_MOESM4_ESM.zip › Source Data/Supplementary figure 7/Panel B/2023-07-11 London 13h45m15s(Coomassie Blue).jpg]
